# Structural Insights into the Host–Guest Complexation between β-Cyclodextrin and Bio-Conjugatable Adamantane Derivatives

**DOI:** 10.3390/molecules26092412

**Published:** 2021-04-21

**Authors:** Jian-Wei Wang, Ka-Xi Yu, Xin-Yuan Ji, Hongzhen Bai, Wen-Hua Zhang, Xiurong Hu, Guping Tang

**Affiliations:** 1 Department of Chemistry, Zhejiang University, Hangzhou 310028, China; wjw126hz@zju.edu.cn (J.-W.W.); yukaxi@zju.edu.cn (K.-X.Y.); 3170101226@zju.edu.cn (X.-Y.J.); hongzhen_bai@zju.edu.cn (H.B.); 2 Department of Materials, Chemistry and Chemical Engineering, Soochow University, Suzhou 215123, China; whzhang@suda.edu.cn

**Keywords:** cyclodextrin, adamantane derivatives, bio-conjugation, host–guest interaction, crystal structure

## Abstract

Understanding the host–guest chemistry of α-/β-/γ- cyclodextrins (CDs) and a wide range of organic species are fundamentally attractive, and are finding broad contemporary applications toward developing efficient drug delivery systems. With the widely used β-CD as the host, we herein demonstrate that its inclusion behaviors toward an array of six simple and bio-conjugatable adamantane derivatives, namely, 1-adamantanol (adm-1-OH), 2-adamantanol (adm-2-OH), adamantan-1-amine (adm-1-NH_2_), 1-adamantanecarboxylic acid (adm-1-COOH), 1,3-adamantanedicarboxylic acid (adm-1,3-diCOOH), and 2-[3-(carboxymethyl)-1-adamantyl]acetic acid (adm-1,3-diCH_2_COOH), offer inclusion adducts with diverse adamantane-to-CD ratios and spatial guest locations. In all six cases, β-CD crystallizes as a pair supported by face-to-face hydrogen bonding between hydroxyl groups on C2 and C3 and their adjacent equivalents, giving rise to a truncated-cone-shaped cavity to accommodate one, two, or three adamantane derivatives. These inclusion complexes can be terminated as (adm-1-OH)_2_⊂CD_2_ (**1**, 2:2), (adm-2-OH)_3_⊂CD_2_ (**2**, 3:2), (adm-1-NH_2_)_3_⊂CD_2_ (**3**, 3:2), (adm-1-COOH)_2_⊂CD_2_ (**4**, 2:2), (adm-1,3-diCOOH)⊂CD_2_ (**5**, 1:2), and (adm-1,3-diCH_2_COOH)⊂CD_2_ (**6**, 1:2). This work may shed light on the design of nanomedicine with hierarchical structures, mediated by delicate cyclodextrin-based hosts and adamantane-appended drugs as the guests.

## 1. Introduction

Cyclodextrins (CDs) are a class of semi-natural cyclic molecular entities featuring five or more *α*-D-glucopyranoside units in a ring linked by α-1,4-glycosidic bonds [1,2,3,4,5,6]. Among this rich class of cyclic oligosaccharides, α-/β-/γ- CDs are widely studied as hosts for a variety of organic molecular guests because of their suitable size, amphiphilic structures, and good availability. Compared to other synthetic host systems, e.g., crown ethers [7,8,9], calixarenes [10,11,12], cucurbiturils [9,13,14], and pillararenes [15,16,17], CDs are less toxic and/or exhibit good water solubility and biocompatibility [11,18]. Their facile covalent modification also renders them highly promising candidates as functional delivery systems [19,20,21,22].

Aside from the covalent functionalization, the capability of pristine CDs as hosts to encapsulate a large number of organic species forms the basis of many practical applications in the pharmaceutical formulation [19,20,23,24], cosmetics [25], environmental remediation [26,27,28], agriculture, and food industry [29]. This originates from the unique hydrophobic inner cavity and hydrophilic outer space of CDs, with the latter also critical for imparting solubility to the otherwise less soluble organic species via inclusion [1,30,31]. These encapsulated species can be subsequently released on-demand with external stimuli, such as competitive solvent association, light-/heat-triggered conformation switch, and chemical stimuli (e.g., redox reactions) [19,20,23,32,33,34,35].

Intrigued by these highly favorable traits of CDs, a huge body of work has been done toward the efficient hierarchical catch-release systems through facile host–guest hybridization to harness the beneficial properties of both CDs and the guests [23,31,32,36,37]. The most widely studied host and guest pair feature β-CD and adamantane derivatives mainly due to their wide availability and good size match [23,38,39].

We have a keen interest in developing β-/γ-cyclodextrin encapsulated antiangiogenic drugs (sorafenib and regorafenib) to boost their bioavailability [31,40], as well as sophisticated systems based on polyethylenimine (PEI)-tethered CDs and adamantane-appended guests toward efficient drug delivery systems [34,41,42]. For example, we have recently reported that, by associating cleavable cationic PEI-tethered β-CD and an array of adamantane-appended polyethylene glycols (PEGs) carrying folate for active tumor targeting, nanosized supramolecular vehicles can be formed for in vitro and in vivo siRNA-Bcl2 delivery toward efficient gene therapy [43].

The host–guest interactions between β-CD with simple adamantane derivatives carrying bio-conjugatable functionalities (–OH, –NH_2_, –COOH, etc.) at the small molecular level may in part mirror those at the macromolecular level in the drug delivery systems. Further exploitation of these interactions may also provide us insight into efficient drug delivery systems with desirable drug loading efficacy and material stability. It is however surprising to note that, though an extensive array of inclusion complexes of β-CD have been reported, there is only a paucity of X-ray single-crystal structure studies of adamantane encapsulated β-CD [39,44,45], and systematic structural characterization of host–guest assemblies based on β-CD and adamantane derivatives is absent.

**Chart 1:**
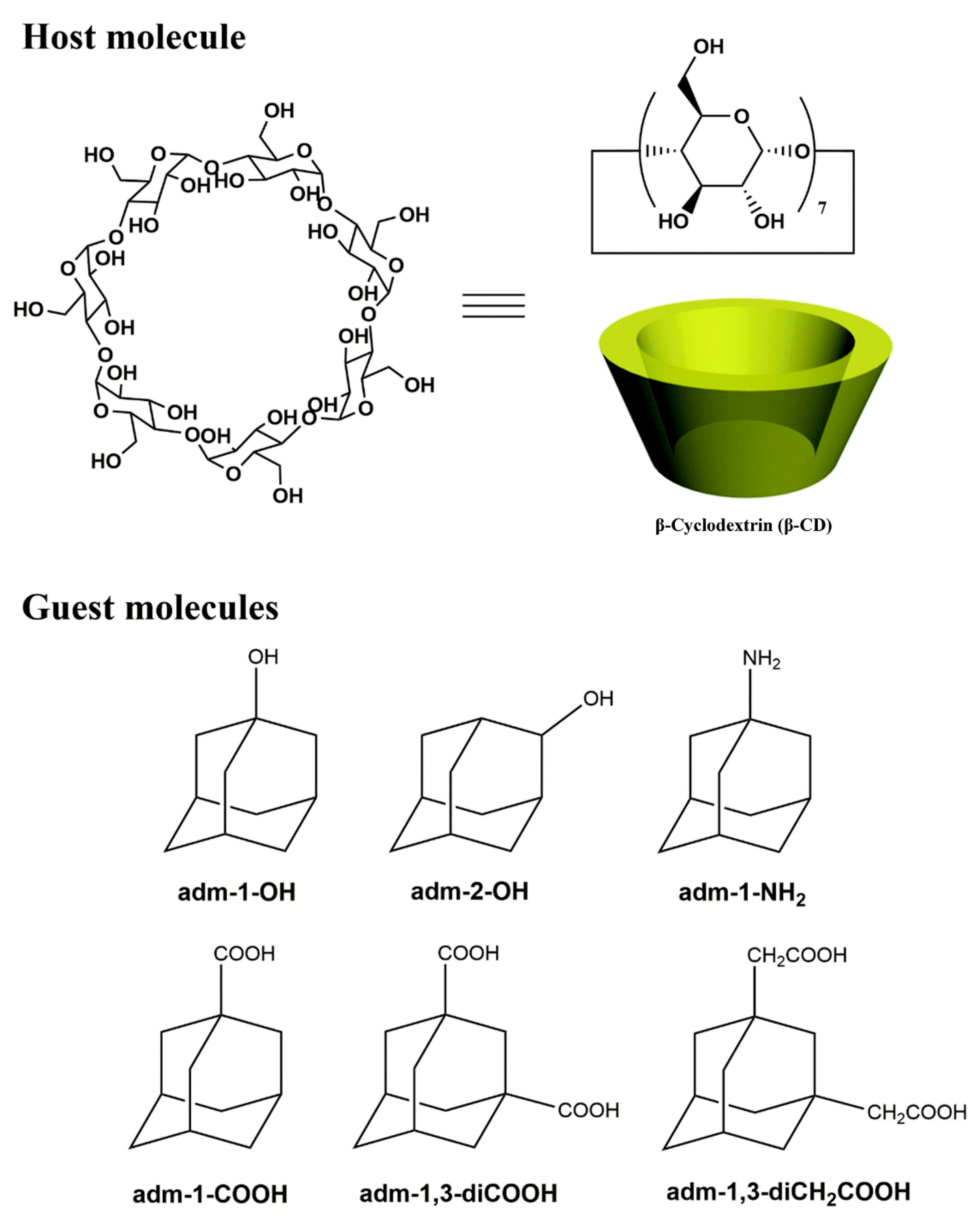
Chemical structures and cartoon illustration of β-CD and the adamantane derivatives.

We herein demonstrate that the hybridization of β-CD with simple bio-conjugatable adamantane derivatives, including 1-adamantanol (adm-1-OH), 2-adamantanol (adm-2-OH), adamantan-1-amine (adm-1-NH_2_), 1-adamantanecarboxylic acid (adm-1-COOH), 1,3-adamantanedicarboxylic acid (adm-1,3-diCOOH), an 2-[3-(carboxymethyl)-1-adamantyl]acetic acid (adm-1,3-diCH_2_COOH) (Chart 1), give rise to unanticipated adamantanamine-to-CD ratios ranging from 1:2 to 3:2 in the solid state, with diverse orientations of the functional groups to the β-CD host (while fully appreciating that the dimeric form of β-CD does not exist in some organic solvent, such as DMSO [46,47], and the number of guest species are not necessarily integrals, we use this datum format to show the possible location and orientation of the guest molecules). An array of six adamantane⊂CD hybrids are thus provided, comprising (adm-1-OH)_2_⊂CD_2_ (**1**, 2:2), (adm-2-OH)_3_⊂CD_2_ (**2**, 3:2), (adm-1-NH_2_)_3_⊂CD_2_ (**3**, 3:2), (adm-1-COOH)_2_⊂CD_2_ (**4**, 2:2), (adm-1,3-diCOOH)⊂CD_2_ (**5**, 1:2), and (adm-1,3-diCH_2_COOH)⊂CD_2_ (**6**, 1:2). In all these cases, β-CD crystallizes as a pair supported by face-to-face hydrogen bonding, between hydroxyl groups on C2 and C3 with their equivalents from adjacent glucopyranose units, giving rise to a truncated-cone-shaped cavity to accommodate adamantane derivatives to support one, two, or three adamantane-derivatives. We demonstrate that the size, geometry, and identity of functionalities, all contribute to the final structural outcome of these host–guest assemblies. This work may provide insight toward designing supramolecular delivery systems underpinned by CDs and adamantane derivatives.

## 2. Experimental Section

### 2.1. General

β-CD, 1-adamanol and 1-adamantanecarboxylic acid were purchased from Sigma-Aldrich (Shanghai, China), 1,3-adamantanedicarboxylic acid and 2-[3-(carboxymethyl)-1-adamantyl]acetic acid were purchased from Tokyo Chemical Industry (Tokyo, Japan), 2-adamanol was purchased from Adama-Beta Reagent Company (Shanghai, China), adamantan-1-amine was obtained from Energy Chemical Industry Company (Shanghai, China). All of the other chemicals were obtained directly from commercial sources and used as received. The two-dimensional ^1^H-^1^H nuclear Overhauser effect spectroscopy (NOESY) was recorded on a Bruker DRX-500 nuclear magnetic resonance (NMR) spectrometer (Bruker, Ettlingen, Germany), and chemical shifts (δ) referenced to the residual solvent peaks or internal standard TMS. The Fourier-transform infrared (FT-IR) spectra were measured on a Thermo Scientific Nicolet iS50 FT-IR spectrometer (Thermo Fisher Scientific Co., Waltham, MA, USA) as KBr disks (400–4000 cm^−1^). The TGA–DSC diagrams were performed on a TA DSC Q100 differential filtering calorimeter (TA Instruments, New Castle, DE, USA) at the heating rate of 10 °C·min^−1^ under a nitrogen stream of 50 cm^3^·min^−1^.

### 2.2. Preparation of β-CD and Adamantane Inclusion Complexes

β-CD and an equivalent amount of adamantane derivatives were introduced to an EtOH–H_2_O mixture with different volume ratios (3:7 for **1**; 2:8 for **2**; 3:7 for **3**; pure water for **4**; 4:6 for **5**; 4:6 for **6**). The formed mixtures were stirred at 80 °C for 2–3 h and smoothly cooled to room temperature. The crystals of each inclusion complex were obtained after ca 8 h as colorless parallelogram platelet crystals, which are significantly different from those of the pristine β-CD (colorless square-platelet crystals). The crystals were filtered and dried in air for subsequent characterizations.

### 2.3. ^1^H-NMR and FT-IR Characterization Data for the Adamantane Inclusion Complexes

*Characterization data for (adm-1-OH)_2_⊂CD_2_* (**1**):^1^H-NMR (500 MHz, DMSO-d_6_): δ 5.72 (d, *J* = 7.0 Hz, 7H), 5.66 (d, *J* = 2.0 Hz, 7H), 4.82 (d, *J* = 3.0 Hz, 7H), 4.45 (t, *J* = 5.5 Hz, 7H), 4.28 (s, 1H), 3.69–3.55 (m, 28H), 3.35–3.28 (m, 14H), 2.04 (s, 3H), 1.58 (d*, J* = 2.5 Hz, 6H), 1.54 (br, 6H). IR (KBr disk): 3411 (s), 2911 (m), 2850 (w), 1639 (w), 1454 (w), 1421 (w), 1381 (w), 1367 (w), 1337 (w), 1305 (w), 1246 (w), 1203 (w), 1158 (m), 1106 (m), 1084 (m), 1031 (s), 937 (w), 858 (w), 758 (w), 702 (w), 646 (w), 607 (w), 575 (w), 527 (w), 480 (w), 444 (w) cm^–1^.

*Characterization data for (adm-2-OH)_3_⊂CD_2_* (**2**):^1^H-NMR (500 MHz, DMSO-d_6_): δ 5.72 (d, *J* = 6.5 Hz, 7H), 5.66 (s, 7H), 4.82 (d, *J* = 3.0 Hz, 7H), 4.48 (d, *J* = 3.0 Hz, 1H), 4.45 (s, 7H), 3.66–3.55 (m, 29H), 3.33–3.28 (m, 14H), 2.06 (d, *J* = 12.0 Hz, 3H), 1.75–1.62 (m, 15H), 1.38 (d, *J* = 12.0 Hz, 3H). IR (KBr disk): 3412 (s), 2902 (m), 2852 (w), 1639 (w), 1458 (w), 1421 (w), 1380 (w), 1367 (w), 1337 (w), 1306 (w), 1248 (w), 1208 (w), 1159 (m), 1105 (m), 1083 (m), 1058(s), 1031 (s), 939 (w), 858 (w), 760 (w), 701 (w), 601 (w), 578 (w), 530 (w), 479 (w), 445 (w) cm^–1^.

*Characterization data for (adm-1-NH_2_)_3_⊂CD_2_* (**3**): ^1^H-NMR (500 MHz, DMSO-d_6_): δ 5.71 (s, 14H), 4.82 (d, *J* = 3.5 Hz, 7H), 4.48 (s, 7H), 3.66–3.55 (m, 28H), 3.34–3.28 (m, 14H), 2.00 (s, 3H), 1.59–1.52 (dd, *J1* = 21.5 Hz, *J2* = 12.0 Hz, 6H), 1.50 (d, *J* = 2.0 Hz, 6H). IR (KBr disk): 3381 (s), 2907 (m), 2849 (m), 1639 (w), 1452 (w), 1413 (w), 1366 (w), 1334 (w), 1303 (w), 1248 (w), 1202 (w), 1155 (m), 1105 (m), 1081 (m), 1030 (s), 1003 (s), 939 (w), 858 (w), 756 (w), 703 (w), 650 (w), 607 (w), 578 (w), 528 (w), 477 (w), 444 (w) cm^–1^.

*Characterization data for (adm-1-COOH)_2_⊂CD_2_* (**4**): ^1^H-NMR (500 MHz, DMSO-d_6_): δ 11.92 (br, 1H), 5.72 (d, *J* = 6.5 Hz, 7H), 5.67 (s, 7H), 4.82 (d, *J* = 3.0 Hz, 7H), 4.44 (t, *J* = 5.5 Hz, 7H), 3.68–3.56 (m, 28H), 3.33–3.28 (m, 14H), 1.97 (s, 3H), 1.79 (s, 6H), 1.66 (s, 6H). IR (KBr disk): 3381 (s), 2921 (s), 2953 (s), 1698 (m), 1644 (w), 1456 (w), 1414 (w), 1384 (w), 1371 (w), 1332 (w), 1300 (w), 1278 (w), 1245 (w), 1204 (w), 1155 (m), 1105 (m), 1080 (m), 1057 (s), 1030 (s), 1004 (s), 939 (m), 890 (w), 861 (w), 757 (w), 704 (w), 666 (w), 650 (w), 605 (w), 577 (w), 529 (w), 478 (w), 445 (w), 413 (w) cm^–1^.

*Characterization data for (adm-1,3-diCOOH)⊂CD_2_* (**5**): ^1^H-NMR (500 MHz, DMSO-d_6_): δ 12.11 (br, 2H), 5.73 (d, J = 7.0 Hz, 7H), 5.68 (d, *J* = 2.0 Hz, 7H), 4.83 (d, *J* = 3.0 Hz, 7H), 4.46 (t, *J* = 5.5 Hz, 7H), 3.67–3.55 (m, 28H), 3.37–3.28 (m, 14H), 2.06 (s, 2H), 1.84 (s, 2H), 1.78–1.69 (dd, *J1* = 30.5 Hz, *J2* = 11.0 Hz, 8H), 1.61 (s, 2H). IR (KBr disk): 3414 (s), 2930 (s), 2857 (m), 1705 (m), 1643 (w), 1455 (w), 1415 (w), 1383 (w), 1373 (w), 1335 (w), 1299 (w), 1273 (w), 1247 (w), 1202 (w), 1157 (m), 1106 (m), 1080 (m), 1050 (s), 1029 (s), 1004 (s), 941 (m), 890 (w), 861 (w), 757 (w), 705 (w), 655 (w), 607 (w), 577 (w), 530 (w), 477 (w), 445 (w), 412 (w) cm^–1^.

*Characterization data for (adm-1,3-diCH_2_COOH)⊂CD_2_* (**6**): ^1^H-NMR (500 MHz, DMSO-d_6_): δ 11.83 (br, 1H), 5.69 (br, 12H), 4.83 (d, *J* = 3.0 Hz, 7H), 3.68–3.55 (m, 28H), 3.37–3.29 (m, 14H), 1.99 (s, 2H), 1.97 (s, 4H), 1.54–1.43 (m, 12H). IR (KBr disk): 3382 (s), 2907 (s), 2849 (m), 1701 (m), 1644 (w), 1448 (w), 1410 (w), 1366 (w), 1329 (w), 1295 (w), 1273 (w), 1247 (w), 1202 (w), 1158 (m), 1104 (m), 1081 (m), 1056 (s), 1031 (s), 1002 (s), 942 (m), 861 (w), 758 (w), 706 (w), 659 (w), 642 (w), 609 (w), 578 (w), 531 (w), 481 (w), 447 (w), 413 (w) cm^–1^.

### 2.4. X-ray Crystal Structure Determinations

Single crystals of **1** and **2** were analyzed on a Bruker D8 Venture diffractometer (Bruker, Karlsruhe, Germany) using liquid Ga-Kα irradiation (λ = 1.34139 Å), while the crystal data for other inclusion complexes were respectively recorded on Rigaku R-AXIS-RAPID (**3**, **4**, Rigaku, Tokyo, Japan), Agilent Xcalibur and Gemini (**5**, Agilent Technologies, Yarnton, UK), and Bruker Apex-II (**6**, Bruker, Karlsruhe, Germany) diffractometers with Mo-Kα (λ = 0.71073 Å) radiation. The appropriate absorption correction (multi-scan) was also applied [48,49,50]. All of the crystal structures were solved by direct methods and refined on F^2^ by full-matrix least-squares techniques with the SHELXTL-2016 program [50].

For **2**, the occupancy factors for one adm-2-OH were fixed at 0.5 to obtain reasonable thermal factors. The other adm-2-OH lies on a special position of higher symmetry than the molecule can possess. It was then treated as a spatial disorder but applying PART –1 and PART 0 in the .ins file with the site occupation factors changed to 0.25 for the atoms. Similarly, for **3**, the occupancy factors for the three adm-1-NH_2_ were fixed at 0.5 to obtain reasonable thermal factors. The other adm-1-NH_2_ lies in a special position of higher symmetry than the molecule can possess. It was then treated as a spatial disorder but applying PART –1 and PART 0 in the .ins file with the site occupation factors changed to 0.10 for the atoms. For **5**, the encapsulated dicarboxylic acid displayed conformational disorder with their relative ratio of 0.49:0.51 refined for the disordered domains. For **1**−**6**, the coordinates of the hydrogen atoms on the –OH groups were calculated by Calc-OH program in WinGX suite [51], their O–H distances were further restrained to O–H = 0.83 Å and thermal parameters constrained to U_iso_(H) = 1.2 U_eq_(O). For **1**−**5**, a large amount of spatially delocalized electron density in the lattice was found which were ascribed to the presence of water solvates. The solvent contribution was then modeled using SQUEEZE in the Platon program suite [52]. Specifically for **6**, a stable shape of adm-1,3-diCH_2_COOH cannot be modeled during the refinement probably due to the weak electron densities, and the adm-1,3-diCH_2_COOH guest is also treated as solvates and removed together with H_2_O using SQUEEZE (247e in total: 84e for adm-1,3-diCH_2_COOH and 163e for H_2_O) [53].

Crystallographic data have been deposited with the Cambridge Crystallographic Data Center (CCDC) as supplementary publication numbers 2053567–2053572. These data can be obtained free of charge from the Cambridge Crystallographic Data Centre via www.ccdc.cam.ac.uk/data_request/cif or from the Supporting Information. A summary of the key crystallographic data are listed in Table 1.

### 2.5. Isothermal Titration Calorimetry (ITC) Measurements

The ITC experiments were performed on a VP–ITC (MicroCal, Inc., Northampton, MA, USA) under atmospheric pressure and at 25.0 °C in 5% DMSO-water solution (pH 7). The β-CD and adamantane derivatives in **4** and **5** were completely dissolved, giving the stability constants (K) and the corresponding thermodynamic parameters. Specifically, a solution of β-CD in a 0.250 mL syringe was sequentially injected with stirring at 300 rpm into a solution of adamantane derivatives in the sample cell (1.423 mL volume). The concentrations of β-CD and adamantane derivatives were used as 0.1 and 1 mM, respectively. All of the thermodynamic parameters reported in this work were obtained by using the “one set of binding sites” model [54,55].

## 3. Results and Discussion

### 3.1. Synthesis and Spectroscopic Characterization

The inclusion complexes of (adm-1-OH)_2_⊂CD_2_ (**1**), (adm-2-OH)_3_⊂CD_2_ (**2**), (adm-1-NH_2_)_3_⊂CD_2_ (**3**), (adm-1-COOH)_2_⊂CD_2_ (**4**), (adm-1,3-diCOOH)⊂CD_2_ (**5**), and (adm-1,3-diCH_2_COOH)⊂CD_2_ (**6**) can be readily prepared by heating equivalent β-CD and respective guest molecules in EtOH/H_2_O mixed solvent at 80 °C. The successful formation of inclusion complexes can be readily distinguished from the change of crystal shape from the square-platelet of pristine β-CD to parallelogram-shaped crystals, regardless of the crystal systems in which these inclusion complexes crystallize. The observation of crystal shape change also suggests that the conversion yield of the inclusion complexes are high but incomplete.

As depicted in Figures S1–S12 (Supplementary Material), a comparison of the ^1^H-NMR spectra between the inclusion complexes of **1**–**6** with that of the free β-CD indicates the successful incorporation of the adamantane-based guest molecules [56]. ^1^H-NMR also suggested the adamantane-to-β-CD ratios of approximately 1:1 for all these complexes (Supplementary Figures S1–S6). The stoichiometric inconsistency between the ^1^H-NMR results and that described from the X-ray single-crystal analysis (elaborated below) can be manifold. The crystallization process usually gives host–guest complexes with a statistical mixture of the guest molecules. The host-to-guest ratio of one single crystal thus does not represent that in the bulk sample. Besides, for the characterization of inclusion species, X-ray diffraction itself can also create bias during the experiment due to its scattering by the hosts, leading to a weaker electron density of the guest than it ought to be. Thus, though we observed nearly homogenous single crystals of parallelogram shape, these single crystals are estimated to contain significantly fewer adamantane-based guests due to the insufficient encapsulation.

Notably, although the ^1^H-NMR suggests 1:1 host–guest ratio for all these inclusion complexes in DMSO solution, only limited chemical shifts were also observed as demonstrated (Supplementary Figures S1–S12 and Tables S1–S12). This point to the dissociation of the guest molecules in DMSO solutions [46,47], likely due to the competitive inclusion by the DMSO solvate that is dominant.

The two-dimensional ^1^H-^1^H nuclear Overhauser effect spectroscopy (NOESY) also indicates that there are only some weak interactions between the β-CD host and the adamantane derivatives, further suggest very limited guest inclusion (Supplementary Figures S13–S18). Nevertheless, from Supplementary Figures S13–S18, it is evident that the correlations between H6/H3 of adamantane backbone and interior protons of β-CD (H5) exist. 

**Figure 1:**
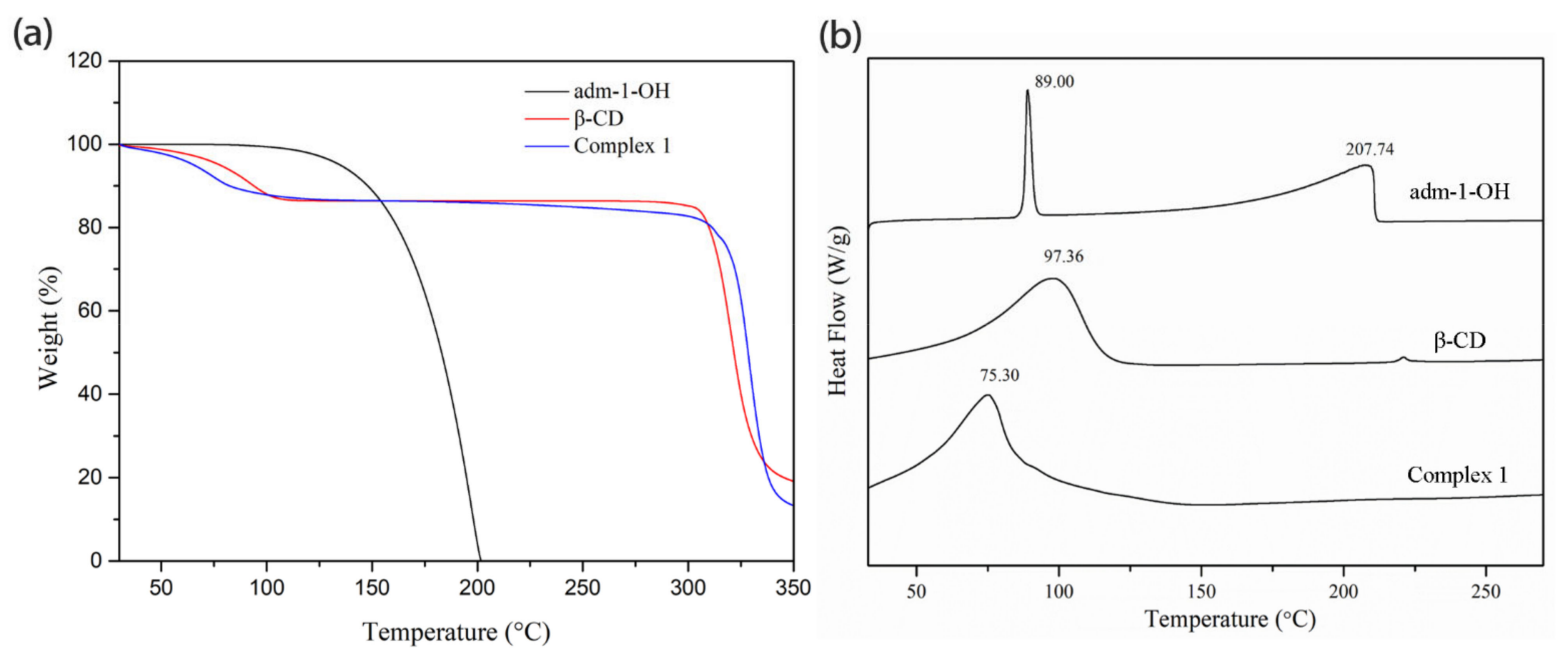
(**a**) TG traces of adm-1-OH, β-CD and **1**. (**b**) A comparison of the DSC-TGA curves of **1** and its subcomponents β-CD and adm-1-OH, showing the melting point alteration upon complex formation.

To investigate the thermal stabilities of these inclusion complexes, thermogravimetric and differential scanning calorimetry (TG-DSC) analyses were performed (Figure 1 and Supplementary Figures S19–S23). For **1**–**4**, the disappearance of the melting peaks of the adamantane guests coincides with the peak alteration of β-CD, indicative of the successful insertion of the guest molecules inside the macrocyclic cavity [56,57,58]. For **5** and **6**, the melting peak of the adamantane guests shifts to lower temperature and are also indicative of loss of the drug crystallinity and thus successful encapsulation. Thermogravimetric analysis (TGA, Figure 1a and Figures S19–S23, Supplementary Material) for **1**–**6** indicated that these inclusion complexes dehydrated at around 100 °C, and plateaued until around 300 °C, upon which the decomposition of these species commences.

### 3.2. Isothermal Titration Calorimetry (ITC) Analysis of **4** and **5**

**Figure 2:**
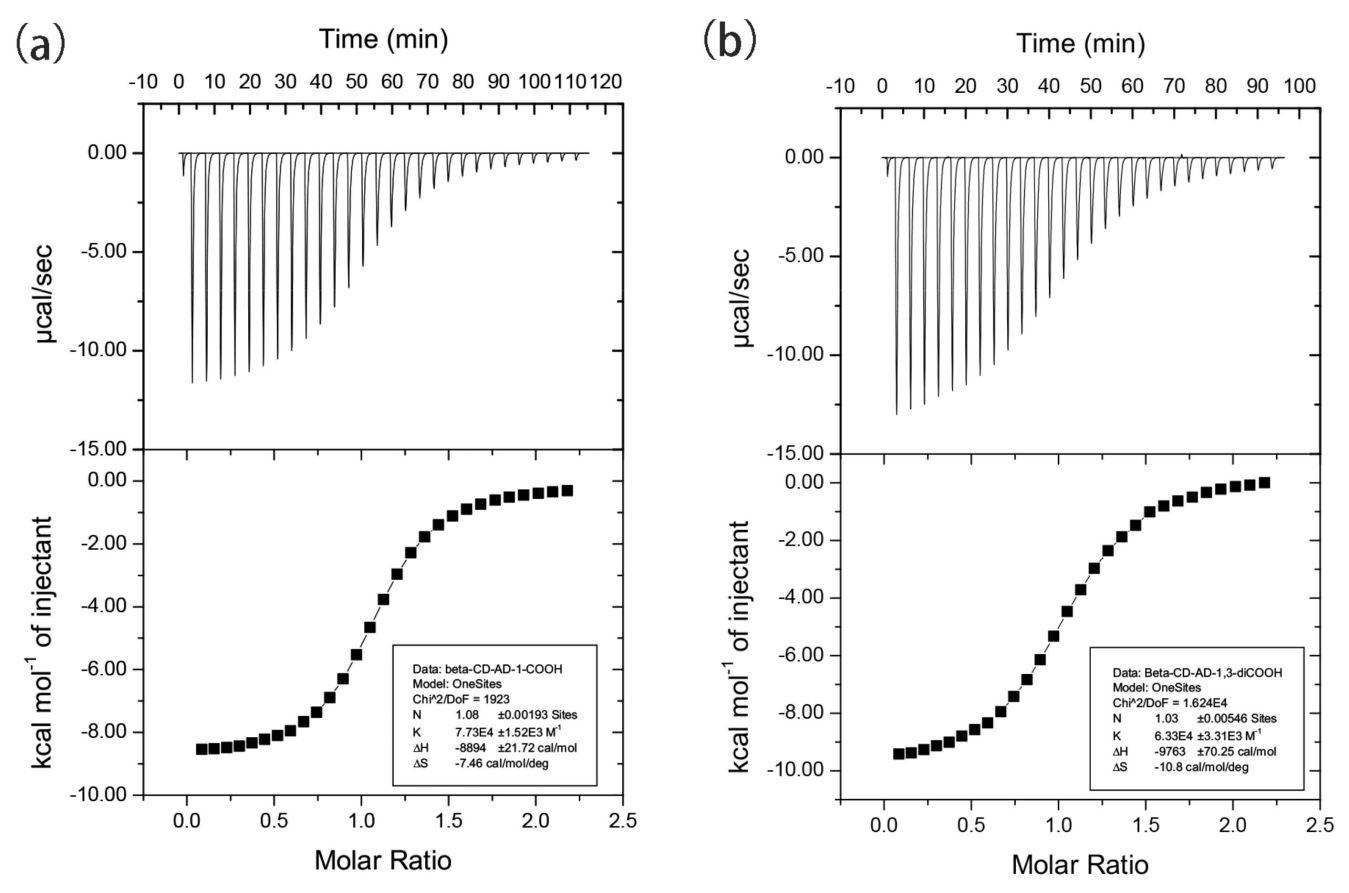
(**a**) The ITC isotherm for the titration of β-CD (1.0 mM) into a solution of adm-1-COOH (0.1 mM), in 5% DMSO-water solution at 25 °C. (**b**) The ITC isotherm for the titration of β-CD (1.0 mM) into a solution of adm-1,3-diCOOH (0.1 mM), in 5% DMSO-water solution at 25 °C.

To assess the thermodynamics of complexation, ITC was performed using a DMSO-water (5:95) solution at 25 °C. A one-step binding model was applied to generate an apparent binding constant (K) for the 1:1 inclusion complex for adm-1-COOH (**4**) and adm-1,3-diCOOH) (**5**) with β-CD, with a large ΔH value of −8894 cal·mol^−1^ and −9763 cal·mol^−1^, and relatively small ΔS value of −7.46 cal mol^−1^·°C^−1^ and −10.8 cal·mol^−1^·°C^−1^. Respective K values of 7.7 × 10^4^ M^−1^ and 6.3 × 10^4^ M^−1^ were accordingly derived (Figure 2). These results indicate that the interactions between **4** and **5** with β-CD tend to be enthalpically driven [59]. These K values observed herein are comparable to or higher than those inclusion complexes based on adamantanecarboxylate (deprotonated form of adm-1-COOH) with β-CD, γ-CD and *α*-CD (2 × 10^4^ M^−1^, 3 × 10^3^ M^−1^, and 1.4 × 10^2^ M^−1^, respectively) [4,60]. The association constant of adm-1-COOH with β-CD varies at different pHs, giving K value of 3.0 × 10^5^ M^−1^, 4.0 × 10^4^ M^−1^, and 1.8 × 10^4^ M^−1^ at pH 4.05, 7.2, and 8.5, respectively, were also reported [3]. Unfortunately, the satisfying ITC data acquisition for **1**, **2**, **3**, and **6** fails due to the low water solubility of these guest molecules.

### 3.3. Single-Crystal Structure Analysis of **1**–**6**

There are several typical space groups, such as *P*1, *C*2, *P*2_1_, or *C*222_1_ [61,62,63], and different crystal packing modes, including channel (CH), screw channel (SC), intermediate (IM), and chessboard (CB) for β-CD-based inclusion compounds [64,65]. According to this classification, the β-CD-based dimers crystallize in the *C*2, *P*2_1,_ and *C*222_1_ space groups are always stacked in the CH, SC, and CB modes. In contrast, those crystallize in the *P*1 space group stack either in the CH mode if their cell dimensions are all about 15.5 Å, or in the IM mode if one of the cell dimensions is more than 17 Å and the two others being about 15.5 Å. Complexes **1–6** well-fit into these categories, as listed in Table 2.

**Figure 3:**
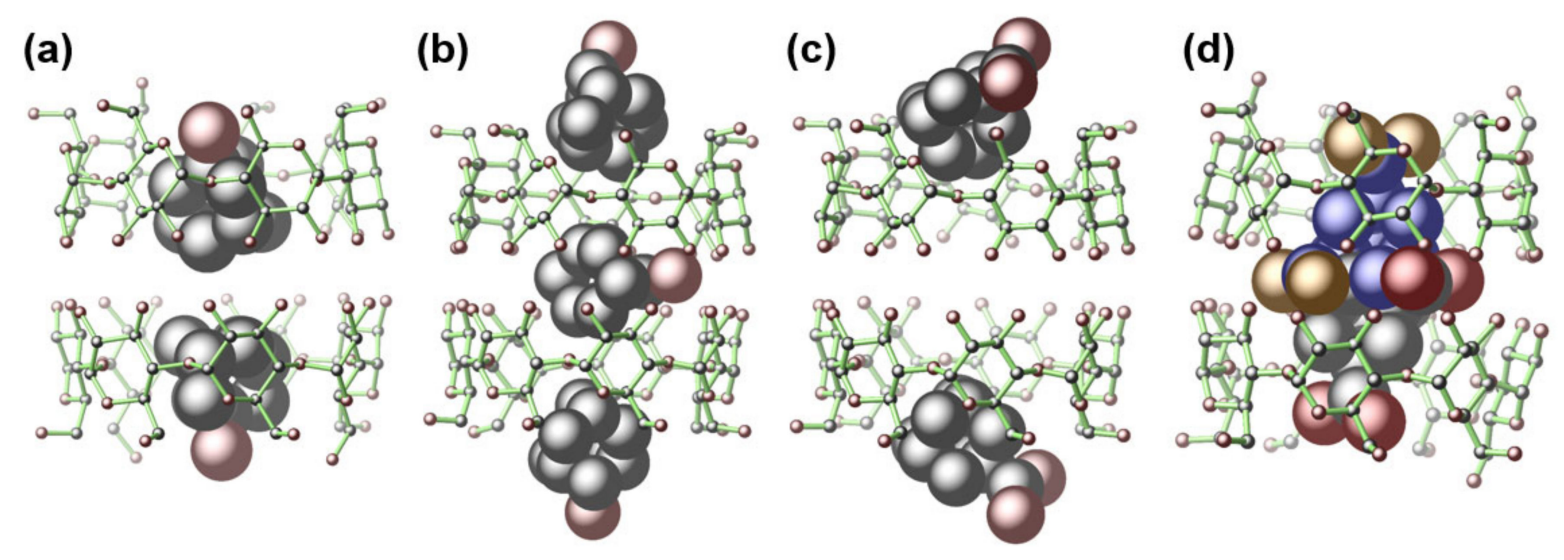
The X-ray crystal structures of **1** (**a**), **2** (**b**), **4** (**c**), and **5** (**d**) showing different adamantane-to-β-CD ratios and diverse guest orientations in the inclusion complexes. The guests are presented as a space-filling model. The hydrogen atoms are omitted for clarity. Color codes: O (brown-red), C (black). For **5**, the two disordered parts of adm-1,3-diCOOH are represented by blue (for C) and brown (for O), as well as gray (for black) and brown-red (for O).

**Figure 4:**
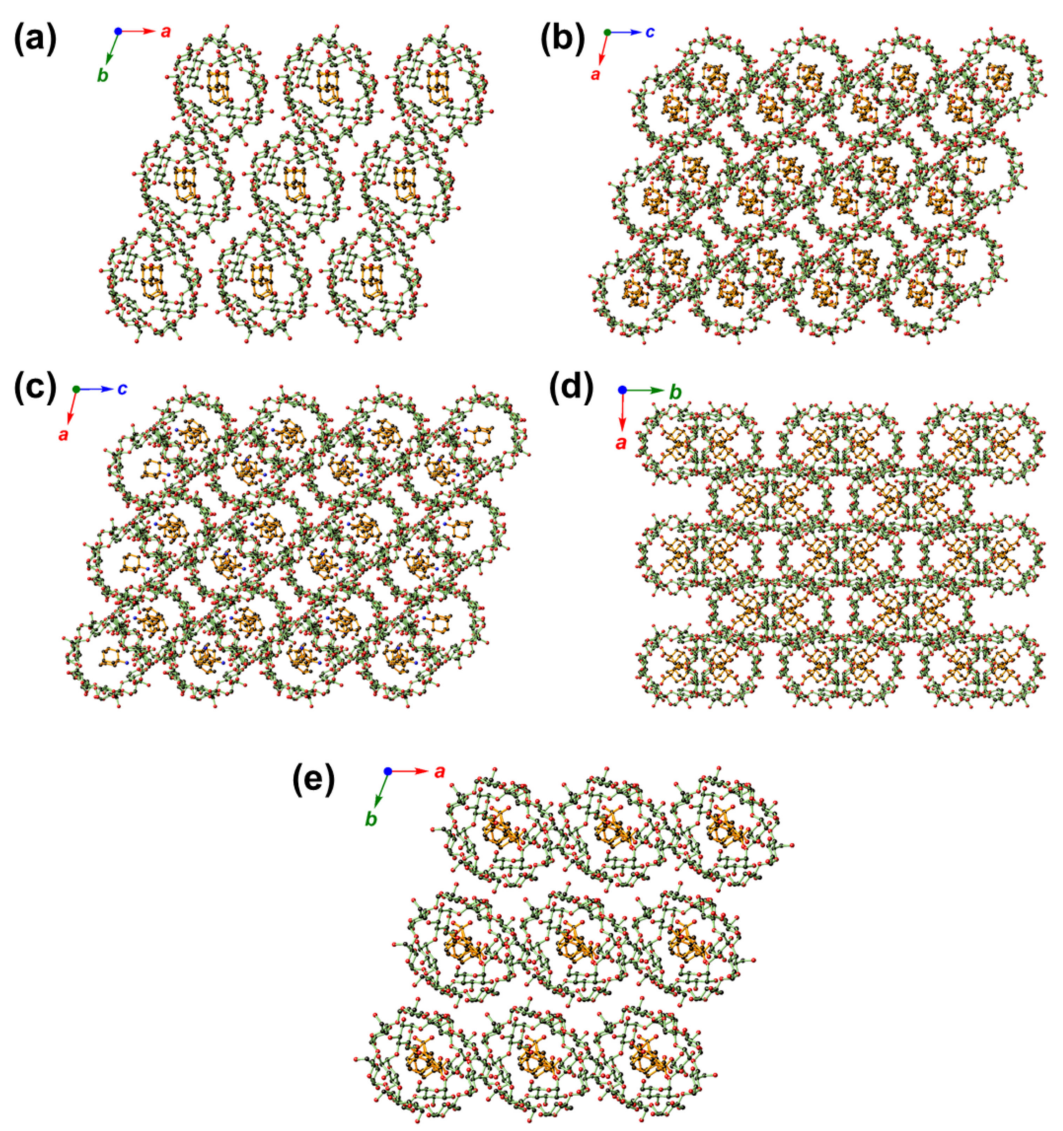
The crystal packing diagrams of **1** ((**a**); along *c* direction), **2** ((**b**); along *b* direction), **3** ((**c**); along *b* direction), **4** ((**d**); along *c* direction), and **5** ((**e**); along *c* direction), showing similarities and differences in their packing patterns. Color codes: O (red), N (blue), and C (gray).

As depicted in Figure 3, in the structures of **1**, **2**, **4**, and **5**, the β-CD host exclusively forms a pair via the hydrogen bonding interactions between the –OH groups on C2/C3 with their equivalents (symmetry codes: *x*, *y*, *z* for **1**; *x*, –*y* – 1, –*z* – 2 for **2**; –*x +* 1, *y*, –*z* + 3/2 for **3**; *x +* 1/2, *y +* 1/2, *z* for **4**; *x*, *y*, *z* for **5**; *x*, *y*, *z* for **6**) to give a truncated-cone-like cavity. Such dimeric form dominates the host–guest complexes of β-CD with very limited exceptions [66,67,68,69,70]. As shown in Figure 3a, a pair of adm-1-OH in the cavity of **1** features a back-to-back conformation. Complexes **2**, **3**, and **4** share similar cell parameters (Table 1), guest inclusion patterns (Figure 3b,c and Supplementary Figure S24), as well as the crystal packing diagrams (Figure 4b–d). The β-CD pairs in **2**/**3** host three bulky adm-2-OH/adm-1-NH_2_ guests, of which two are associated with the β-CD pairs on the surface, while the third one is deeply buried inside of the cavity supported by the β-CD pair, and adopted two symmetry-related orientations (symmetry codes: *x*, –*y* – 1, –*z* – 2 for **2**; –*x +* 1, *y*, –*z* + 3/2 for **3**). Notably, all these guest molecules are ordered (except the sandwiched guest in **2** and **3**) within the cavity, probably induced by the asymmetric micro-environment of the cavity [71,72,73].

The slight size increase from adm-2-OH (in **2**)/adm-1-NH_2_ (in **3**) to adm-1-COOH [74,75] resulted in a 2:2 inclusion crystal of **4**. With an identical β-CD-adamantane ratio to that of **1** with deeply buried adm-1-OH guests, the two adm-1-COOH guest in **4** are flanked on the two sides of the β-CD pair, leading to the generation of space with the potential to host additional guest with suitable size. It is notable that though **2**, **3,** and **4** crystallize in the same space group of *C*222_1_ and feature the CH mode, the packing diagrams of **4** exhibits a different fashion as compared to those of **2** and **3** (Figure 4b–d, and Supplementary Materials, Figure S25).

From Table 1, it is notable that **1**, **5**, and **6** share nearly identical cell parameters and their molecular arrangement within the cells are also expected to resemble each other. Though with different β-CD-to-adamantane ratios, **1** and **5** share similar structural features in that all the adamantane derivatives are deeply buried inside of the cavity formed by β-CD (Figure 3a,d), and their structure packings (CH type) also resemble each other (Figure 4a,e). Compared to **1**, the β-CD pair in **5** is only able to host one adm-1,3-diCOOH molecule due to the bulky size of the latter, the disordered adm-1,3-diCOOH guest nevertheless exhibits preferential back-to-back orientations in a nearly 1:1 ratio (0.49:0.51). 

## 4. Conclusions

The guest inclusion behaviors of β-CD toward a series of simple adamantane derivatives carrying –OH, –NH_2_, –COOH, and –CH_2_COOH functionalities have been scrutinized. Our work suggests that the identity of functional groups (adm-1-OH in **1** versus adm-1-NH_2_ in **3**), geometry (adm-1-OH in **1** versus adm-2-OH in **2**), and size (adm-1,3-diCOOH in **5** and adm-1,3-diCH_2_COOH in **6**) collectively define the adamantane-to-β-CD ratios and the spatial locations of these guest molecules. Though these limited structural studies are far away from serving as an accurate predictor for the structural outcome in other scenarios, the structural features described herein may offer us some insight into the design of cyclodextrin-based host–guest assemblies; for example, to achieve the regioselectivity in the post-synthetic functionalization of the encapsulated adamantane derivatives, via the stealth of one functionality, while exposing the other for further modification. Given the broad and continuous interest in using β-CD and adamantane hybrid for designing efficient drug delivery systems for biomedical applications, and that –OH, –NH_2_, and –COOH are ideal functionalities to perform bio-conjugate chemistry under mild conditions, our work herein may provide insight and cautions for those working on supramolecular delivery systems utilizing cyclodextrin and adamantane hybrids.

## Figures and Tables

**Table 1:** Crystal data and refinement parameters for the **1–6.**

	1	2	3	4	5	6
Formula Weight	2574.41	2726.69	2723.73	2630.47	2494.20	2522.28
Empirical formula	2C_42_H_70_O_35_∙ 2C_10_H_16_O	2C_42_H_70_O_35_∙ 3C_10_H_16_O	2C_42_H_70_O_35_∙3C_10_ H_17_N	2C_42_H_70_O_35_∙ 2C_11_H_16_O_2_	2C_42_H_70_O_35_∙ C_12_H_16_O_4_	2C_42_H_70_O_35_∙ C_14_H_20_O_4_
Crystal system	triclinic	orthorhombic	orthorhombic	orthorhombic	triclinic	triclinic
Space group	*P*1	*C*222_1_	*C*222_1_	*C*222_1_	*P*1	*P*1
a (Å)	15.3986(9)	23.8095(5)	19.2469(5)	19.1225(4)	15.2993(8)	15.2868(3)
b (Å)	15.4175(8)	19.2806(4)	23.9691(5)	24.2639(6)	15.4429(7)	15.4435(3)
c (Å)	17.9599(10)	32.4542(9)	32.5978(9)	32.6433(8)	18.0837(10)	18.0918(4)
α (°)	113.169(2)	90	90	90.00	99.751(4)	100.0500(10)
β (°)	99.213(2)	90	90	90.00	113.551(5)	113.0330(10)
γ (°)	103.219(2)	90	90	90.00	102.813(4)	102.4900(10)
V (Å^3^)	3664.1(4)	14,898.5(6)	15,038.4(7)	15,146.0(6)	3656.6(4)	3674.56(13)
Z	1	1	4	4	1	1
ρ_calc_ (g cm^–3^)	1.167	1.097	1.031	1.075	1.133	1.026
*F*(000)	1372	5235	4754	5212	1324	1204
µ (cm^–1^)	0.545	0.520	0.802	0.093	0.099	0.504
Total reflections	50,836	96,200	72,586	55,633	27,091	46,007
Unique reflections	26,218	14,176	13,298	13,052	21,115	27,008
Observed reflections	19,199	11,540	12,601	9831	16,593	25,912
R_int_	0.0715	0.0782	0.0408	0.0361	0.0387	0.0400
Variables	1568	774	773	739	1676	1387
R_1_^a^	0.1024	0.082400853	0.0801	0.0883	0.0758	0.0648
*w*R_2_^b^	0.2643	0.2371	0.2303	0.2630	0.2080	0.1891
GOF ^c^	1.027	1.071	1.051	1.153	1.027	1.028

^a^ R_1_ = Σ||Fo|− - |Fc||/Σ|Fo|, ^b^ *w*R_2_ = {Σ[ω(F_o_^2^ - F_c_^2^)^2^]/Σ[ω(F_o_^2^)2]}^1/2^, and ^c^ GOF = {Σ[ω(F_o_^2^ - F_c_^2^)^2^]/(*n* - *p*)}^1/2^, where *n* is the number of reflections, and *p* the total number of parameters refined.

**Table 2:** A list of the packing modes of **1**–**6.**

	Guest	Guest:β-CD	Space Group	Packing Mode
**1**	adm-1-OH	2:2	*P*1	CH
**2**	adm-2-OH	3:2	*C*222_1_	CB
**3**	adm-1-NH_2_	3:2	*C*222_1_	CB
**4**	adm-1-COOH	2:2	*C*222_1_	CB
**5**	adm-1,3-diCOOH	1:2	*P*1	CH

## Data Availability

CCDC 2053567–2053572 contain the supplementary crystallographic data for this paper. These data can be obtained free of charge via www.ccdc.cam.ac.uk/data_request/cif, or by emailing data_request@ccdc.cam.ac.uk, or by contacting The Cambridge Crystallographic Data Centre, 12 Union Road, Cambridge CB2 1EZ, UK; fax: +44 1223 336033.

## Supplementary Materials

The following are available online: Figures S1–S12: The 1H-NMR spectra for **1**–**6**; Figures S13–S18: 2D NOESY spectra for **1**–**6**; Figures S19–S14: TG traces and DSC curves for **2**–**6**. Figure S24: The X-ray crystal structure of **3**. Figure S25: The crystal packing diagram of **6**; Tables S1–S12: The summarized data and remark of chemical shifts of β-cyclodextrin or adamantane derivatives before/after forming complexes **1**–**6**.
