# Supplementary material for: Structural Insights into the Host–Guest Complexation between β-Cyclodextrin and Bio-Conjugatable Adamantane Derivatives
Source: Molecules. 2021 Apr 21;26(9):2412. doi: 10.3390/molecules26092412 (PMC8122645; doi:10.3390/molecules26092412)

checkCIF/PLATON report

You have not supplied any structure factors. As a result the full set of tests cannot be run.

THIS REPORT IS FOR GUIDANCE ONLY. IF USED AS PART OF A REVIEW PROCEDURE FOR PUBLICATION, IT SHOULD NOT REPLACE THE EXPERTISE OF AN EXPERIENCED CRYSTALLOGRAPHIC REFEREE.

No syntax errors found. CIF dictionary Interpreting this report

Datablock: I

```
Bond precision:  C-C = 0.0092 A          Wavelength=1.34180

Cell:            a=23.8095(5)     b=19.2806(4)     c=32.4542(9)
                 alpha=90         beta=90          gamma=90
Temperature:     173 K
```

	Calculated	Reported
Volume	14898.5(6)	14898.5(6)
Space group	C 2 2 21	C 2 2 21
Hall group	C 2c 2	C 2c 2
Moiety formula	8(C42 H70 O35), 4(C10 H16 O), C10 H15 O [+ solvent]	?
Sum formula	C386 H639 O285 [+ solvent]	C386 H639 O285
Mr	9840.01	9839.93
Dx,g cm-3	1.097	1.097
Z	1	1
Mu (mm-1)	0.520	0.520
F000	5235.0	5235.0
F000’	5250.87	
h,k,lmax	29,23,39	29,23,39
Nref	14191[7630]	14176
Tmin,Tmax	0.928,0.959	0.622,0.751
Tmin’	0.925	

```
Correction method= # Reported T Limits: Tmin=0.622 Tmax=0.751
AbsCorr = MULTI-SCAN

Data completeness= 1.86/1.00      Theta(max)= 54.966

R(reflections)= 0.0824( 11540)    wR2(reflections)= 0.2371( 14176)

S = 1.071                  Npar= 774
```

The following ALERTS were generated. Each ALERT has the format
test-name_ALERT_alert-type_alert-level.
Click on the hyperlinks for more details of the test.

Alert level A

```
PLAT097_ALERT_2_A Large Reported Max.  (Positive) Residual Density       1.85 eA-3
```

Alert level B

```
PLAT250_ALERT_2_B Large U3/U1 Ratio for Average U(i,j) Tensor ....        6.1 Note
PLAT416_ALERT_2_B Short Intra D-H..H-D       H19      ..H23      .       1.64 Ang.
                                                      x,y,z  =      1_555 Check
PLAT417_ALERT_2_B Short Inter D-H..H-D       H23      ..H29      .       2.07 Ang.
                                                x,-1-y,-2-z  =      3_543 Check
PLAT417_ALERT_2_B Short Inter D-H..H-D       H24      ..H24      .       2.01 Ang.
                                                x,-1-y,-2-z  =      3_543 Check
PLAT420_ALERT_2_B D-H Bond Without Acceptor  O6       --H6       .     Please Check
PLAT420_ALERT_2_B D-H Bond Without Acceptor  O11      --H11      .     Please Check
PLAT420_ALERT_2_B D-H Bond Without Acceptor  O16      --H16      .     Please Check
PLAT420_ALERT_2_B D-H Bond Without Acceptor  O21      --H21      .     Please Check
PLAT420_ALERT_2_B D-H Bond Without Acceptor  O31      --H31      .     Please Check
```

Alert level C

```
DIFMX02_ALERT_1_C  The maximum difference density is > 0.1*ZMAX*0.75
            The relevant atom site should be identified.
RADNW01_ALERT_1_C  The radiation wavelength lies outside the expected range
            for the supplied radiation type. Expected range 1.34130-1.34150
            Wavelength given =    1.34180
PLAT048_ALERT_1_C MoietyFormula Not Given (or Incomplete) ........     Please Check
PLAT094_ALERT_2_C Ratio of Maximum / Minimum Residual Density ....       3.81 Report
PLAT220_ALERT_2_C NonSolvent   Resd 1  O   Ueq(max)/Ueq(min) Range        3.4 Ratio
PLAT222_ALERT_3_C NonSolvent Resd 1  H   Uiso(max)/Uiso(min) Range        4.5 Ratio
PLAT234_ALERT_4_C Large Hirshfeld Difference O31      --C37      .       0.17 Ang.
PLAT250_ALERT_2_C Large U3/U1 Ratio for Average U(i,j) Tensor ....        3.2 Note
PLAT260_ALERT_2_C Large Average Ueq of Residue Including       O37      0.130 Check
PLAT340_ALERT_3_C Low Bond Precision on  C-C Bonds ...............     0.0092 Ang.
PLAT411_ALERT_2_C Short Inter H...H Contact  H25A     ..H25A     .       2.13 Ang.
                                              -2-x,y,-5/2-z  =      4_352 Check
PLAT414_ALERT_2_C Short Intra D-H..H-X       H6       ..H9A              1.98 Ang.
                                                      x,y,z  =      1_555 Check
PLAT415_ALERT_2_C Short Inter D-H..H-X       H1A      ..H16      .       2.12 Ang.
                                       -5/2-x,-1/2+y,-5/2-z  =      8_242 Check
PLAT416_ALERT_2_C Short Intra D-H..H-D       H29      ..H33      .       1.97 Ang.
                                                      x,y,z  =      1_555 Check
PLAT417_ALERT_2_C Short Inter D-H..H-D       H4       ..H13      .       2.11 Ang.
                                                x,-1-y,-2-z  =      3_543 Check
PLAT417_ALERT_2_C Short Inter D-H..H-D       H24      ..H28      .       2.14 Ang.
                                                x,-1-y,-2-z  =      3_543 Check
PLAT790_ALERT_4_C Centre of Gravity not Within Unit Cell: Resd.  #          1 Note
              C42 H70 O35
```

Alert level G

```
ABSMU01_ALERT_1_G  Calculation of _exptl_absorpt_correction_mu
                not performed for this radiation type.
PLAT002_ALERT_2_G Number of Distance or Angle Restraints on AtSite         22 Note
PLAT007_ALERT_5_G Number of Unrefined Donor-H Atoms ..............         23 Report
PLAT032_ALERT_4_G Std. Uncertainty on Flack Parameter Value High .      0.300 Report
PLAT083_ALERT_2_G SHELXL Second Parameter in WGHT  Unusually Large      24.42 Why ?
```

```
PLAT171_ALERT_4_G The CIF-Embedded .res File Contains EADP Records          2 Report
PLAT175_ALERT_4_G The CIF-Embedded .res File Contains SAME Records          1 Report
PLAT300_ALERT_4_G Atom Site Occupancy of O36       Constrained at        0.5 Check
PLAT300_ALERT_4_G Atom Site Occupancy of C43       Constrained at        0.5 Check
PLAT300_ALERT_4_G Atom Site Occupancy of C44       Constrained at        0.5 Check
PLAT300_ALERT_4_G Atom Site Occupancy of C45       Constrained at        0.5 Check
PLAT300_ALERT_4_G Atom Site Occupancy of C46       Constrained at        0.5 Check
PLAT300_ALERT_4_G Atom Site Occupancy of C47       Constrained at        0.5 Check
PLAT300_ALERT_4_G Atom Site Occupancy of C48       Constrained at        0.5 Check
PLAT300_ALERT_4_G Atom Site Occupancy of C49       Constrained at        0.5 Check
PLAT300_ALERT_4_G Atom Site Occupancy of C50       Constrained at        0.5 Check
PLAT300_ALERT_4_G Atom Site Occupancy of C51       Constrained at        0.5 Check
PLAT300_ALERT_4_G Atom Site Occupancy of C52       Constrained at        0.5 Check
PLAT300_ALERT_4_G Atom Site Occupancy of H36       Constrained at        0.5 Check
PLAT300_ALERT_4_G Atom Site Occupancy of H43A      Constrained at        0.5 Check
PLAT300_ALERT_4_G Atom Site Occupancy of H44A      Constrained at        0.5 Check
PLAT300_ALERT_4_G Atom Site Occupancy of H44B      Constrained at        0.5 Check
PLAT300_ALERT_4_G Atom Site Occupancy of H45A      Constrained at        0.5 Check
PLAT300_ALERT_4_G Atom Site Occupancy of H45B      Constrained at        0.5 Check
PLAT300_ALERT_4_G Atom Site Occupancy of H46A      Constrained at        0.5 Check
PLAT300_ALERT_4_G Atom Site Occupancy of H46B      Constrained at        0.5 Check
PLAT300_ALERT_4_G Atom Site Occupancy of H47A      Constrained at        0.5 Check
PLAT300_ALERT_4_G Atom Site Occupancy of H48A      Constrained at        0.5 Check
PLAT300_ALERT_4_G Atom Site Occupancy of H49A      Constrained at        0.5 Check
PLAT300_ALERT_4_G Atom Site Occupancy of H49B      Constrained at        0.5 Check
PLAT300_ALERT_4_G Atom Site Occupancy of H50A      Constrained at        0.5 Check
PLAT300_ALERT_4_G Atom Site Occupancy of H51A      Constrained at        0.5 Check
PLAT300_ALERT_4_G Atom Site Occupancy of H51B      Constrained at        0.5 Check
PLAT300_ALERT_4_G Atom Site Occupancy of H52A      Constrained at        0.5 Check
PLAT300_ALERT_4_G Atom Site Occupancy of O37       Constrained at      0.125 Check
PLAT300_ALERT_4_G Atom Site Occupancy of C53       Constrained at      0.125 Check
PLAT300_ALERT_4_G Atom Site Occupancy of C54       Constrained at      0.125 Check
PLAT300_ALERT_4_G Atom Site Occupancy of C55       Constrained at      0.125 Check
PLAT300_ALERT_4_G Atom Site Occupancy of C56       Constrained at      0.125 Check
PLAT300_ALERT_4_G Atom Site Occupancy of C57       Constrained at      0.125 Check
PLAT300_ALERT_4_G Atom Site Occupancy of C58       Constrained at      0.125 Check
PLAT300_ALERT_4_G Atom Site Occupancy of C59       Constrained at      0.125 Check
PLAT300_ALERT_4_G Atom Site Occupancy of C60       Constrained at      0.125 Check
PLAT300_ALERT_4_G Atom Site Occupancy of C61       Constrained at      0.125 Check
PLAT300_ALERT_4_G Atom Site Occupancy of C62       Constrained at      0.125 Check
PLAT300_ALERT_4_G Atom Site Occupancy of H37       Constrained at      0.125 Check
PLAT300_ALERT_4_G Atom Site Occupancy of H53A      Constrained at      0.125 Check
PLAT300_ALERT_4_G Atom Site Occupancy of H54A      Constrained at      0.125 Check
PLAT300_ALERT_4_G Atom Site Occupancy of H54B      Constrained at      0.125 Check
PLAT300_ALERT_4_G Atom Site Occupancy of H55A      Constrained at      0.125 Check
PLAT300_ALERT_4_G Atom Site Occupancy of H55B      Constrained at      0.125 Check
PLAT300_ALERT_4_G Atom Site Occupancy of H56A      Constrained at      0.125 Check
PLAT300_ALERT_4_G Atom Site Occupancy of H56B      Constrained at      0.125 Check
PLAT300_ALERT_4_G Atom Site Occupancy of H58A      Constrained at      0.125 Check
PLAT300_ALERT_4_G Atom Site Occupancy of H59A      Constrained at      0.125 Check
PLAT300_ALERT_4_G Atom Site Occupancy of H59B      Constrained at      0.125 Check
PLAT300_ALERT_4_G Atom Site Occupancy of H60A      Constrained at      0.125 Check
PLAT300_ALERT_4_G Atom Site Occupancy of H61A      Constrained at      0.125 Check
PLAT300_ALERT_4_G Atom Site Occupancy of H61B      Constrained at      0.125 Check
PLAT300_ALERT_4_G Atom Site Occupancy of H62A      Constrained at      0.125 Check
PLAT302_ALERT_4_G Anion/Solvent/Minor-Residue Disorder (Resd  2  )       100% Note
PLAT302_ALERT_4_G Anion/Solvent/Minor-Residue Disorder (Resd  3  )       100% Note
PLAT304_ALERT_4_G Non-Integer Number of Atoms in ..... (Resd  2  )      13.50 Check
PLAT304_ALERT_4_G Non-Integer Number of Atoms in ..... (Resd  3  )       3.25 Check
PLAT417_ALERT_2_G Short Inter D-H..H-D       H26      ..H36      .       1.25 Ang.
                                              -2-x,y,-5/2-z  =      4_352 Check
PLAT606_ALERT_4_G Solvent Accessible VOID(S) in Structure ........          ! Info
```

```
PLAT721_ALERT_1_G Bond    Calc     0.85000, Rep     0.84000 Dev...       0.01 Ang.
              O37      -H37             1.555   1.555 ........     #  185 Check
PLAT722_ALERT_1_G Angle   Calc      110.00, Rep      111.10 Dev...       1.10 Degree
              C53     -C54     -H54A       1.555  1.555  1.555     #  357 Check
PLAT722_ALERT_1_G Angle   Calc      108.00, Rep      109.10 Dev...       1.10 Degree
              C54     -C60     -H60A       1.555  1.555  1.555     #  394 Check
PLAT789_ALERT_4_G Atoms with Negative _atom_site_disorder_group  #         26 Check
PLAT790_ALERT_4_G Centre of Gravity not Within Unit Cell: Resd.  #          2 Note
              C10 H16 O
PLAT790_ALERT_4_G Centre of Gravity not Within Unit Cell: Resd.  #          3 Note
              C10 H15 O
PLAT791_ALERT_4_G Model has Chirality at C2         (Sohnke SpGr)          R Verify
PLAT791_ALERT_4_G Model has Chirality at C3         (Sohnke SpGr)          S Verify
PLAT791_ALERT_4_G Model has Chirality at C4         (Sohnke SpGr)          R Verify
PLAT791_ALERT_4_G Model has Chirality at C5         (Sohnke SpGr)          R Verify
PLAT791_ALERT_4_G Model has Chirality at C6         (Sohnke SpGr)          R Verify
PLAT791_ALERT_4_G Model has Chirality at C8         (Sohnke SpGr)          R Verify
PLAT791_ALERT_4_G Model has Chirality at C9         (Sohnke SpGr)          S Verify
PLAT791_ALERT_4_G Model has Chirality at C10        (Sohnke SpGr)          R Verify
PLAT791_ALERT_4_G Model has Chirality at C11        (Sohnke SpGr)          R Verify
PLAT791_ALERT_4_G Model has Chirality at C12        (Sohnke SpGr)          R Verify
PLAT791_ALERT_4_G Model has Chirality at C14        (Sohnke SpGr)          R Verify
PLAT791_ALERT_4_G Model has Chirality at C15        (Sohnke SpGr)          S Verify
PLAT791_ALERT_4_G Model has Chirality at C16        (Sohnke SpGr)          R Verify
PLAT791_ALERT_4_G Model has Chirality at C17        (Sohnke SpGr)          R Verify
PLAT791_ALERT_4_G Model has Chirality at C18        (Sohnke SpGr)          R Verify
PLAT791_ALERT_4_G Model has Chirality at C20        (Sohnke SpGr)          R Verify
PLAT791_ALERT_4_G Model has Chirality at C21        (Sohnke SpGr)          S Verify
PLAT791_ALERT_4_G Model has Chirality at C22        (Sohnke SpGr)          R Verify
PLAT791_ALERT_4_G Model has Chirality at C23        (Sohnke SpGr)          R Verify
PLAT791_ALERT_4_G Model has Chirality at C24        (Sohnke SpGr)          R Verify
PLAT791_ALERT_4_G Model has Chirality at C26        (Sohnke SpGr)          R Verify
PLAT791_ALERT_4_G Model has Chirality at C27        (Sohnke SpGr)          S Verify
PLAT791_ALERT_4_G Model has Chirality at C28        (Sohnke SpGr)          R Verify
PLAT791_ALERT_4_G Model has Chirality at C29        (Sohnke SpGr)          R Verify
PLAT791_ALERT_4_G Model has Chirality at C30        (Sohnke SpGr)          R Verify
PLAT791_ALERT_4_G Model has Chirality at C32        (Sohnke SpGr)          R Verify
PLAT791_ALERT_4_G Model has Chirality at C33        (Sohnke SpGr)          S Verify
PLAT791_ALERT_4_G Model has Chirality at C34        (Sohnke SpGr)          R Verify
PLAT791_ALERT_4_G Model has Chirality at C35        (Sohnke SpGr)          R Verify
PLAT791_ALERT_4_G Model has Chirality at C36        (Sohnke SpGr)          R Verify
PLAT791_ALERT_4_G Model has Chirality at C38        (Sohnke SpGr)          R Verify
PLAT791_ALERT_4_G Model has Chirality at C39        (Sohnke SpGr)          S Verify
PLAT791_ALERT_4_G Model has Chirality at C40        (Sohnke SpGr)          R Verify
PLAT791_ALERT_4_G Model has Chirality at C41        (Sohnke SpGr)          R Verify
PLAT791_ALERT_4_G Model has Chirality at C42        (Sohnke SpGr)          R Verify
PLAT860_ALERT_3_G Number of Least-Squares Restraints .............        33 Note
PLAT869_ALERT_4_G ALERTS Related to the Use of SQUEEZE Suppressed          ! Info
PLAT883_ALERT_1_G No Info/Value for _atom_sites_solution_primary .     Please Do !
PLAT965_ALERT_2_G The SHELXL WEIGHT Optimisation has not Converged     Please Check
```

---

1 **ALERT level A** = Most likely a serious problem - resolve or explain
9 **ALERT level B** = A potentially serious problem, consider carefully
17 **ALERT level C** = Check. Ensure it is not caused by an omission or oversight
111 **ALERT level G** = General information/check it is not something unexpected

8 ALERT type 1 CIF construction/syntax error, inconsistent or missing data
24 ALERT type 2 Indicator that the structure model may be wrong or deficient
3 ALERT type 3 Indicator that the structure quality may be low
102 ALERT type 4 Improvement, methodology, query or suggestion
1 ALERT type 5 Informative message, check

checkCIF publication errors

Alert level A

```
PUBL004_ALERT_1_A  The contact author's name and address are missing,
            _publ_contact_author_name and _publ_contact_author_address.
PUBL005_ALERT_1_A  _publ_contact_author_email, _publ_contact_author_fax and
            _publ_contact_author_phone are all missing.
            At least one of these should be present.
PUBL006_ALERT_1_A  _publ_requested_journal is missing
            e.g. 'Acta Crystallographica Section C'
PUBL008_ALERT_1_A  _publ_section_title is missing. Title of paper.
PUBL009_ALERT_1_A  _publ_author_name is missing. List of author(s) name(s).
PUBL010_ALERT_1_A  _publ_author_address is missing. Author(s) address(es).
PUBL012_ALERT_1_A  _publ_section_abstract is missing.
            Abstract of paper in English.
```

Alert level G

```
PUBL017_ALERT_1_G The _publ_section_references section is missing or
            empty.
```

```
   7 ALERT level A = Data missing that is essential or data in wrong format
   1 ALERT level G = General alerts. Data that may be required is missing
```

Publication of your CIF

You should attempt to resolve as many as possible of the alerts in all categories. Often the minor alerts point to easily fixed oversights, errors and omissions in your CIF or refinement strategy, so attention to these fine details can be worthwhile. In order to resolve some of the more serious problems it may be necessary to carry out additional measurements or structure refinements. However, the nature of your study may justify the reported deviations from journal submission requirements and the more serious of these should be commented upon in the discussion or experimental section of a paper or in the "special_details" fields of the CIF. *checkCIF* was carefully designed to identify outliers and unusual parameters, but every test has its limitations and alerts that are not important in a particular case may appear. Conversely, the absence of alerts does not guarantee there are no aspects of the results needing attention. It is up to the individual to critically assess their own results and, if necessary, seek expert advice.

If level A alerts remain, which you believe to be justified deviations, and you intend to submit this CIF for publication in a journal, you should additionally insert an explanation in your CIF using the Validation Reply Form (VRF) below. This will allow your explanation to be considered as part of the review process.

Validation response form

Please find below a validation response form (VRF) that can be filled in and pasted into your CIF.

```
# start Validation Reply Form
_vrf_PUBL004_GLOBAL
;
PROBLEM: The contact author’s name and address are missing,
RESPONSE: ...
;
_vrf_PUBL005_GLOBAL
;
PROBLEM: _publ_contact_author_email, _publ_contact_author_fax and
RESPONSE: ...
;
_vrf_PUBL006_GLOBAL
;
PROBLEM: _publ_requested_journal is missing
RESPONSE: ...
;
_vrf_PUBL008_GLOBAL
;
PROBLEM: _publ_section_title is missing. Title of paper.
RESPONSE: ...
;
_vrf_PUBL009_GLOBAL
;
PROBLEM: _publ_author_name is missing. List of author(s) name(s).
RESPONSE: ...
;
_vrf_PUBL010_GLOBAL
;
PROBLEM: _publ_author_address is missing. Author(s) address(es).
RESPONSE: ...
;
_vrf_PUBL012_GLOBAL
;
PROBLEM: _publ_section_abstract is missing.
RESPONSE: ...
;
_vrf_PLAT097_I
;
PROBLEM: Large Reported Max.  (Positive) Residual Density       1.85 eA-3
RESPONSE: ...
;
# end Validation Reply Form
```

If you wish to submit your CIF for publication in Acta Crystallographica Section C or E, you should upload your CIF via the web. If you wish to submit your CIF for publication in IUCrData you should upload your CIF via the web. If your CIF is to form part of a submission to another IUCr journal, you will be asked, either during electronic submission or by the Co-editor handling your paper, to upload your CIF via our web site.

PLATON version of 22/03/2021; check.def file version of 19/03/2021

Datablock I - ellipsoid plot

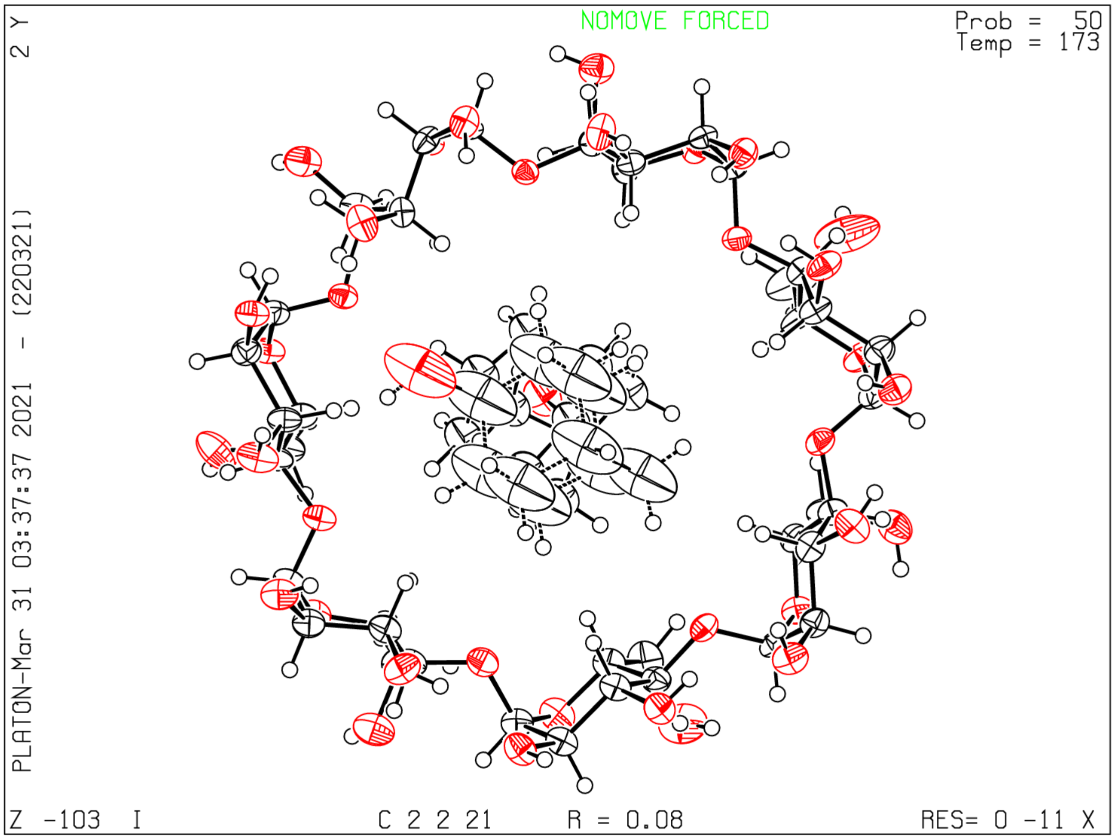

Supplement: Supplementary file 1 [file molecules-26-02412-s001.zip › CIF and CheckCIF/CheckCIF_Complex 2.pdf]
