# Supplementary material for: Structural Insights into the Host–Guest Complexation between β-Cyclodextrin and Bio-Conjugatable Adamantane Derivatives
Source: Molecules. 2021 Apr 21;26(9):2412. doi: 10.3390/molecules26092412 (PMC8122645; doi:10.3390/molecules26092412)

checkCIF/PLATON report

You have not supplied any structure factors. As a result the full set of tests cannot be run.

THIS REPORT IS FOR GUIDANCE ONLY. IF USED AS PART OF A REVIEW PROCEDURE FOR PUBLICATION, IT SHOULD NOT REPLACE THE EXPERTISE OF AN EXPERIENCED CRYSTALLOGRAPHIC REFEREE.

No syntax errors found. CIF dictionary Interpreting this report

Datablock: I

```
Bond precision:  C-C = 0.0075 A          Wavelength=1.54184

Cell:            a=19.2469(5)     b=23.9691(5)     c=32.5978(9)
                 alpha=90         beta=90          gamma=90
Temperature:     173 K
```

	Calculated	Reported
Volume	15038.4(7)	15038.4(7)
Space group	C 2 2 21	C 2 2 21
Hall group	C 2c 2	C 2c 2
Moiety formula	2(C42 H21 O35), C10 H2 N, 0.2(C10 N) [+ solvent]	?
Sum formula	C96 H44 N1.20 O70 [+ solvent]	C96 H44 N1.20 O70
Mr	2334.13	2334.12
Dx,g cm-3	1.031	1.031
Z	4	4
Mu (mm-1)	0.802	0.802
F000	4753.6	4754.0
F000’	4773.98	
h,k,lmax	22,28,38	22,28,38
Nref	13325[7176]	13298
Tmin,Tmax	0.908,0.938	0.222,0.318
Tmin’	0.908	

```
Correction method= # Reported T Limits: Tmin=0.222 Tmax=0.318
AbsCorr = MULTI-SCAN

Data completeness= 1.85/1.00      Theta(max)= 66.708

R(reflections)= 0.0802( 12601)    wR2(reflections)= 0.2320( 13298)

S = 2.014                  Npar= 773
```

The following ALERTS were generated. Each ALERT has the format
test-name_ALERT_alert-type_alert-level.
Click on the hyperlinks for more details of the test.

Alert level B

```
PLAT097_ALERT_2_B Large Reported Max.  (Positive) Residual Density       1.30 eA-3
PLAT250_ALERT_2_B Large U3/U1 Ratio for Average U(i,j) Tensor ....        4.4 Note
PLAT416_ALERT_2_B Short Intra D-H..H-D       H8        ..H14      .       1.65 Ang.
                                                    x,y,z  =      1_555 Check
PLAT417_ALERT_2_B Short Inter D-H..H-D       H3        ..H9       .       2.09 Ang.
                                              1-x,y,3/2-z  =      4_656 Check
PLAT417_ALERT_2_B Short Inter D-H..H-D       H6        ..H26      .       1.89 Ang.
                                          -1/2+x,-1/2+y,z  =      5_445 Check
PLAT417_ALERT_2_B Short Inter D-H..H-D       H9        ..H9       .       2.02 Ang.
                                              1-x,y,3/2-z  =      4_656 Check
PLAT417_ALERT_2_B Short Inter D-H..H-D       H19       ..H28      .       2.04 Ang.
                                              1-x,y,3/2-z  =      4_656 Check
PLAT420_ALERT_2_B D-H Bond Without Acceptor  O1        --H1       .     Please Check
PLAT420_ALERT_2_B D-H Bond Without Acceptor  O6        --H6       .     Please Check
PLAT420_ALERT_2_B D-H Bond Without Acceptor  O16       --H16      .     Please Check
PLAT420_ALERT_2_B D-H Bond Without Acceptor  O21       --H21      .     Please Check
PLAT420_ALERT_2_B D-H Bond Without Acceptor  O33       --H33      .     Please Check
```

Alert level C

```
ABSTY02_ALERT_1_C  An _exptl_absorpt_correction_type has been given without
            a literature citation. This should be contained in the
            _exptl_absorpt_process_details field.
            Absorption correction given as multi-scan
DIFMX02_ALERT_1_C  The maximum difference density is > 0.1*ZMAX*0.75
            The relevant atom site should be identified.
GOODF01_ALERT_2_C  The least squares goodness of fit parameter lies
            outside the range 0.80 <> 2.00
            Goodness of fit given =      2.014
PLAT048_ALERT_1_C MoietyFormula Not Given (or Incomplete) ........     Please Check
PLAT077_ALERT_4_C Unitcell Contains Non-integer Number of Atoms ..     Please Check
PLAT085_ALERT_2_C SHELXL Default Weighting Scheme is not Optimized     Please Check
PLAT087_ALERT_2_C Unsatisfactory S value (Too High) .............       2.01 Check
PLAT094_ALERT_2_C Ratio of Maximum / Minimum Residual Density ....      2.25 Report
PLAT220_ALERT_2_C NonSolvent   Resd 1  O   Ueq(max)/Ueq(min) Range       3.6 Ratio
PLAT230_ALERT_2_C Hirshfeld Test Diff for    O31       --C37      .       5.1 s.u.
PLAT250_ALERT_2_C Large U3/U1 Ratio for Average U(i,j) Tensor ....        2.2 Note
PLAT340_ALERT_3_C Low Bond Precision on  C-C Bonds ..............    0.00754 Ang.
PLAT417_ALERT_2_C Short Inter D-H..H-D       H23       ..H24      .       2.10 Ang.
                                              1-x,y,3/2-z  =      4_656 Check
PLAT420_ALERT_2_C D-H Bond Without Acceptor  N1        --H1B      .     Please Check
```

Alert level G

```
PLAT002_ALERT_2_G Number of Distance or Angle Restraints on AtSite         22 Note
PLAT007_ALERT_5_G Number of Unrefined Donor-H Atoms ..............         23 Report
PLAT171_ALERT_4_G The CIF-Embedded .res File Contains EADP Records          2 Report
PLAT175_ALERT_4_G The CIF-Embedded .res File Contains SAME Records          1 Report
PLAT300_ALERT_4_G Atom Site Occupancy of N1         Constrained at        0.5 Check
PLAT300_ALERT_4_G Atom Site Occupancy of C43        Constrained at        0.5 Check
PLAT300_ALERT_4_G Atom Site Occupancy of C44        Constrained at        0.5 Check
PLAT300_ALERT_4_G Atom Site Occupancy of C45        Constrained at        0.5 Check
PLAT300_ALERT_4_G Atom Site Occupancy of C46        Constrained at        0.5 Check
PLAT300_ALERT_4_G Atom Site Occupancy of C47        Constrained at        0.5 Check
PLAT300_ALERT_4_G Atom Site Occupancy of C48        Constrained at        0.5 Check
```

```
PLAT300_ALERT_4_G Atom Site Occupancy of C49        Constrained at        0.5 Check
PLAT300_ALERT_4_G Atom Site Occupancy of C50        Constrained at        0.5 Check
PLAT300_ALERT_4_G Atom Site Occupancy of C51        Constrained at        0.5 Check
PLAT300_ALERT_4_G Atom Site Occupancy of C52        Constrained at        0.5 Check
PLAT300_ALERT_4_G Atom Site Occupancy of H1A        Constrained at        0.5 Check
PLAT300_ALERT_4_G Atom Site Occupancy of H1B        Constrained at        0.5 Check
PLAT300_ALERT_4_G Atom Site Occupancy of N2         Constrained at        0.1 Check
PLAT300_ALERT_4_G Atom Site Occupancy of C53        Constrained at        0.1 Check
PLAT300_ALERT_4_G Atom Site Occupancy of C54        Constrained at        0.1 Check
PLAT300_ALERT_4_G Atom Site Occupancy of C55        Constrained at        0.1 Check
PLAT300_ALERT_4_G Atom Site Occupancy of C56        Constrained at        0.1 Check
PLAT300_ALERT_4_G Atom Site Occupancy of C57        Constrained at        0.1 Check
PLAT300_ALERT_4_G Atom Site Occupancy of C58        Constrained at        0.1 Check
PLAT300_ALERT_4_G Atom Site Occupancy of C59        Constrained at        0.1 Check
PLAT300_ALERT_4_G Atom Site Occupancy of C60        Constrained at        0.1 Check
PLAT300_ALERT_4_G Atom Site Occupancy of C61        Constrained at        0.1 Check
PLAT300_ALERT_4_G Atom Site Occupancy of C62        Constrained at        0.1 Check
PLAT302_ALERT_4_G Anion/Solvent/Minor-Residue Disorder (Resd  2  )       100% Note
PLAT302_ALERT_4_G Anion/Solvent/Minor-Residue Disorder (Resd  3  )       100% Note
PLAT304_ALERT_4_G Non-Integer Number of Atoms in ..... (Resd  2  )       6.50 Check
PLAT304_ALERT_4_G Non-Integer Number of Atoms in ..... (Resd  3  )       1.10 Check
PLAT315_ALERT_2_G Singly Bonded Carbon Detected (H-atoms Missing).        C51 Check
PLAT315_ALERT_2_G Singly Bonded Carbon Detected (H-atoms Missing).        C61 Check
PLAT343_ALERT_2_G Unusual sp?      Angle Range in Main Residue for         C1 Check
PLAT343_ALERT_2_G Unusual sp?      Angle Range in Main Residue for         C2 Check
PLAT343_ALERT_2_G Unusual sp?      Angle Range in Main Residue for         C3 Check
PLAT343_ALERT_2_G Unusual sp?      Angle Range in Main Residue for         C4 Check
PLAT343_ALERT_2_G Unusual sp?      Angle Range in Main Residue for         C5 Check
PLAT343_ALERT_2_G Unusual sp?      Angle Range in Main Residue for         C6 Check
PLAT343_ALERT_2_G Unusual sp?      Angle Range in Main Residue for         C7 Check
PLAT343_ALERT_2_G Unusual sp?      Angle Range in Main Residue for         C8 Check
PLAT343_ALERT_2_G Unusual sp?      Angle Range in Main Residue for         C9 Check
PLAT343_ALERT_2_G Unusual sp?      Angle Range in Main Residue for        C10 Check
PLAT343_ALERT_2_G Unusual sp?      Angle Range in Main Residue for        C11 Check
PLAT343_ALERT_2_G Unusual sp?      Angle Range in Main Residue for        C12 Check
PLAT343_ALERT_2_G Unusual sp?      Angle Range in Main Residue for        C13 Check
PLAT343_ALERT_2_G Unusual sp?      Angle Range in Main Residue for        C14 Check
PLAT343_ALERT_2_G Unusual sp?      Angle Range in Main Residue for        C15 Check
PLAT343_ALERT_2_G Unusual sp?      Angle Range in Main Residue for        C16 Check
PLAT343_ALERT_2_G Unusual sp?      Angle Range in Main Residue for        C17 Check
PLAT343_ALERT_2_G Unusual sp?      Angle Range in Main Residue for        C18 Check
PLAT343_ALERT_2_G Unusual sp?      Angle Range in Main Residue for        C19 Check
PLAT343_ALERT_2_G Unusual sp?      Angle Range in Main Residue for        C20 Check
PLAT343_ALERT_2_G Unusual sp?      Angle Range in Main Residue for        C21 Check
PLAT343_ALERT_2_G Unusual sp?      Angle Range in Main Residue for        C22 Check
PLAT343_ALERT_2_G Unusual sp?      Angle Range in Main Residue for        C23 Check
PLAT343_ALERT_2_G Unusual sp?      Angle Range in Main Residue for        C24 Check
PLAT343_ALERT_2_G Unusual sp?      Angle Range in Main Residue for        C25 Check
PLAT343_ALERT_2_G Unusual sp?      Angle Range in Main Residue for        C26 Check
PLAT343_ALERT_2_G Unusual sp?      Angle Range in Main Residue for        C27 Check
PLAT343_ALERT_2_G Unusual sp?      Angle Range in Main Residue for        C28 Check
PLAT343_ALERT_2_G Unusual sp?      Angle Range in Main Residue for        C29 Check
PLAT343_ALERT_2_G Unusual sp?      Angle Range in Main Residue for        C30 Check
PLAT343_ALERT_2_G Unusual sp?      Angle Range in Main Residue for        C31 Check
PLAT343_ALERT_2_G Unusual sp?      Angle Range in Main Residue for        C32 Check
PLAT343_ALERT_2_G Unusual sp?      Angle Range in Main Residue for        C33 Check
PLAT343_ALERT_2_G Unusual sp?      Angle Range in Main Residue for        C34 Check
PLAT343_ALERT_2_G Unusual sp?      Angle Range in Main Residue for        C35 Check
PLAT343_ALERT_2_G Unusual sp?      Angle Range in Main Residue for        C36 Check
PLAT343_ALERT_2_G Unusual sp?      Angle Range in Main Residue for        C37 Check
PLAT343_ALERT_2_G Unusual sp?      Angle Range in Main Residue for        C38 Check
PLAT343_ALERT_2_G Unusual sp?      Angle Range in Main Residue for        C39 Check
```

```
PLAT343_ALERT_2_G Unusual sp?      Angle Range in Main Residue for        C40 Check
PLAT343_ALERT_2_G Unusual sp?      Angle Range in Main Residue for        C41 Check
PLAT343_ALERT_2_G Unusual sp?      Angle Range in Main Residue for        C42 Check
PLAT367_ALERT_2_G Long?  C(sp?)-C(sp?) Bond  C1       - C2       .       1.52 Ang.
PLAT367_ALERT_2_G Long?  C(sp?)-C(sp?) Bond  C2       - C3       .       1.53 Ang.
PLAT367_ALERT_2_G Long?  C(sp?)-C(sp?) Bond  C3       - C4       .       1.52 Ang.
PLAT367_ALERT_2_G Long?  C(sp?)-C(sp?) Bond  C4       - C5       .       1.53 Ang.
PLAT367_ALERT_2_G Long?  C(sp?)-C(sp?) Bond  C5       - C6       .       1.51 Ang.
PLAT367_ALERT_2_G Long?  C(sp?)-C(sp?) Bond  C7       - C8       .       1.50 Ang.
PLAT367_ALERT_2_G Long?  C(sp?)-C(sp?) Bond  C8       - C9       .       1.54 Ang.
PLAT367_ALERT_2_G Long?  C(sp?)-C(sp?) Bond  C9       - C10      .       1.51 Ang.
PLAT367_ALERT_2_G Long?  C(sp?)-C(sp?) Bond  C10      - C11      .       1.50 Ang.
PLAT367_ALERT_2_G Long?  C(sp?)-C(sp?) Bond  C11      - C12      .       1.53 Ang.
PLAT367_ALERT_2_G Long?  C(sp?)-C(sp?) Bond  C13      - C14      .       1.52 Ang.
PLAT367_ALERT_2_G Long?  C(sp?)-C(sp?) Bond  C14      - C15      .       1.53 Ang.
PLAT367_ALERT_2_G Long?  C(sp?)-C(sp?) Bond  C15      - C16      .       1.52 Ang.
PLAT367_ALERT_2_G Long?  C(sp?)-C(sp?) Bond  C16      - C17      .       1.52 Ang.
PLAT367_ALERT_2_G Long?  C(sp?)-C(sp?) Bond  C17      - C18      .       1.52 Ang.
PLAT367_ALERT_2_G Long?  C(sp?)-C(sp?) Bond  C19      - C20      .       1.51 Ang.
PLAT367_ALERT_2_G Long?  C(sp?)-C(sp?) Bond  C20      - C21      .       1.54 Ang.
PLAT367_ALERT_2_G Long?  C(sp?)-C(sp?) Bond  C21      - C22      .       1.51 Ang.
PLAT367_ALERT_2_G Long?  C(sp?)-C(sp?) Bond  C22      - C23      .       1.51 Ang.
PLAT367_ALERT_2_G Long?  C(sp?)-C(sp?) Bond  C23      - C24      .       1.53 Ang.
PLAT367_ALERT_2_G Long?  C(sp?)-C(sp?) Bond  C25      - C26      .       1.52 Ang.
PLAT367_ALERT_2_G Long?  C(sp?)-C(sp?) Bond  C26      - C27      .       1.52 Ang.
PLAT367_ALERT_2_G Long?  C(sp?)-C(sp?) Bond  C27      - C28      .       1.52 Ang.
PLAT367_ALERT_2_G Long?  C(sp?)-C(sp?) Bond  C28      - C29      .       1.53 Ang.
PLAT367_ALERT_2_G Long?  C(sp?)-C(sp?) Bond  C29      - C30      .       1.54 Ang.
PLAT367_ALERT_2_G Long?  C(sp?)-C(sp?) Bond  C31      - C32      .       1.53 Ang.
PLAT367_ALERT_2_G Long?  C(sp?)-C(sp?) Bond  C32      - C33      .       1.52 Ang.
PLAT367_ALERT_2_G Long?  C(sp?)-C(sp?) Bond  C33      - C34      .       1.52 Ang.
PLAT367_ALERT_2_G Long?  C(sp?)-C(sp?) Bond  C34      - C35      .       1.52 Ang.
PLAT367_ALERT_2_G Long?  C(sp?)-C(sp?) Bond  C35      - C36      .       1.52 Ang.
PLAT367_ALERT_2_G Long?  C(sp?)-C(sp?) Bond  C37      - C38      .       1.52 Ang.
PLAT367_ALERT_2_G Long?  C(sp?)-C(sp?) Bond  C38      - C39      .       1.55 Ang.
PLAT367_ALERT_2_G Long?  C(sp?)-C(sp?) Bond  C39      - C40      .       1.51 Ang.
PLAT367_ALERT_2_G Long?  C(sp?)-C(sp?) Bond  C40      - C41      .       1.51 Ang.
PLAT367_ALERT_2_G Long?  C(sp?)-C(sp?) Bond  C41      - C42      .       1.52 Ang.
PLAT606_ALERT_4_G Solvent Accessible VOID(S) in Structure ........          ! Info
PLAT773_ALERT_2_G Check long C-C Bond in CIF: C50      --C51             1.77 Ang.
PLAT773_ALERT_2_G Check long C-C Bond in CIF: C60      --C61             1.77 Ang.
PLAT789_ALERT_4_G Atoms with Negative _atom_site_disorder_group  #         11 Check
PLAT791_ALERT_4_G Model has Chirality at C43         (Sohnke SpGr)          R Verify
PLAT860_ALERT_3_G Number of Least-Squares Restraints .............         34 Note
PLAT869_ALERT_4_G ALERTS Related to the Use of SQUEEZE Suppressed           ! Info
PLAT883_ALERT_1_G No Info/Value for _atom_sites_solution_primary .     Please Do !
PLAT965_ALERT_2_G The SHELXL WEIGHT Optimisation has not Converged     Please Check
```

```
  0 ALERT level A = Most likely a serious problem - resolve or explain
 12 ALERT level B = A potentially serious problem, consider carefully
 14 ALERT level C = Check. Ensure it is not caused by an omission or oversight
120 ALERT level G = General information/check it is not something unexpected

  4 ALERT type 1 CIF construction/syntax error, inconsistent or missing data
104 ALERT type 2 Indicator that the structure model may be wrong or deficient
  2 ALERT type 3 Indicator that the structure quality may be low
 35 ALERT type 4 Improvement, methodology, query or suggestion
  1 ALERT type 5 Informative message, check
```

---

checkCIF publication errors

Alert level A

```
PUBL004_ALERT_1_A  The contact author's name and address are missing,
            _publ_contact_author_name and _publ_contact_author_address.
PUBL005_ALERT_1_A  _publ_contact_author_email, _publ_contact_author_fax and
            _publ_contact_author_phone are all missing.
            At least one of these should be present.
PUBL006_ALERT_1_A  _publ_requested_journal is missing
            e.g. 'Acta Crystallographica Section C'
PUBL008_ALERT_1_A  _publ_section_title is missing. Title of paper.
PUBL009_ALERT_1_A  _publ_author_name is missing. List of author(s) name(s).
PUBL010_ALERT_1_A  _publ_author_address is missing. Author(s) address(es).
PUBL012_ALERT_1_A  _publ_section_abstract is missing.
            Abstract of paper in English.
```

Alert level G

```
PUBL017_ALERT_1_G The _publ_section_references section is missing or
            empty.
```

---

7 **ALERT level A** = Data missing that is essential or data in wrong format
1 **ALERT level G** = General alerts. Data that may be required is missing

---

Publication of your CIF

You should attempt to resolve as many as possible of the alerts in all categories. Often the minor alerts point to easily fixed oversights, errors and omissions in your CIF or refinement strategy, so attention to these fine details can be worthwhile. In order to resolve some of the more serious problems it may be necessary to carry out additional measurements or structure refinements. However, the nature of your study may justify the reported deviations from journal submission requirements and the more serious of these should be commented upon in the discussion or experimental section of a paper or in the "special_details" fields of the CIF. *checkCIF* was carefully designed to identify outliers and unusual parameters, but every test has its limitations and alerts that are not important in a particular case may appear. Conversely, the absence of alerts does not guarantee there are no aspects of the results needing attention. It is up to the individual to critically assess their own results and, if necessary, seek expert advice.

If level A alerts remain, which you believe to be justified deviations, and you intend to submit this CIF for publication in a journal, you should additionally insert an explanation in your CIF using the Validation Reply Form (VRF) below. This will allow your explanation to be considered as part of the review process.

Validation response form

Please find below a validation response form (VRF) that can be filled in and pasted into your CIF.

```
# start Validation Reply Form
_vrf_PUBL004_GLOBAL
;
PROBLEM: The contact author's name and address are missing,
```

```
RESPONSE: ...
;
_vrf_PUBL005_GLOBAL
;
PROBLEM: _publ_contact_author_email, _publ_contact_author_fax and
RESPONSE: ...
;
_vrf_PUBL006_GLOBAL
;
PROBLEM: _publ_requested_journal is missing
RESPONSE: ...
;
_vrf_PUBL008_GLOBAL
;
PROBLEM: _publ_section_title is missing. Title of paper.
RESPONSE: ...
;
_vrf_PUBL009_GLOBAL
;
PROBLEM: _publ_author_name is missing. List of author(s) name(s).
RESPONSE: ...
;
_vrf_PUBL010_GLOBAL
;
PROBLEM: _publ_author_address is missing. Author(s) address(es).
RESPONSE: ...
;
_vrf_PUBL012_GLOBAL
;
PROBLEM: _publ_section_abstract is missing.
RESPONSE: ...
;
# end Validation Reply Form
```

If you wish to submit your CIF for publication in Acta Crystallographica Section C or E, you should upload your CIF via the web. If you wish to submit your CIF for publication in IUCrData you should upload your CIF via the web. If your CIF is to form part of a submission to another IUCr journal, you will be asked, either during electronic submission or by the Co-editor handling your paper, to upload your CIF via our web site.

PLATON version of 22/03/2021; check.def file version of 19/03/2021

Datablock I - ellipsoid plot

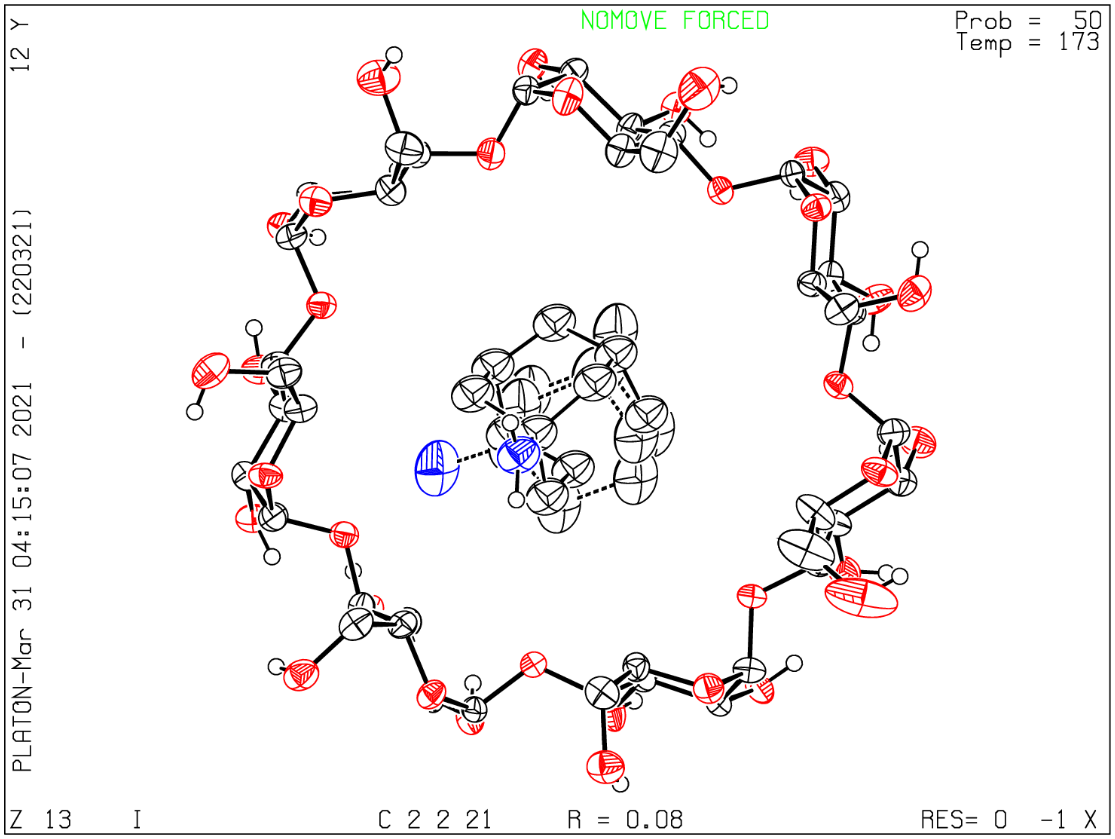

Supplement: Supplementary file 1 [file molecules-26-02412-s001.zip › CIF and CheckCIF/CheckCIF_Complex 3.pdf]
