# Supplementary material for: Structural Insights into the Host–Guest Complexation between β-Cyclodextrin and Bio-Conjugatable Adamantane Derivatives
Source: Molecules. 2021 Apr 21;26(9):2412. doi: 10.3390/molecules26092412 (PMC8122645; doi:10.3390/molecules26092412)

checkCIF/PLATON report

You have not supplied any structure factors. As a result the full set of tests cannot be run.

THIS REPORT IS FOR GUIDANCE ONLY. IF USED AS PART OF A REVIEW PROCEDURE FOR PUBLICATION, IT SHOULD NOT REPLACE THE EXPERTISE OF AN EXPERIENCED CRYSTALLOGRAPHIC REFEREE.

No syntax errors found. CIF dictionary Interpreting this report

Datablock: I

```
Bond precision:  C-C = 0.0098 A          Wavelength=0.71073

Cell:            a=19.1225(4)     b=24.2639(6)     c=32.6433(8)
                 alpha=90         beta=90          gamma=90
Temperature:     296 K
```

	Calculated	Reported
Volume	15146.1(6)	15146.0(6)
Space group	C 2 2 21	C 2 2 21
Hall group	C 2c 2	C 2c 2
Moiety formula	2(C42 H70 O35), 2(C5.50 H8.50 O) [+ solvent]	2(C42 H70 O35), 2(C5.50 H8.50 O) [+ SOLVENT]
Sum formula	C95 H157 O72 [+ solvent]	C95 H157 O72
Mr	2451.21	2451.20
Dx,g cm-3	1.075	1.075
Z	4	4
Mu (mm-1)	0.093	0.093
F000	5212.0	5212.0
F000’	5215.80	
h,k,lmax	22,28,38	22,28,38
Nref	13382[7205]	13052
Tmin,Tmax	0.970,0.974	0.970,0.974
Tmin’	0.970	

```
Correction method= # Reported T Limits: Tmin=0.970 Tmax=0.974
AbsCorr = MULTI-SCAN

Data completeness= 1.81/0.98      Theta(max)= 25.019

R(reflections)= 0.0883( 9831)     wR2(reflections)= 0.2859( 13052)

S = 1.153                   Npar= 739
```

The following ALERTS were generated. Each ALERT has the format
test-name_ALERT_alert-type_alert-level.
Click on the hyperlinks for more details of the test.

Alert level A

```
PLAT097_ALERT_2_A Large Reported Max.  (Positive) Residual Density       1.82 eA-3
```

Alert level B

```
PLAT230_ALERT_2_B Hirshfeld Test Diff for    O30      --C36      .       11.2 s.u.
PLAT416_ALERT_2_B Short Intra D-H..H-D       H3       ..H9       .       1.50 Ang.
                                                   x,y,z  =      1_555 Check
PLAT416_ALERT_2_B Short Intra D-H..H-D       H13      ..H19      .       1.47 Ang.
                                                   x,y,z  =      1_555 Check
PLAT417_ALERT_2_B Short Inter D-H..H-D       H8       ..H9       .       2.06 Ang.
                                             1-x,y,1/2-z  =      4_655 Check
PLAT417_ALERT_2_B Short Inter D-H..H-D       H15      ..H30      .       2.00 Ang.
                                             -1/2+x,1/2+y,z  =      5_455 Check
PLAT420_ALERT_2_B D-H Bond Without Acceptor  O5       --H5O      .     Please Check
PLAT420_ALERT_2_B D-H Bond Without Acceptor  O10      --H10      .     Please Check
PLAT420_ALERT_2_B D-H Bond Without Acceptor  O15      --H15      .     Please Check
PLAT420_ALERT_2_B D-H Bond Without Acceptor  O35      --H35      .     Please Check
PLAT420_ALERT_2_B D-H Bond Without Acceptor  O51      --H51O     .     Please Check
```

Alert level C

```
DIFMX02_ALERT_1_C  The maximum difference density is > 0.1*ZMAX*0.75
            The relevant atom site should be identified.
PLAT084_ALERT_3_C High wR2 Value (i.e. > 0.25) ...................       0.29 Report
PLAT094_ALERT_2_C Ratio of Maximum / Minimum Residual Density ....       3.56 Report
PLAT242_ALERT_2_C Low    'MainMol' Ueq as Compared to Neighbors of        C36 Check
PLAT260_ALERT_2_C Large Average Ueq of Residue Including       O51      0.109 Check
PLAT340_ALERT_3_C Low Bond Precision on  C-C Bonds ...............     0.0098 Ang.
PLAT417_ALERT_2_C Short Inter D-H..H-D       H24O     ..H28      .       2.12 Ang.
                                             1-x,y,1/2-z  =      4_655 Check
```

Alert level G

```
FORMU01_ALERT_1_G  There is a discrepancy between the atom counts in the
            _chemical_formula_sum and _chemical_formula_moiety. This is
            usually due to the moiety formula being in the wrong format.
            Atom count from _chemical_formula_sum:   C95 H157 O72
            Atom count from _chemical_formula_moiety:C84 H140 O70
PLAT002_ALERT_2_G Number of Distance or Angle Restraints on AtSite         11 Note
PLAT007_ALERT_5_G Number of Unrefined Donor-H Atoms ..............         22 Report
PLAT066_ALERT_1_G Predicted and Reported Tmin&Tmax Range Identical          ? Check
PLAT171_ALERT_4_G The CIF-Embedded .res File Contains EADP Records          1 Report
PLAT172_ALERT_4_G The CIF-Embedded .res File Contains DFIX Records          3 Report
PLAT173_ALERT_4_G The CIF-Embedded .res File Contains DANG Records          1 Report
PLAT300_ALERT_4_G Atom Site Occupancy of O51        Constrained at        0.5 Check
PLAT300_ALERT_4_G Atom Site Occupancy of O52        Constrained at        0.5 Check
PLAT300_ALERT_4_G Atom Site Occupancy of C51        Constrained at        0.5 Check
PLAT300_ALERT_4_G Atom Site Occupancy of C52        Constrained at        0.5 Check
PLAT300_ALERT_4_G Atom Site Occupancy of C53        Constrained at        0.5 Check
PLAT300_ALERT_4_G Atom Site Occupancy of C54        Constrained at        0.5 Check
PLAT300_ALERT_4_G Atom Site Occupancy of C55        Constrained at        0.5 Check
PLAT300_ALERT_4_G Atom Site Occupancy of C56        Constrained at        0.5 Check
PLAT300_ALERT_4_G Atom Site Occupancy of C57        Constrained at        0.5 Check
PLAT300_ALERT_4_G Atom Site Occupancy of C58        Constrained at        0.5 Check
PLAT300_ALERT_4_G Atom Site Occupancy of C59        Constrained at        0.5 Check
```

```
PLAT300_ALERT_4_G Atom Site Occupancy of C60        Constrained at       0.5 Check
PLAT300_ALERT_4_G Atom Site Occupancy of C61        Constrained at       0.5 Check
PLAT300_ALERT_4_G Atom Site Occupancy of H53A       Constrained at       0.5 Check
PLAT300_ALERT_4_G Atom Site Occupancy of H53B       Constrained at       0.5 Check
PLAT300_ALERT_4_G Atom Site Occupancy of H54A       Constrained at       0.5 Check
PLAT300_ALERT_4_G Atom Site Occupancy of H54B       Constrained at       0.5 Check
PLAT300_ALERT_4_G Atom Site Occupancy of H55A       Constrained at       0.5 Check
PLAT300_ALERT_4_G Atom Site Occupancy of H55B       Constrained at       0.5 Check
PLAT300_ALERT_4_G Atom Site Occupancy of H56        Constrained at       0.5 Check
PLAT300_ALERT_4_G Atom Site Occupancy of H57A       Constrained at       0.5 Check
PLAT300_ALERT_4_G Atom Site Occupancy of H57B       Constrained at       0.5 Check
PLAT300_ALERT_4_G Atom Site Occupancy of H58        Constrained at       0.5 Check
PLAT300_ALERT_4_G Atom Site Occupancy of H59A       Constrained at       0.5 Check
PLAT300_ALERT_4_G Atom Site Occupancy of H59B       Constrained at       0.5 Check
PLAT300_ALERT_4_G Atom Site Occupancy of H60        Constrained at       0.5 Check
PLAT300_ALERT_4_G Atom Site Occupancy of H61A       Constrained at       0.5 Check
PLAT300_ALERT_4_G Atom Site Occupancy of H61B       Constrained at       0.5 Check
PLAT302_ALERT_4_G Anion/Solvent/Minor-Residue Disorder (Resd  2  )      100% Note
PLAT411_ALERT_2_G Short Inter H...H Contact  H20A     ..H57B     .      2.03 Ang.
                                                    x,y,z  =     1_555 Check
PLAT606_ALERT_4_G Solvent Accessible VOID(S) in Structure ........         ! Info
PLAT791_ALERT_4_G Model has Chirality at C1          (Sohnke SpGr)         S Verify
PLAT791_ALERT_4_G Model has Chirality at C2          (Sohnke SpGr)         R Verify
PLAT791_ALERT_4_G Model has Chirality at C3          (Sohnke SpGr)         R Verify
PLAT791_ALERT_4_G Model has Chirality at C4          (Sohnke SpGr)         R Verify
PLAT791_ALERT_4_G Model has Chirality at C5          (Sohnke SpGr)         R Verify
PLAT791_ALERT_4_G Model has Chirality at C7          (Sohnke SpGr)         S Verify
PLAT791_ALERT_4_G Model has Chirality at C8          (Sohnke SpGr)         R Verify
PLAT791_ALERT_4_G Model has Chirality at C9          (Sohnke SpGr)         R Verify
PLAT791_ALERT_4_G Model has Chirality at C10         (Sohnke SpGr)         R Verify
PLAT791_ALERT_4_G Model has Chirality at C11         (Sohnke SpGr)         R Verify
PLAT791_ALERT_4_G Model has Chirality at C13         (Sohnke SpGr)         S Verify
PLAT791_ALERT_4_G Model has Chirality at C14         (Sohnke SpGr)         R Verify
PLAT791_ALERT_4_G Model has Chirality at C15         (Sohnke SpGr)         R Verify
PLAT791_ALERT_4_G Model has Chirality at C16         (Sohnke SpGr)         R Verify
PLAT791_ALERT_4_G Model has Chirality at C17         (Sohnke SpGr)         R Verify
PLAT791_ALERT_4_G Model has Chirality at C19         (Sohnke SpGr)         S Verify
PLAT791_ALERT_4_G Model has Chirality at C20         (Sohnke SpGr)         R Verify
PLAT791_ALERT_4_G Model has Chirality at C21         (Sohnke SpGr)         R Verify
PLAT791_ALERT_4_G Model has Chirality at C22         (Sohnke SpGr)         R Verify
PLAT791_ALERT_4_G Model has Chirality at C23         (Sohnke SpGr)         R Verify
PLAT791_ALERT_4_G Model has Chirality at C25         (Sohnke SpGr)         S Verify
PLAT791_ALERT_4_G Model has Chirality at C26         (Sohnke SpGr)         R Verify
PLAT791_ALERT_4_G Model has Chirality at C27         (Sohnke SpGr)         R Verify
PLAT791_ALERT_4_G Model has Chirality at C28         (Sohnke SpGr)         R Verify
PLAT791_ALERT_4_G Model has Chirality at C29         (Sohnke SpGr)         R Verify
PLAT791_ALERT_4_G Model has Chirality at C31         (Sohnke SpGr)         S Verify
PLAT791_ALERT_4_G Model has Chirality at C32         (Sohnke SpGr)         R Verify
PLAT791_ALERT_4_G Model has Chirality at C33         (Sohnke SpGr)         R Verify
PLAT791_ALERT_4_G Model has Chirality at C34         (Sohnke SpGr)         R Verify
PLAT791_ALERT_4_G Model has Chirality at C35         (Sohnke SpGr)         R Verify
PLAT791_ALERT_4_G Model has Chirality at C37         (Sohnke SpGr)         S Verify
PLAT791_ALERT_4_G Model has Chirality at C38         (Sohnke SpGr)         R Verify
PLAT791_ALERT_4_G Model has Chirality at C39         (Sohnke SpGr)         R Verify
PLAT791_ALERT_4_G Model has Chirality at C40         (Sohnke SpGr)         R Verify
PLAT791_ALERT_4_G Model has Chirality at C41         (Sohnke SpGr)         R Verify
PLAT850_ALERT_4_G Check Flack Parameter Exact Value 0.00 with s.u.      0.20 Check
PLAT860_ALERT_3_G Number of Least-Squares Restraints ............         18 Note
PLAT869_ALERT_4_G ALERTS Related to the Use of SQUEEZE Suppressed          ! Info
PLAT883_ALERT_1_G No Info/Value for _atom_sites_solution_primary .     Please Do !
```

---

1 **ALERT level A** = Most likely a serious problem - resolve or explain
10 **ALERT level B** = A potentially serious problem, consider carefully
7 **ALERT level C** = Check. Ensure it is not caused by an omission or oversight
77 **ALERT level G** = General information/check it is not something unexpected

4 ALERT type 1 CIF construction/syntax error, inconsistent or missing data
17 ALERT type 2 Indicator that the structure model may be wrong or deficient
3 ALERT type 3 Indicator that the structure quality may be low
70 ALERT type 4 Improvement, methodology, query or suggestion
1 ALERT type 5 Informative message, check

---

checkCIF publication errors

Alert level A

PUBL004_ALERT_1_A The contact author’s name and address are missing,
_publ_contact_author_name and _publ_contact_author_address.
PUBL005_ALERT_1_A _publ_contact_author_email, _publ_contact_author_fax and
_publ_contact_author_phone are all missing.
At least one of these should be present.
PUBL006_ALERT_1_A _publ_requested_journal is missing
e.g. ’Acta Crystallographica Section C’
PUBL008_ALERT_1_A _publ_section_title is missing. Title of paper.
PUBL009_ALERT_1_A _publ_author_name is missing. List of author(s) name(s).
PUBL010_ALERT_1_A _publ_author_address is missing. Author(s) address(es).
PUBL012_ALERT_1_A _publ_section_abstract is missing.
Abstract of paper in English.

Alert level G

PUBL017_ALERT_1_G The _publ_section_references section is missing or
empty.

---

7 **ALERT level A** = Data missing that is essential or data in wrong format
1 **ALERT level G** = General alerts. Data that may be required is missing

---

Publication of your CIF

You should attempt to resolve as many as possible of the alerts in all categories. Often the minor alerts point to easily fixed oversights, errors and omissions in your CIF or refinement strategy, so attention to these fine details can be worthwhile. In order to resolve some of the more serious problems it may be necessary to carry out additional measurements or structure refinements. However, the nature of your study may justify the reported deviations from journal submission requirements and the more serious of these should be commented upon in the discussion or experimental section of a paper or in the "special_details" fields of the CIF. *checkCIF* was carefully designed to identify outliers and unusual parameters, but every test has its limitations and alerts that are not important in a particular case may appear. Conversely, the absence of alerts does not guarantee there are no aspects of the results needing attention. It is up to the individual to critically assess their own results and, if necessary, seek expert advice.

If level A alerts remain, which you believe to be justified deviations, and you intend to submit this CIF for publication in a journal, you should additionally insert an explanation in your CIF using the Validation Reply Form (VRF) below. This will allow your explanation to be considered as part of the review process.

Validation response form

Please find below a validation response form (VRF) that can be filled in and pasted into your CIF.

```
# start Validation Reply Form
_vrf_PUBL004_GLOBAL
;
PROBLEM: The contact author’s name and address are missing,
RESPONSE: ...
;
_vrf_PUBL005_GLOBAL
;
PROBLEM: _publ_contact_author_email, _publ_contact_author_fax and
RESPONSE: ...
;
_vrf_PUBL006_GLOBAL
;
PROBLEM: _publ_requested_journal is missing
RESPONSE: ...
;
_vrf_PUBL008_GLOBAL
;
PROBLEM: _publ_section_title is missing. Title of paper.
RESPONSE: ...
;
_vrf_PUBL009_GLOBAL
;
PROBLEM: _publ_author_name is missing. List of author(s) name(s).
RESPONSE: ...
;
_vrf_PUBL010_GLOBAL
;
PROBLEM: _publ_author_address is missing. Author(s) address(es).
RESPONSE: ...
;
_vrf_PUBL012_GLOBAL
;
PROBLEM: _publ_section_abstract is missing.
```

```
RESPONSE: ...
;
_vrf_PLAT097_I
;
PROBLEM: Large Reported Max.  (Positive) Residual Density       1.82 eA-3
RESPONSE: ...
;
# end Validation Reply Form
```

If you wish to submit your CIF for publication in Acta Crystallographica Section C or E, you should upload your CIF via the web. If you wish to submit your CIF for publication in IUCrData you should upload your CIF via the web. If your CIF is to form part of a submission to another IUCr journal, you will be asked, either during electronic submission or by the Co-editor handling your paper, to upload your CIF via our web site.

PLATON version of 22/03/2021; check.def file version of 19/03/2021

Datablock I - ellipsoid plot

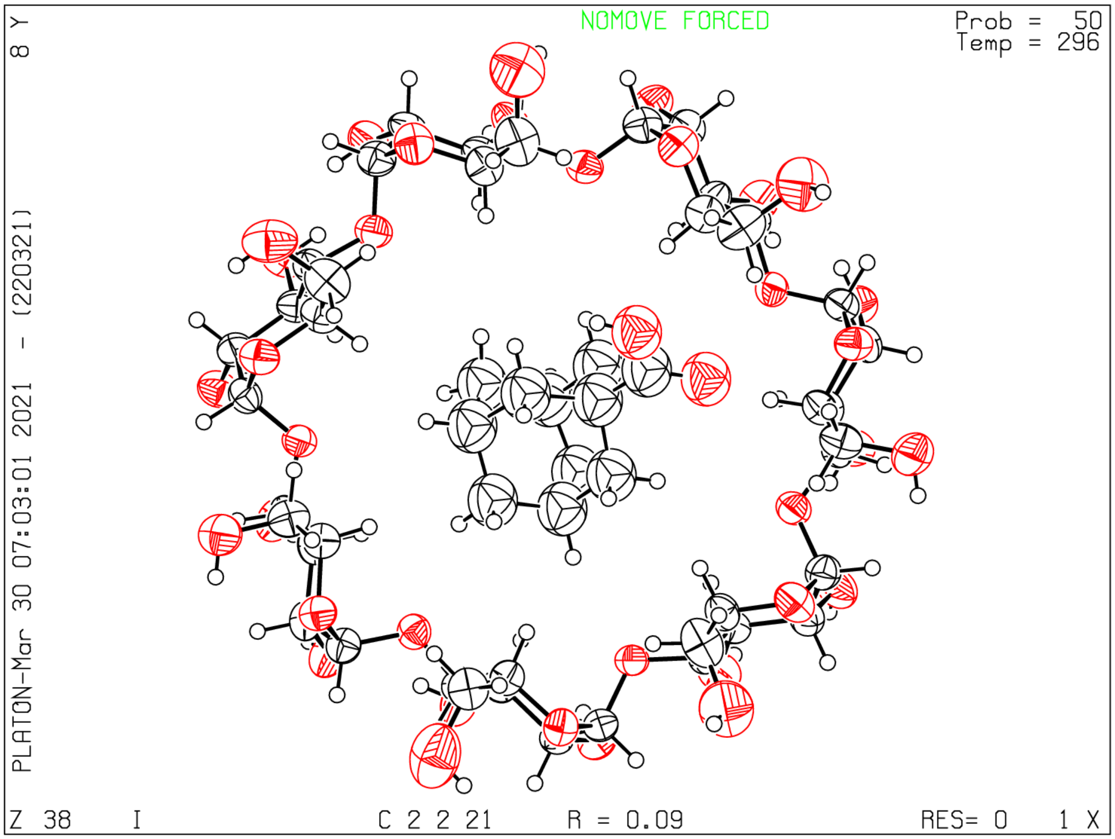

Supplement: Supplementary file 1 [file molecules-26-02412-s001.zip › CIF and CheckCIF/CheckCIF_Complex 4.pdf]
