# Supplementary material for: Structural Insights into the Host–Guest Complexation between β-Cyclodextrin and Bio-Conjugatable Adamantane Derivatives
Source: Molecules. 2021 Apr 21;26(9):2412. doi: 10.3390/molecules26092412 (PMC8122645; doi:10.3390/molecules26092412)

checkCIF/PLATON report

You have not supplied any structure factors. As a result the full set of tests cannot be run.

THIS REPORT IS FOR GUIDANCE ONLY. IF USED AS PART OF A REVIEW PROCEDURE FOR PUBLICATION, IT SHOULD NOT REPLACE THE EXPERTISE OF AN EXPERIENCED CRYSTALLOGRAPHIC REFEREE.

No syntax errors found. CIF dictionary Interpreting this report

Datablock: I

Bond precision: C-C = 0.0094 A Wavelength=0.71073

Cell:	a=15.2993(8)	b=15.4429(7)	c=18.0837(10)
	alpha=99.751(4)	beta=113.551(5)	gamma=102.813(4)
Temperature:	150 K		

	Calculated	Reported
Volume	3656.6(4)	3656.6(4)
Space group	P 1	P 1
Hall group	P 1	P 1
Moiety formula	2(C42 H70 O35), C12 H16 O4 [+ solvent]	2(C42 H70 O35), C12 H16 O4 [+ SOLVENT]
Sum formula	C96 H156 O74 [+ solvent]	C96 H156 O74
Mr	2494.22	2494.20
Dx,g cm-3	1.133	1.133
Z	1	1
Mu (mm-1)	0.099	0.099
F000	1324.0	1324.0
F000’	1324.97	
h,k,lmax	21,21,24	21,20,24
Nref	40254[20127]	21115
Tmin,Tmax	0.991,0.992	0.991,0.992
Tmin’	0.990	

Correction method= # Reported T Limits: Tmin=0.991 Tmax=0.992
AbsCorr = MULTI-SCAN

Data completeness= 1.05/0.52 Theta(max)= 29.369

R(reflections)= 0.0758(16593) wR2(reflections)= 0.2305(21115)

S = 1.027 Npar= 1676

The following ALERTS were generated. Each ALERT has the format
test-name_ALERT_alert-type_alert-level.
Click on the hyperlinks for more details of the test.

Alert level B

```
PLAT097_ALERT_2_B Large Reported Max.  (Positive) Residual Density       1.04 eA-3
PLAT415_ALERT_2_B Short Inter D-H..H-X       H53A     ..H58O     .       1.91 Ang.
                                                -1+x,y,z  =      1_455 Check
PLAT415_ALERT_2_B Short Inter D-H..H-X       H54A     ..H64O     .       1.93 Ang.
                                                -1+x,y,z  =      1_455 Check
PLAT420_ALERT_2_B D-H Bond Without Acceptor  O3       --H3O      .     Please Check
PLAT420_ALERT_2_B D-H Bond Without Acceptor  O6       --H6O      .     Please Check
PLAT420_ALERT_2_B D-H Bond Without Acceptor  O9       --H9O      .     Please Check
PLAT420_ALERT_2_B D-H Bond Without Acceptor  O13      --H13O     .     Please Check
PLAT420_ALERT_2_B D-H Bond Without Acceptor  O16      --H16O     .     Please Check
PLAT420_ALERT_2_B D-H Bond Without Acceptor  O23      --H23O     .     Please Check
PLAT420_ALERT_2_B D-H Bond Without Acceptor  O24      --H24O     .     Please Check
PLAT420_ALERT_2_B D-H Bond Without Acceptor  O26      --H26O     .     Please Check
PLAT420_ALERT_2_B D-H Bond Without Acceptor  O28      --H28      .     Please Check
PLAT420_ALERT_2_B D-H Bond Without Acceptor  O34      --H34O     .     Please Check
PLAT420_ALERT_2_B D-H Bond Without Acceptor  O38      --H38O     .     Please Check
PLAT420_ALERT_2_B D-H Bond Without Acceptor  O39      --H39O     .     Please Check
PLAT420_ALERT_2_B D-H Bond Without Acceptor  O41      --H41O     .     Please Check
PLAT420_ALERT_2_B D-H Bond Without Acceptor  O43      --H43O     .     Please Check
PLAT420_ALERT_2_B D-H Bond Without Acceptor  O53      --H53O     .     Please Check
PLAT420_ALERT_2_B D-H Bond Without Acceptor  O56      --H56O     .     Please Check
PLAT420_ALERT_2_B D-H Bond Without Acceptor  O59      --H59O     .     Please Check
PLAT420_ALERT_2_B D-H Bond Without Acceptor  O63      --H63O     .     Please Check
PLAT420_ALERT_2_B D-H Bond Without Acceptor  O64      --H64O     .     Please Check
PLAT420_ALERT_2_B D-H Bond Without Acceptor  O66      --H66O     .     Please Check
PLAT420_ALERT_2_B D-H Bond Without Acceptor  O68      --H68O     .     Please Check
PLAT420_ALERT_2_B D-H Bond Without Acceptor  O74A     --H74W     .     Please Check
```

Alert level C

```
DIFMX02_ALERT_1_C  The maximum difference density is > 0.1*ZMAX*0.75
            The relevant atom site should be identified.
STRVA01_ALERT_4_C           Flack test results are ambiguous.
           From the CIF: _refine_ls_abs_structure_Flack    0.600
           From the CIF: _refine_ls_abs_structure_Flack_su    0.600
PLAT094_ALERT_2_C Ratio of Maximum / Minimum Residual Density ....       2.55 Report
PLAT309_ALERT_2_C Single Bonded Oxygen (C-O > 1.3 Ang) ..........       O72A Check
PLAT340_ALERT_3_C Low Bond Precision on  C-C Bonds ..............    0.00936 Ang.
PLAT415_ALERT_2_C Short Inter D-H..H-X       H54O     ..H84A     .       2.05 Ang.
                                                 x,1+y,z  =      1_565 Check
PLAT761_ALERT_1_C CIF Contains no X-H Bonds .....................     Please Check
PLAT762_ALERT_1_C CIF Contains no X-Y-H or H-Y-H Angles ..........     Please Check
PLAT907_ALERT_2_C Flack x > 0.5, Structure Needs to be Inverted? .       0.60 Check
```

Alert level G

```
PLAT002_ALERT_2_G Number of Distance or Angle Restraints on AtSite         20 Note
PLAT003_ALERT_2_G Number of Uiso or Uij Restrained non-H Atoms ...         32 Report
PLAT007_ALERT_5_G Number of Unrefined Donor-H Atoms .............         46 Report
PLAT032_ALERT_4_G Std. Uncertainty on Flack Parameter Value High .      0.600 Report
PLAT066_ALERT_1_G Predicted and Reported Tmin&Tmax Range Identical          ? Check
PLAT175_ALERT_4_G The CIF-Embedded .res File Contains SAME Records          1 Report
PLAT177_ALERT_4_G The CIF-Embedded .res File Contains DELU Records          1 Report
PLAT178_ALERT_4_G The CIF-Embedded .res File Contains SIMU Records          1 Report
PLAT302_ALERT_4_G Anion/Solvent/Minor-Residue Disorder (Resd  3  )       100% Note
```

PLAT302_ALERT_4_G Anion/Solvent/Minor-Residue Disorder (Resd 4) 100% Note
PLAT304_ALERT_4_G Non-Integer Number of Atoms in ..... (Resd 3) 16.42 Check
PLAT304_ALERT_4_G Non-Integer Number of Atoms in ..... (Resd 4) 15.58 Check
PLAT417_ALERT_2_G Short Inter D-H..H-D H8O ..H73W . 1.98 Ang.
x,1+y,z = 1_565 Check
PLAT606_ALERT_4_G Solvent Accessible VOID(S) in Structure .... ! Info
PLAT720_ALERT_4_G Number of Unusual/Non-Standard Labels .... 1 Note
PLAT791_ALERT_4_G Model has Chirality at C2 (Sohnke SpGr) R Verify
PLAT791_ALERT_4_G Model has Chirality at C3 (Sohnke SpGr) S Verify
PLAT791_ALERT_4_G Model has Chirality at C4 (Sohnke SpGr) R Verify
PLAT791_ALERT_4_G Model has Chirality at C5 (Sohnke SpGr) R Verify
PLAT791_ALERT_4_G Model has Chirality at C6 (Sohnke SpGr) R Verify
PLAT791_ALERT_4_G Model has Chirality at C8 (Sohnke SpGr) R Verify
PLAT791_ALERT_4_G Model has Chirality at C9 (Sohnke SpGr) S Verify
PLAT791_ALERT_4_G Model has Chirality at C10 (Sohnke SpGr) R Verify
PLAT791_ALERT_4_G Model has Chirality at C11 (Sohnke SpGr) R Verify
PLAT791_ALERT_4_G Model has Chirality at C12 (Sohnke SpGr) R Verify
PLAT791_ALERT_4_G Model has Chirality at C14 (Sohnke SpGr) R Verify
PLAT791_ALERT_4_G Model has Chirality at C15 (Sohnke SpGr) S Verify
PLAT791_ALERT_4_G Model has Chirality at C16 (Sohnke SpGr) R Verify
PLAT791_ALERT_4_G Model has Chirality at C17 (Sohnke SpGr) R Verify
PLAT791_ALERT_4_G Model has Chirality at C18 (Sohnke SpGr) R Verify
PLAT791_ALERT_4_G Model has Chirality at C20 (Sohnke SpGr) R Verify
PLAT791_ALERT_4_G Model has Chirality at C21 (Sohnke SpGr) S Verify
PLAT791_ALERT_4_G Model has Chirality at C22 (Sohnke SpGr) R Verify
PLAT791_ALERT_4_G Model has Chirality at C23 (Sohnke SpGr) R Verify
PLAT791_ALERT_4_G Model has Chirality at C24 (Sohnke SpGr) R Verify
PLAT791_ALERT_4_G Model has Chirality at C26 (Sohnke SpGr) R Verify
PLAT791_ALERT_4_G Model has Chirality at C27 (Sohnke SpGr) S Verify
PLAT791_ALERT_4_G Model has Chirality at C28 (Sohnke SpGr) R Verify
PLAT791_ALERT_4_G Model has Chirality at C29 (Sohnke SpGr) R Verify
PLAT791_ALERT_4_G Model has Chirality at C30 (Sohnke SpGr) R Verify
PLAT791_ALERT_4_G Model has Chirality at C32 (Sohnke SpGr) R Verify
PLAT791_ALERT_4_G Model has Chirality at C33 (Sohnke SpGr) S Verify
PLAT791_ALERT_4_G Model has Chirality at C34 (Sohnke SpGr) R Verify
PLAT791_ALERT_4_G Model has Chirality at C35 (Sohnke SpGr) R Verify
PLAT791_ALERT_4_G Model has Chirality at C36 (Sohnke SpGr) R Verify
PLAT791_ALERT_4_G Model has Chirality at C38 (Sohnke SpGr) R Verify
PLAT791_ALERT_4_G Model has Chirality at C39 (Sohnke SpGr) S Verify
PLAT791_ALERT_4_G Model has Chirality at C40 (Sohnke SpGr) R Verify
PLAT791_ALERT_4_G Model has Chirality at C41 (Sohnke SpGr) R Verify
PLAT791_ALERT_4_G Model has Chirality at C42 (Sohnke SpGr) R Verify
PLAT791_ALERT_4_G Model has Chirality at C44 (Sohnke SpGr) R Verify
PLAT791_ALERT_4_G Model has Chirality at C45 (Sohnke SpGr) S Verify
PLAT791_ALERT_4_G Model has Chirality at C46 (Sohnke SpGr) R Verify
PLAT791_ALERT_4_G Model has Chirality at C47 (Sohnke SpGr) R Verify
PLAT791_ALERT_4_G Model has Chirality at C48 (Sohnke SpGr) R Verify
PLAT791_ALERT_4_G Model has Chirality at C50 (Sohnke SpGr) R Verify
PLAT791_ALERT_4_G Model has Chirality at C51 (Sohnke SpGr) S Verify
PLAT791_ALERT_4_G Model has Chirality at C52 (Sohnke SpGr) R Verify
PLAT791_ALERT_4_G Model has Chirality at C53 (Sohnke SpGr) R Verify
PLAT791_ALERT_4_G Model has Chirality at C54 (Sohnke SpGr) R Verify
PLAT791_ALERT_4_G Model has Chirality at C56 (Sohnke SpGr) R Verify
PLAT791_ALERT_4_G Model has Chirality at C57 (Sohnke SpGr) S Verify
PLAT791_ALERT_4_G Model has Chirality at C58 (Sohnke SpGr) R Verify
PLAT791_ALERT_4_G Model has Chirality at C59 (Sohnke SpGr) R Verify
PLAT791_ALERT_4_G Model has Chirality at C60 (Sohnke SpGr) R Verify
PLAT791_ALERT_4_G Model has Chirality at C62 (Sohnke SpGr) R Verify
PLAT791_ALERT_4_G Model has Chirality at C63 (Sohnke SpGr) S Verify
PLAT791_ALERT_4_G Model has Chirality at C64 (Sohnke SpGr) R Verify
PLAT791_ALERT_4_G Model has Chirality at C65 (Sohnke SpGr) R Verify
PLAT791_ALERT_4_G Model has Chirality at C66 (Sohnke SpGr) R Verify

```
PLAT791_ALERT_4_G Model has Chirality at C68         (Sohnke SpGr)          R Verify
PLAT791_ALERT_4_G Model has Chirality at C69         (Sohnke SpGr)          S Verify
PLAT791_ALERT_4_G Model has Chirality at C70         (Sohnke SpGr)          R Verify
PLAT791_ALERT_4_G Model has Chirality at C71         (Sohnke SpGr)          R Verify
PLAT791_ALERT_4_G Model has Chirality at C72         (Sohnke SpGr)          R Verify
PLAT791_ALERT_4_G Model has Chirality at C74         (Sohnke SpGr)          R Verify
PLAT791_ALERT_4_G Model has Chirality at C75         (Sohnke SpGr)          S Verify
PLAT791_ALERT_4_G Model has Chirality at C76         (Sohnke SpGr)          R Verify
PLAT791_ALERT_4_G Model has Chirality at C77         (Sohnke SpGr)          R Verify
PLAT791_ALERT_4_G Model has Chirality at C78         (Sohnke SpGr)          R Verify
PLAT791_ALERT_4_G Model has Chirality at C80         (Sohnke SpGr)          R Verify
PLAT791_ALERT_4_G Model has Chirality at C81         (Sohnke SpGr)          S Verify
PLAT791_ALERT_4_G Model has Chirality at C82         (Sohnke SpGr)          R Verify
PLAT791_ALERT_4_G Model has Chirality at C83         (Sohnke SpGr)          R Verify
PLAT791_ALERT_4_G Model has Chirality at C84         (Sohnke SpGr)          R Verify
PLAT791_ALERT_4_G Model has Chirality at C93A        (Sohnke SpGr)          R Verify
PLAT791_ALERT_4_G Model has Chirality at C95A        (Sohnke SpGr)          S Verify
PLAT860_ALERT_3_G Number of Least-Squares Restraints .............        447 Note
PLAT869_ALERT_4_G ALERTS Related to the Use of SQUEEZE Suppressed           ! Info
PLAT883_ALERT_1_G No Info/Value for _atom_sites_solution_primary .     Please Do !
PLAT941_ALERT_3_G Average HKL Measurement Multiplicity ...........        1.6 Low
PLAT965_ALERT_2_G The SHELXL WEIGHT Optimisation has not Converged     Please Check
```

```
   0 ALERT level A = Most likely a serious problem - resolve or explain
  25 ALERT level B = A potentially serious problem, consider carefully
   9 ALERT level C = Check. Ensure it is not caused by an omission or oversight
  92 ALERT level G = General information/check it is not something unexpected

   5 ALERT type 1 CIF construction/syntax error, inconsistent or missing data
  33 ALERT type 2 Indicator that the structure model may be wrong or deficient
   3 ALERT type 3 Indicator that the structure quality may be low
  84 ALERT type 4 Improvement, methodology, query or suggestion
   1 ALERT type 5 Informative message, check
```

---

checkCIF publication errors

Alert level A

```
PUBL004_ALERT_1_A  The contact author’s name and address are missing,
            _publ_contact_author_name and _publ_contact_author_address.
PUBL005_ALERT_1_A  _publ_contact_author_email, _publ_contact_author_fax and
            _publ_contact_author_phone are all missing.
            At least one of these should be present.
PUBL006_ALERT_1_A  _publ_requested_journal is missing
            e.g. ’Acta Crystallographica Section C’
PUBL008_ALERT_1_A  _publ_section_title is missing. Title of paper.
PUBL009_ALERT_1_A  _publ_author_name is missing. List of author(s) name(s).
PUBL010_ALERT_1_A  _publ_author_address is missing. Author(s) address(es).
PUBL012_ALERT_1_A  _publ_section_abstract is missing.
            Abstract of paper in English.
```

Alert level G

```
PUBL017_ALERT_1_G The _publ_section_references section is missing or
            empty.
```

```
  7 ALERT level A = Data missing that is essential or data in wrong format
  1 ALERT level G = General alerts. Data that may be required is missing
```

Publication of your CIF

You should attempt to resolve as many as possible of the alerts in all categories. Often the minor alerts point to easily fixed oversights, errors and omissions in your CIF or refinement strategy, so attention to these fine details can be worthwhile. In order to resolve some of the more serious problems it may be necessary to carry out additional measurements or structure refinements. However, the nature of your study may justify the reported deviations from journal submission requirements and the more serious of these should be commented upon in the discussion or experimental section of a paper or in the "special_details" fields of the CIF. *checkCIF* was carefully designed to identify outliers and unusual parameters, but every test has its limitations and alerts that are not important in a particular case may appear. Conversely, the absence of alerts does not guarantee there are no aspects of the results needing attention. It is up to the individual to critically assess their own results and, if necessary, seek expert advice.

If level A alerts remain, which you believe to be justified deviations, and you intend to submit this CIF for publication in a journal, you should additionally insert an explanation in your CIF using the Validation Reply Form (VRF) below. This will allow your explanation to be considered as part of the review process.

Validation response form

Please find below a validation response form (VRF) that can be filled in and pasted into your CIF.

```
# start Validation Reply Form
_vrf_PUBL004_GLOBAL
;
PROBLEM: The contact author’s name and address are missing,
RESPONSE: ...
;
_vrf_PUBL005_GLOBAL
;
PROBLEM: _publ_contact_author_email, _publ_contact_author_fax and
RESPONSE: ...
;
_vrf_PUBL006_GLOBAL
;
PROBLEM: _publ_requested_journal is missing
RESPONSE: ...
;
_vrf_PUBL008_GLOBAL
;
PROBLEM: _publ_section_title is missing. Title of paper.
RESPONSE: ...
;
_vrf_PUBL009_GLOBAL
;
PROBLEM: _publ_author_name is missing. List of author(s) name(s).
RESPONSE: ...
;
_vrf_PUBL010_GLOBAL
;
PROBLEM: _publ_author_address is missing. Author(s) address(es).
```

```
RESPONSE: ...
;
_vrf_PUBL012_GLOBAL
;
PROBLEM: _publ_section_abstract is missing.
RESPONSE: ...
;
# end Validation Reply Form
```

If you wish to submit your CIF for publication in Acta Crystallographica Section C or E, you should upload your CIF via the web. If you wish to submit your CIF for publication in IUCrData you should upload your CIF via the web. If your CIF is to form part of a submission to another IUCr journal, you will be asked, either during electronic submission or by the Co-editor handling your paper, to upload your CIF via our web site.

PLATON version of 22/03/2021; check.def file version of 19/03/2021

Datablock I - ellipsoid plot

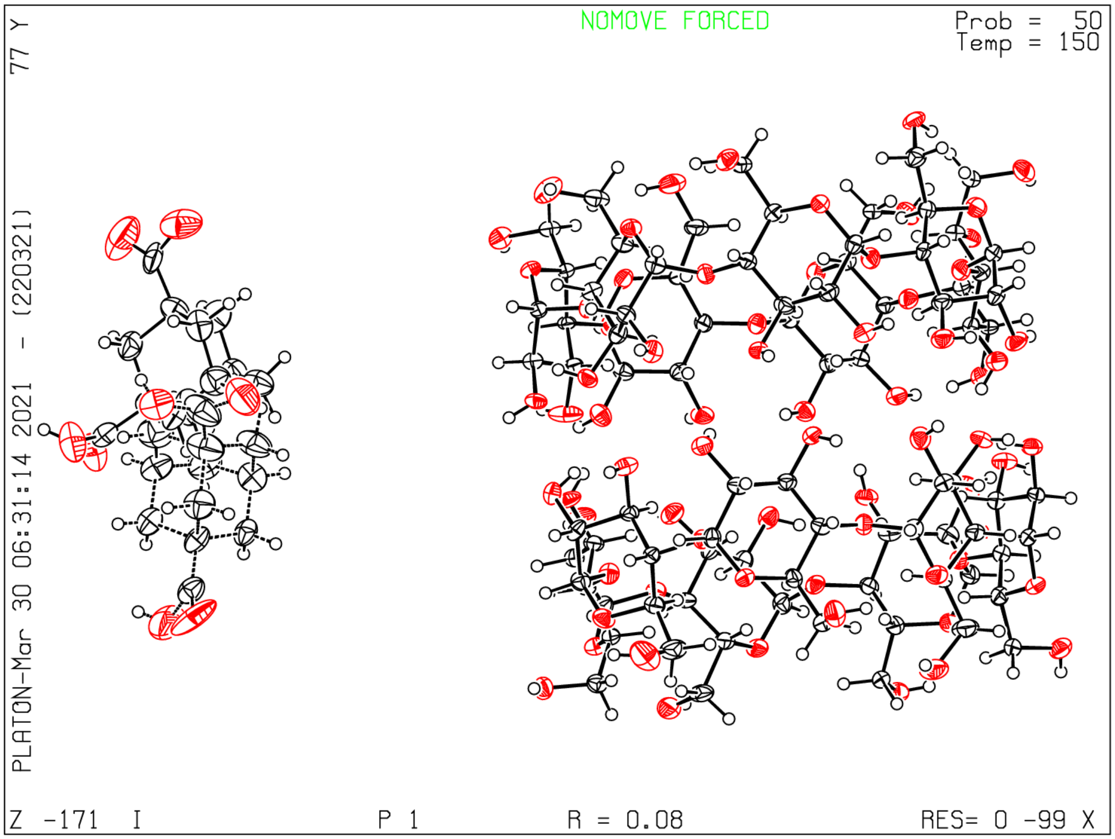

Supplement: Supplementary file 1 [file molecules-26-02412-s001.zip › CIF and CheckCIF/CheckCIF_Complex 5.pdf]
