# Supplementary material for: Structural Insights into the Host–Guest Complexation between β-Cyclodextrin and Bio-Conjugatable Adamantane Derivatives
Source: Molecules. 2021 Apr 21;26(9):2412. doi: 10.3390/molecules26092412 (PMC8122645; doi:10.3390/molecules26092412)

checkCIF/PLATON report

You have not supplied any structure factors. As a result the full set of tests cannot be run.

THIS REPORT IS FOR GUIDANCE ONLY. IF USED AS PART OF A REVIEW PROCEDURE FOR PUBLICATION, IT SHOULD NOT REPLACE THE EXPERTISE OF AN EXPERIENCED CRYSTALLOGRAPHIC REFEREE.

No syntax errors found. CIF dictionary Interpreting this report

Datablock: I

```
Bond precision:  C-C = 0.0064 A          Wavelength=1.34180

Cell:          a=15.2868(3)       b=15.4435(3)      c=18.0918(4)
               alpha=100.050(1)   beta=113.033(1)   gamma=102.490(1)
Temperature:   170 K

                Calculated                    Reported
Volume          3674.57(14)                   3674.56(13)
Space group     P 1                           P 1
Hall group      P 1                           P 1
Moiety formula  C42 H70 O35 [+ solvent]       ?
Sum formula     C42 H70 O35 [+ solvent]       C84 H140 O70
Mr              1134.98                       2269.95
Dx,g cm-3       1.026                         1.026
Z               2                             1
Mu (mm-1)       0.501                         0.504
F000            1204.0                        1204.0
F000'           1207.76
h,k,lmax        19,19,22                      19,19,22
Nref            30126[ 15063]                 27008
Tmin,Tmax       0.860,0.895                   0.860,0.895
Tmin'           0.860

Correction method= # Reported T Limits: Tmin=0.860 Tmax=0.895
AbsCorr = MULTI-SCAN

Data completeness= 1.79/0.90      Theta(max)= 57.090

R(reflections)= 0.0648( 25912)    wR2(reflections)= 0.1912( 27008)

S = 1.028                  Npar= 1387
```

The following ALERTS were generated. Each ALERT has the format
test-name_ALERT_alert-type_alert-level.
Click on the hyperlinks for more details of the test.

Alert level B

```
PLAT097_ALERT_2_B Large Reported Max.  (Positive) Residual Density       1.48 eA-3
PLAT415_ALERT_2_B Short Inter D-H..H-X       H6A      ..H24      .       1.99 Ang.
                                                  x,1+y,z  =      1_565 Check
PLAT420_ALERT_2_B D-H Bond Without Acceptor  O1       --H1       .     Please Check
PLAT420_ALERT_2_B D-H Bond Without Acceptor  O3       --H3       .     Please Check
PLAT420_ALERT_2_B D-H Bond Without Acceptor  O9       --H9       .     Please Check
PLAT420_ALERT_2_B D-H Bond Without Acceptor  O13      --H13      .     Please Check
PLAT420_ALERT_2_B D-H Bond Without Acceptor  O16      --H16      .     Please Check
PLAT420_ALERT_2_B D-H Bond Without Acceptor  O18      --H18      .     Please Check
PLAT420_ALERT_2_B D-H Bond Without Acceptor  O23      --H23      .     Please Check
PLAT420_ALERT_2_B D-H Bond Without Acceptor  O24      --H24      .     Please Check
PLAT420_ALERT_2_B D-H Bond Without Acceptor  O26      --H26      .     Please Check
PLAT420_ALERT_2_B D-H Bond Without Acceptor  O28      --H28      .     Please Check
PLAT420_ALERT_2_B D-H Bond Without Acceptor  O31      --H31      .     Please Check
PLAT420_ALERT_2_B D-H Bond Without Acceptor  O33      --H33      .     Please Check
PLAT420_ALERT_2_B D-H Bond Without Acceptor  O34      --H34      .     Please Check
PLAT420_ALERT_2_B D-H Bond Without Acceptor  O39      --H39      .     Please Check
PLAT420_ALERT_2_B D-H Bond Without Acceptor  O43      --H43      .     Please Check
PLAT420_ALERT_2_B D-H Bond Without Acceptor  O46      --H46      .     Please Check
PLAT420_ALERT_2_B D-H Bond Without Acceptor  O51      --H51      .     Please Check
PLAT420_ALERT_2_B D-H Bond Without Acceptor  O54      --H54      .     Please Check
PLAT420_ALERT_2_B D-H Bond Without Acceptor  O56      --H56      .     Please Check
PLAT420_ALERT_2_B D-H Bond Without Acceptor  O58      --H58      .     Please Check
PLAT420_ALERT_2_B D-H Bond Without Acceptor  O63      --H63      .     Please Check
PLAT420_ALERT_2_B D-H Bond Without Acceptor  O64      --H64      .     Please Check
PLAT420_ALERT_2_B D-H Bond Without Acceptor  O66      --H66      .     Please Check
PLAT420_ALERT_2_B D-H Bond Without Acceptor  O68      --H68      .     Please Check
```

Alert level C

```
DIFMX02_ALERT_1_C  The maximum difference density is > 0.1*ZMAX*0.75
            The relevant atom site should be identified.
RADNW01_ALERT_1_C  The radiation wavelength lies outside the expected range
            for the supplied radiation type. Expected range 1.34130-1.34150
            Wavelength given =    1.34180
PLAT048_ALERT_1_C MoietyFormula Not Given (or Incomplete) ........     Please Check
PLAT094_ALERT_2_C Ratio of Maximum / Minimum Residual Density ....       2.97 Report
PLAT220_ALERT_2_C NonSolvent   Resd 1  C   Ueq(max)/Ueq(min) Range        3.1 Ratio
PLAT220_ALERT_2_C NonSolvent   Resd 1  O   Ueq(max)/Ueq(min) Range        5.1 Ratio
PLAT220_ALERT_2_C NonSolvent   Resd 2  O   Ueq(max)/Ueq(min) Range        3.8 Ratio
PLAT222_ALERT_3_C NonSolvent Resd 1  H   Uiso(max)/Uiso(min) Range        5.7 Ratio
PLAT222_ALERT_3_C NonSolvent Resd 2  H   Uiso(max)/Uiso(min) Range        4.3 Ratio
PLAT234_ALERT_4_C Large Hirshfeld Difference O31      --C37      .       0.18 Ang.
PLAT242_ALERT_2_C Low    'MainMol' Ueq as Compared to Neighbors of        C73 Check
PLAT340_ALERT_3_C Low Bond Precision on  C-C Bonds ...............    0.00636 Ang.
PLAT415_ALERT_2_C Short Inter D-H..H-X       H54      ..H84A     .       2.08 Ang.
                                                  1+x,y,z  =      1_655 Check
```

Alert level G

```
ABSMU01_ALERT_1_G  Calculation of _exptl_absorpt_correction_mu
                not performed for this radiation type.
PLAT002_ALERT_2_G Number of Distance or Angle Restraints on AtSite          2 Note
PLAT007_ALERT_5_G Number of Unrefined Donor-H Atoms .............          42 Report
PLAT033_ALERT_4_G Flack x Value Deviates > 3.0 * sigma from Zero .      0.260 Note
PLAT045_ALERT_1_G Calculated and Reported Z Differ by a Factor ...       2.00 Check
PLAT066_ALERT_1_G Predicted and Reported Tmin&Tmax Range Identical          ? Check
PLAT154_ALERT_1_G The s.u.'s on the Cell Angles are Equal ..(Note)      0.001 Degree
PLAT172_ALERT_4_G The CIF-Embedded .res File Contains DFIX Records          1 Report
PLAT606_ALERT_4_G Solvent Accessible VOID(S) in Structure ........          ! Info
```

```
PLAT790_ALERT_4_G Centre of Gravity not Within Unit Cell: Resd.  #          2 Note
              C42 H70 O35
PLAT791_ALERT_4_G Model has Chirality at C2          (Sohnke SpGr)          R Verify
PLAT791_ALERT_4_G Model has Chirality at C3          (Sohnke SpGr)          S Verify
PLAT791_ALERT_4_G Model has Chirality at C4          (Sohnke SpGr)          R Verify
PLAT791_ALERT_4_G Model has Chirality at C5          (Sohnke SpGr)          R Verify
PLAT791_ALERT_4_G Model has Chirality at C6          (Sohnke SpGr)          R Verify
PLAT791_ALERT_4_G Model has Chirality at C8          (Sohnke SpGr)          R Verify
PLAT791_ALERT_4_G Model has Chirality at C9          (Sohnke SpGr)          S Verify
PLAT791_ALERT_4_G Model has Chirality at C10         (Sohnke SpGr)          R Verify
PLAT791_ALERT_4_G Model has Chirality at C11         (Sohnke SpGr)          R Verify
PLAT791_ALERT_4_G Model has Chirality at C12         (Sohnke SpGr)          R Verify
PLAT791_ALERT_4_G Model has Chirality at C14         (Sohnke SpGr)          R Verify
PLAT791_ALERT_4_G Model has Chirality at C15         (Sohnke SpGr)          S Verify
PLAT791_ALERT_4_G Model has Chirality at C16         (Sohnke SpGr)          R Verify
PLAT791_ALERT_4_G Model has Chirality at C17         (Sohnke SpGr)          R Verify
PLAT791_ALERT_4_G Model has Chirality at C18         (Sohnke SpGr)          R Verify
PLAT791_ALERT_4_G Model has Chirality at C20         (Sohnke SpGr)          R Verify
PLAT791_ALERT_4_G Model has Chirality at C21         (Sohnke SpGr)          S Verify
PLAT791_ALERT_4_G Model has Chirality at C22         (Sohnke SpGr)          R Verify
PLAT791_ALERT_4_G Model has Chirality at C23         (Sohnke SpGr)          R Verify
PLAT791_ALERT_4_G Model has Chirality at C24         (Sohnke SpGr)          R Verify
PLAT791_ALERT_4_G Model has Chirality at C26         (Sohnke SpGr)          R Verify
PLAT791_ALERT_4_G Model has Chirality at C27         (Sohnke SpGr)          S Verify
PLAT791_ALERT_4_G Model has Chirality at C28         (Sohnke SpGr)          R Verify
PLAT791_ALERT_4_G Model has Chirality at C29         (Sohnke SpGr)          R Verify
PLAT791_ALERT_4_G Model has Chirality at C30         (Sohnke SpGr)          R Verify
PLAT791_ALERT_4_G Model has Chirality at C32         (Sohnke SpGr)          R Verify
PLAT791_ALERT_4_G Model has Chirality at C33         (Sohnke SpGr)          S Verify
PLAT791_ALERT_4_G Model has Chirality at C34         (Sohnke SpGr)          R Verify
PLAT791_ALERT_4_G Model has Chirality at C35         (Sohnke SpGr)          R Verify
PLAT791_ALERT_4_G Model has Chirality at C36         (Sohnke SpGr)          R Verify
PLAT791_ALERT_4_G Model has Chirality at C38         (Sohnke SpGr)          R Verify
PLAT791_ALERT_4_G Model has Chirality at C39         (Sohnke SpGr)          S Verify
PLAT791_ALERT_4_G Model has Chirality at C40         (Sohnke SpGr)          R Verify
PLAT791_ALERT_4_G Model has Chirality at C41         (Sohnke SpGr)          R Verify
PLAT791_ALERT_4_G Model has Chirality at C42         (Sohnke SpGr)          R Verify
PLAT791_ALERT_4_G Model has Chirality at C44         (Sohnke SpGr)          R Verify
PLAT791_ALERT_4_G Model has Chirality at C45         (Sohnke SpGr)          S Verify
PLAT791_ALERT_4_G Model has Chirality at C46         (Sohnke SpGr)          R Verify
PLAT791_ALERT_4_G Model has Chirality at C47         (Sohnke SpGr)          R Verify
PLAT791_ALERT_4_G Model has Chirality at C48         (Sohnke SpGr)          R Verify
PLAT791_ALERT_4_G Model has Chirality at C50         (Sohnke SpGr)          R Verify
PLAT791_ALERT_4_G Model has Chirality at C51         (Sohnke SpGr)          S Verify
PLAT791_ALERT_4_G Model has Chirality at C52         (Sohnke SpGr)          R Verify
PLAT791_ALERT_4_G Model has Chirality at C53         (Sohnke SpGr)          R Verify
PLAT791_ALERT_4_G Model has Chirality at C54         (Sohnke SpGr)          R Verify
PLAT791_ALERT_4_G Model has Chirality at C56         (Sohnke SpGr)          R Verify
PLAT791_ALERT_4_G Model has Chirality at C57         (Sohnke SpGr)          S Verify
PLAT791_ALERT_4_G Model has Chirality at C58         (Sohnke SpGr)          R Verify
PLAT791_ALERT_4_G Model has Chirality at C59         (Sohnke SpGr)          R Verify
PLAT791_ALERT_4_G Model has Chirality at C60         (Sohnke SpGr)          R Verify
PLAT791_ALERT_4_G Model has Chirality at C62         (Sohnke SpGr)          R Verify
PLAT791_ALERT_4_G Model has Chirality at C63         (Sohnke SpGr)          S Verify
PLAT791_ALERT_4_G Model has Chirality at C64         (Sohnke SpGr)          R Verify
PLAT791_ALERT_4_G Model has Chirality at C65         (Sohnke SpGr)          R Verify
PLAT791_ALERT_4_G Model has Chirality at C66         (Sohnke SpGr)          R Verify
PLAT791_ALERT_4_G Model has Chirality at C68         (Sohnke SpGr)          R Verify
PLAT791_ALERT_4_G Model has Chirality at C69         (Sohnke SpGr)          S Verify
PLAT791_ALERT_4_G Model has Chirality at C70         (Sohnke SpGr)          R Verify
PLAT791_ALERT_4_G Model has Chirality at C71         (Sohnke SpGr)          R Verify
PLAT791_ALERT_4_G Model has Chirality at C72         (Sohnke SpGr)          R Verify
```

```
PLAT791_ALERT_4_G Model has Chirality at C74        (Sohnke SpGr)          R Verify
PLAT791_ALERT_4_G Model has Chirality at C75        (Sohnke SpGr)          S Verify
PLAT791_ALERT_4_G Model has Chirality at C76        (Sohnke SpGr)          R Verify
PLAT791_ALERT_4_G Model has Chirality at C77        (Sohnke SpGr)          R Verify
PLAT791_ALERT_4_G Model has Chirality at C78        (Sohnke SpGr)          R Verify
PLAT791_ALERT_4_G Model has Chirality at C80        (Sohnke SpGr)          R Verify
PLAT791_ALERT_4_G Model has Chirality at C81        (Sohnke SpGr)          S Verify
PLAT791_ALERT_4_G Model has Chirality at C82        (Sohnke SpGr)          R Verify
PLAT791_ALERT_4_G Model has Chirality at C83        (Sohnke SpGr)          R Verify
PLAT791_ALERT_4_G Model has Chirality at C84        (Sohnke SpGr)          R Verify
PLAT860_ALERT_3_G Number of Least-Squares Restraints .............          4 Note
PLAT869_ALERT_4_G ALERTS Related to the Use of SQUEEZE Suppressed           ! Info
PLAT883_ALERT_1_G No Info/Value for _atom_sites_solution_primary .     Please Do !
PLAT941_ALERT_3_G Average HKL Measurement Multiplicity ...........        3.1 Low
PLAT965_ALERT_2_G The SHELXL WEIGHT Optimisation has not Converged     Please Check
PLAT984_ALERT_1_G The  C-f'=    0.0150 Deviates from the B&C-Value     0.0137 Check
PLAT984_ALERT_1_G The  O-f'=    0.0410 Deviates from the B&C-Value     0.0389 Check
```

```
   0 ALERT level A = Most likely a serious problem - resolve or explain
  26 ALERT level B = A potentially serious problem, consider carefully
  13 ALERT level C = Check. Ensure it is not caused by an omission or oversight
  87 ALERT level G = General information/check it is not something unexpected

  10 ALERT type 1 CIF construction/syntax error, inconsistent or missing data
  34 ALERT type 2 Indicator that the structure model may be wrong or deficient
   5 ALERT type 3 Indicator that the structure quality may be low
  76 ALERT type 4 Improvement, methodology, query or suggestion
   1 ALERT type 5 Informative message, check
```

---

checkCIF publication errors

Alert level A

```
PUBL004_ALERT_1_A  The contact author's name and address are missing,
            _publ_contact_author_name and _publ_contact_author_address.
PUBL005_ALERT_1_A  _publ_contact_author_email, _publ_contact_author_fax and
            _publ_contact_author_phone are all missing.
            At least one of these should be present.
PUBL006_ALERT_1_A  _publ_requested_journal is missing
            e.g. 'Acta Crystallographica Section C'
PUBL008_ALERT_1_A  _publ_section_title is missing. Title of paper.
PUBL009_ALERT_1_A  _publ_author_name is missing. List of author(s) name(s).
PUBL010_ALERT_1_A  _publ_author_address is missing. Author(s) address(es).
PUBL012_ALERT_1_A  _publ_section_abstract is missing.
            Abstract of paper in English.
```

Alert level G

```
PUBL017_ALERT_1_G The _publ_section_references section is missing or
            empty.
```

```
   7 ALERT level A = Data missing that is essential or data in wrong format
   1 ALERT level G = General alerts. Data that may be required is missing
```

Publication of your CIF

You should attempt to resolve as many as possible of the alerts in all categories. Often the minor alerts point to easily fixed oversights, errors and omissions in your CIF or refinement strategy, so attention to these fine details can be worthwhile. In order to resolve some of the more serious problems it may be necessary to carry out additional measurements or structure refinements. However, the nature of your study may justify the reported deviations from journal submission requirements and the more serious of these should be commented upon in the discussion or experimental section of a paper or in the "special_details" fields of the CIF. *checkCIF* was carefully designed to identify outliers and unusual parameters, but every test has its limitations and alerts that are not important in a particular case may appear. Conversely, the absence of alerts does not guarantee there are no aspects of the results needing attention. It is up to the individual to critically assess their own results and, if necessary, seek expert advice.

If level A alerts remain, which you believe to be justified deviations, and you intend to submit this CIF for publication in a journal, you should additionally insert an explanation in your CIF using the Validation Reply Form (VRF) below. This will allow your explanation to be considered as part of the review process.

Validation response form

Please find below a validation response form (VRF) that can be filled in and pasted into your CIF.

```
# start Validation Reply Form
_vrf_PUBL004_GLOBAL
;
PROBLEM: The contact author’s name and address are missing,
RESPONSE: ...
;
_vrf_PUBL005_GLOBAL
;
PROBLEM: _publ_contact_author_email, _publ_contact_author_fax and
RESPONSE: ...
;
_vrf_PUBL006_GLOBAL
;
PROBLEM: _publ_requested_journal is missing
RESPONSE: ...
;
_vrf_PUBL008_GLOBAL
;
PROBLEM: _publ_section_title is missing. Title of paper.
RESPONSE: ...
;
_vrf_PUBL009_GLOBAL
;
PROBLEM: _publ_author_name is missing. List of author(s) name(s).
RESPONSE: ...
;
_vrf_PUBL010_GLOBAL
;
PROBLEM: _publ_author_address is missing. Author(s) address(es).
RESPONSE: ...
;
_vrf_PUBL012_GLOBAL
;
```

```
PROBLEM: _publ_section_abstract is missing.
RESPONSE: ...
;
# end Validation Reply Form
```

If you wish to submit your CIF for publication in Acta Crystallographica Section C or E, you should upload your CIF via the web. If you wish to submit your CIF for publication in IUCrData you should upload your CIF via the web. If your CIF is to form part of a submission to another IUCr journal, you will be asked, either during electronic submission or by the Co-editor handling your paper, to upload your CIF via our web site.

PLATON version of 22/03/2021; check.def file version of 19/03/2021

Datablock I - ellipsoid plot

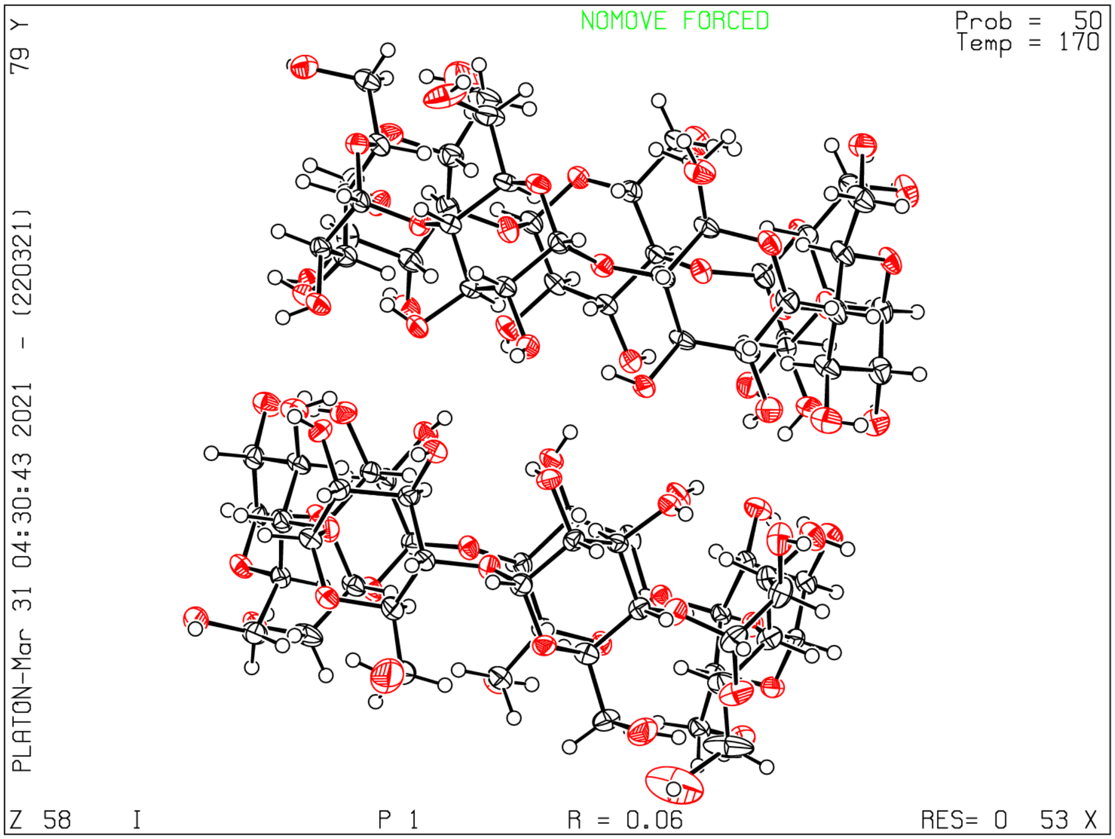

Supplement: Supplementary file 1 [file molecules-26-02412-s001.zip › CIF and CheckCIF/CheckCIF_Complex 6.pdf]
