# Supplementary material for: Structural Insights into the Host–Guest Complexation between β-Cyclodextrin and Bio-Conjugatable Adamantane Derivatives
Source: Molecules. 2021 Apr 21;26(9):2412. doi: 10.3390/molecules26092412 (PMC8122645; doi:10.3390/molecules26092412)
Supplement: Supplementary file 1 [file molecules-26-02412-s001.zip › Supplmentary Material for XML.pdf]

molecules

MDPI

Supplementary Material

Structural Insights into the Host-Guest Complexation between *β*-Cyclodextrin and Bio-conjugatable Adamantane Derivatives

Table of Contents

Figure S1. The ^{1}H NMR spectrum (500 MHz, DMSO-d_6, room temperature) of 1. 3

Figure S2. The ^{1}H NMR spectrum (500 MHz, DMSO-d_6, room temperature) of 2. 3

Figure S3. The ^{1}H NMR spectrum (500 MHz, DMSO-d_6, room temperature) of 3. 4

Figure S4. The ^{1}H NMR spectrum (500 MHz, DMSO-d_6, room temperature) of 4. 4

Figure S5. The ^{1}H NMR spectrum (500 MHz, DMSO-d_6, room temperature) of 5. 5

Figure S6. The ^{1}H NMR spectrum (500 MHz, DMSO-d_6, room temperature) of 6. 5

Figure S7. The ^{1}H NMR spectra (500 MHz, DMSO-d_6, room temperature) of 1, β-CD and adm-1-OH. 6

Figure S8. The ^{1}H NMR spectra (500 MHz, DMSO-d_6, room temperature) of 2, β-CD and adm-2-OH. 6

Figure S9. The ^{1}H NMR spectra (500 MHz, DMSO-d_6, room temperature) of 3, β-CD and adm-1-NH_2. 7

Figure S10. The ^{1}H NMR spectra (500 MHz, DMSO-d_6, room temperature) of 4, β-CD and adm-1-COOH. 7

Figure S11. The ^{1}H NMR spectra (500 MHz, DMSO-d_6, room temperature) of 5, β-CD and adm-1,3-diCOOH. 8

Figure S12. The ^{1}H NMR spectra (500 MHz, DMSO-d_6, room temperature) of 6, β-CD and adm-1,3-diCH_2COOH. 8

Figure S13. (a) 2D NOESY spectra (500 MHz, DMSO-d_6, room temperature) of 1 with (b) expansion of significant regions showing interactions between adm-1-OH and β-CD. 9

Figure S14. (a) 2D NOESY spectra (500 MHz, DMSO-d_6, room temperature) of 2 with (b) expansion of significant regions showing interactions between adm-2-OH and β-CD. 9

Figure S15. (a) 2D NOESY spectra (500 MHz, DMSO-d_6, room temperature) of 3 with (b) expansion of significant regions showing interactions between adm-1-NH_2 and β-CD. 9

Figure S16. (a) 2D NOESY spectra (500 MHz, DMSO-d_6, room temperature) of 4 with (b) expansion of significant regions showing interactions between adm-1-NH_2 and β-CD. 10

Figure S17. (a) 2D NOESY spectra (500 MHz, DMSO-d_6, room temperature) of 5 with (b) expansion of significant regions showing interactions between adm-1,3-diCOOH and β-CD. 10

Figure S18. (a) 2D NOESY spectra (500 MHz, DMSO-d_6, room temperature) of 6 with (b) expansion of significant regions showing interactions between adm-1,3-diCH_2COOH and β-CD. 10

Figure S19. (a) TG traces of adm-2-OH, β-CD and 2. (b) A comparison of the DSC-TGA curves of 2 and its subcomponents β-CD and adm-2-OH, showing the melting point alteration upon complex formation. 11

Figure S20. (a) TG traces of adm-1-NH_2, β-CD and 3. (b) A comparison of the DSC-TGA curves of 3 and its subcomponents β-CD and adm-1-NH_2, showing the melting point alteration upon complex formation. 11

Figure S21. (a) TG traces of adm-1-COOH, β-CD and 4. (b) A comparison of the DSC-TGA curves of 4 and its subcomponents β-CD and adm-1-COOH, showing the melting point alteration upon complex formation. 11

Figure S22. (a) TG traces of adm-1,3-diCOOH, β-CD and 5. (b) A comparison of the DSC-TGA curves of 5 and its subcomponents β-CD and adm-1,3-diCOOH, showing the melting point alteration upon complex formation. 12

Figure S23. (a) TG traces of adm-1,3-diCH_2COOH, β-CD and 6. (b) A comparison of the DSC-TGA curves of 6 its subcomponents β-CD and adm-1,3-diCH_2COOH, showing the melting point alteration upon complex formation. 12

Figure S24. The X-ray crystal structure of 3 showing the diverse guest orientations in the inclusion complexes. The guests are presented as a space-filling model. The hydrogen atoms are omitted for clarity. Color codes: O (brown-red), N (blue), C (black). ...13

Figure S25. The crystal packing diagram of 6 (along *c* direction). Color codes: O (red) and C (gray). 13

Table S1. Chemical shifts of adamantane derivative 1 with/without complexation with β-CD (recorded in DMSO-d_6 with TMS as the internal standard) for comparison. 14

Table S2. Chemical shifts of adamantane derivative 2 with/without complexation with β-CD (recorded in DMSO-d_6 with TMS as the internal standard) for comparison. 14

Table S3. Chemical shifts of adamantane derivative 3 with/without complexation with β-CD (recorded in DMSO-d_6 with TMS as the internal standard) for comparison. 14

Table S4. Chemical shifts of adamantane derivative 4 with/without complexation with β-CD (recorded in DMSO-d_6 with TMS as the internal standard) for comparison. 14

Table S5. Chemical shifts of adamantane derivative 5 with/without complexation with β-CD (recorded in DMSO-d_6 with TMS as the internal standard) for comparison. 14

Table S6. Chemical shifts of adamantane derivative 6 with/without complexation with β-CD (recorded in DMSO-d_6 with TMS as the internal standard) for comparison. 15

Table S7. Chemical shifts of β-CD in 1 with/without complexation with adamantane derivative (recorded in DMSO-d_6 with TMS as the internal standard) for comparison. 15

Table S8. Chemical shifts of β-CD in 2 with/without complexation with adamantane derivative (recorded in DMSO-d_6 with TMS as the internal standard) for comparison. 15

Table S9. Chemical shifts of β-CD in 3 with/without complexation with adamantane derivative (recorded in DMSO-d_6 with TMS as the internal standard) for comparison. 16

Table S10. Chemical shifts of β-CD in 4 with/without complexation with adamantane derivative (recorded in DMSO-d_6 with TMS as the internal standard) for comparison. 16

Table S11. Chemical shifts of β-CD in 5 with/without complexation with adamantane derivative (recorded in DMSO-d_6 with TMS as the internal standard) for comparison. 16

Table S12. Chemical shifts of β-CD in 6 with/without complexation with adamantane derivative (recorded in DMSO-d_6 with TMS as the internal standard) for comparison. 17

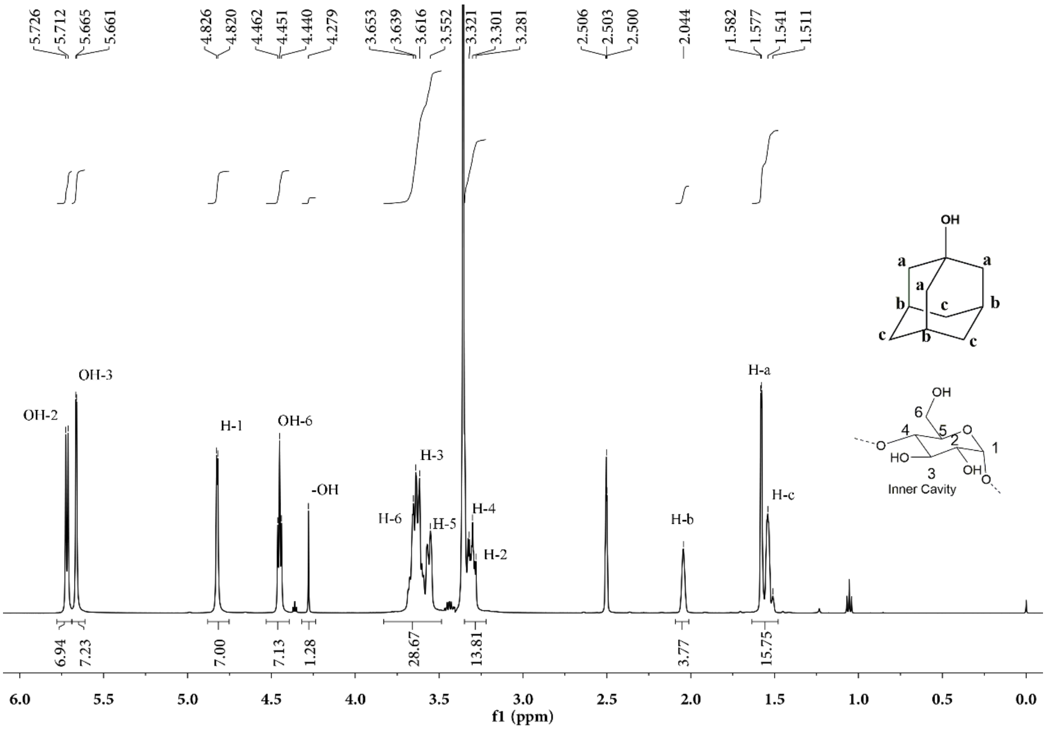

Figure S1. The ^{1}H NMR spectrum (500 MHz, DMSO-d_6, room temperature) of **1**.

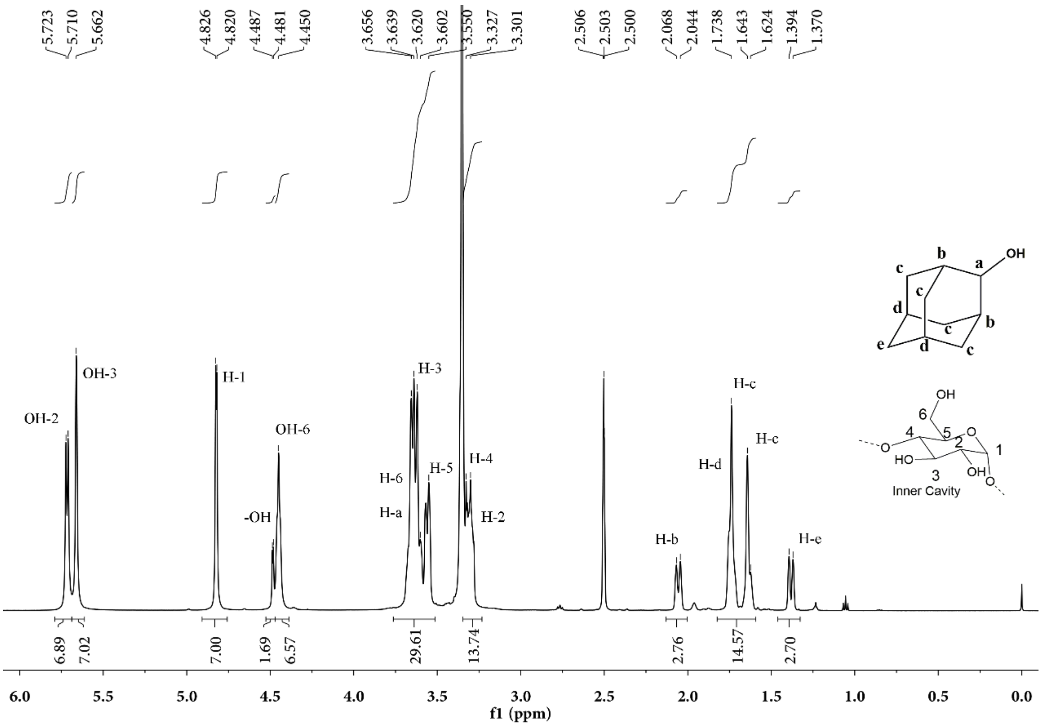

Figure S2. The ^{1}H NMR spectrum (500 MHz, DMSO-d_6, room temperature) of **2.**

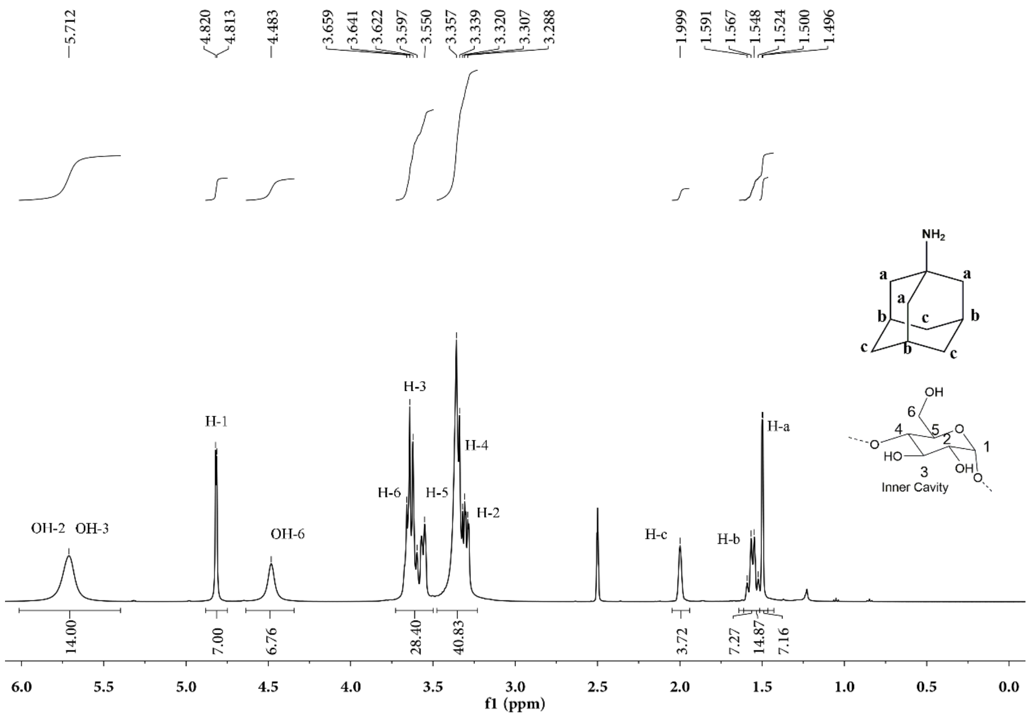

Figure S3. The ^{1}H NMR spectrum (500 MHz, DMSO-d_6, room temperature) of **3.**

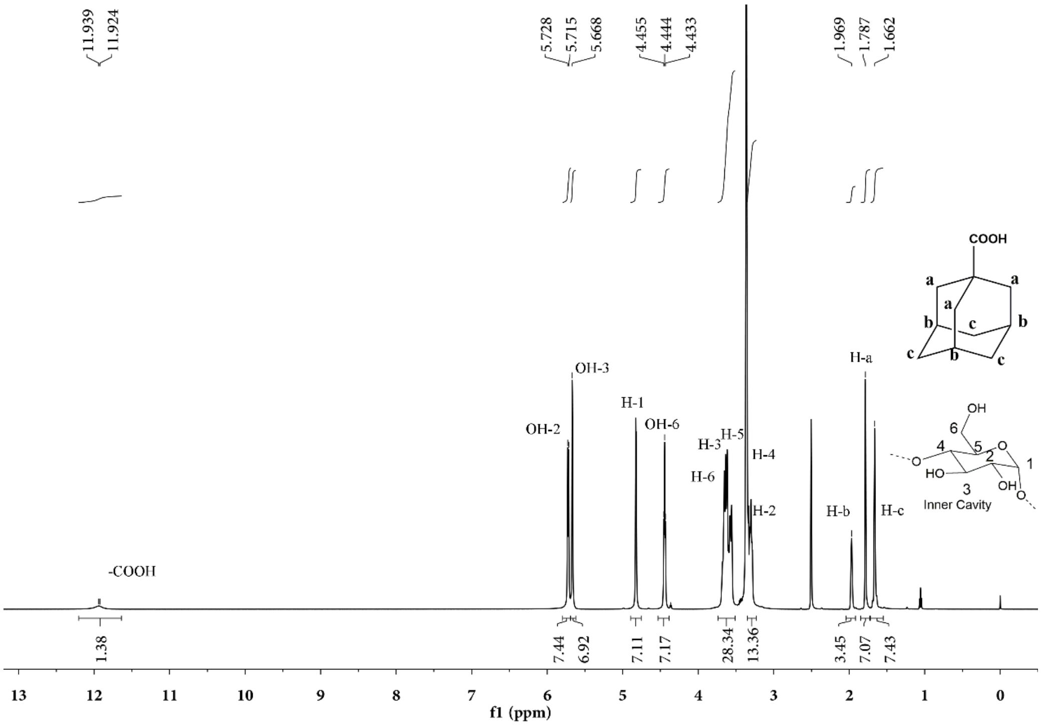

Figure S4. The ^{1}H NMR spectrum (500 MHz, DMSO-d_6, room temperature) of **4.**

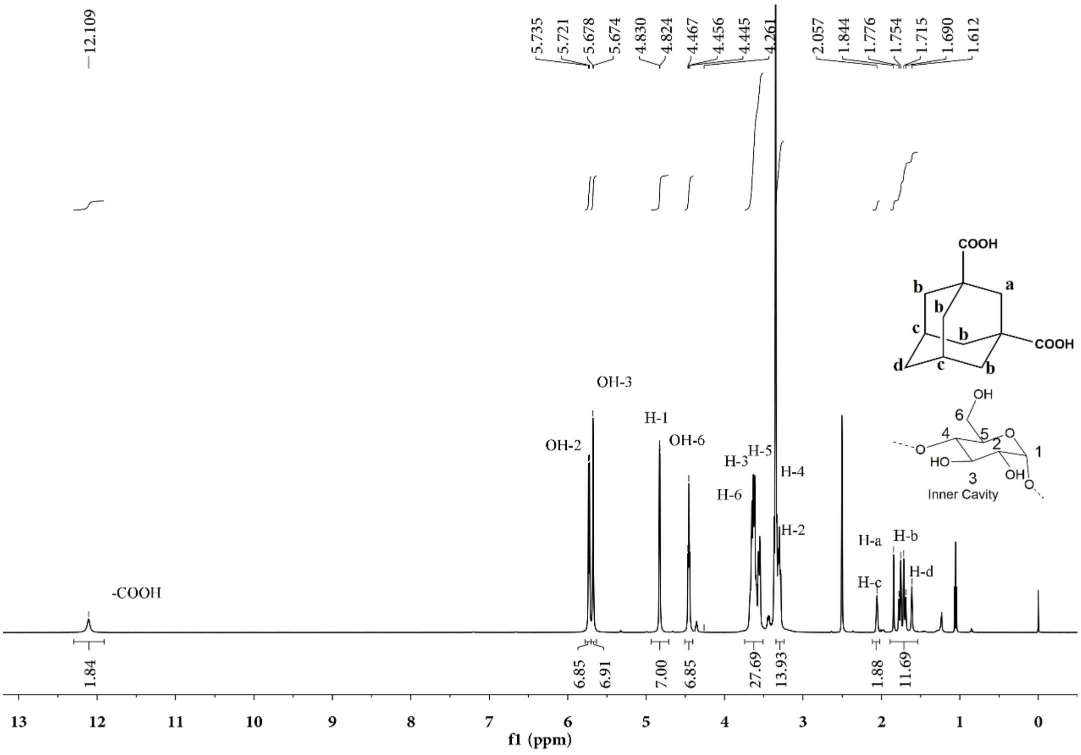

Figure S5. The ^{1}H NMR spectrum (500 MHz, DMSO-d_6, room temperature) of **5.**

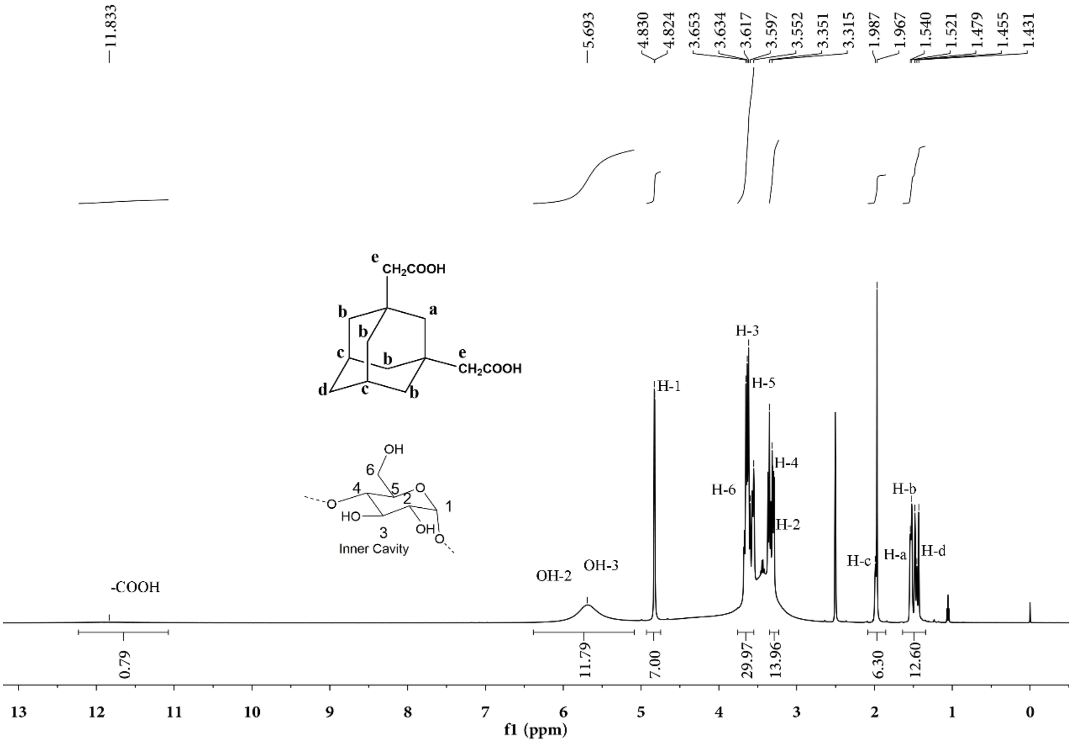

Figure S6. The ^{1}H NMR spectrum (500 MHz, DMSO-d_6, room temperature) of **6.**

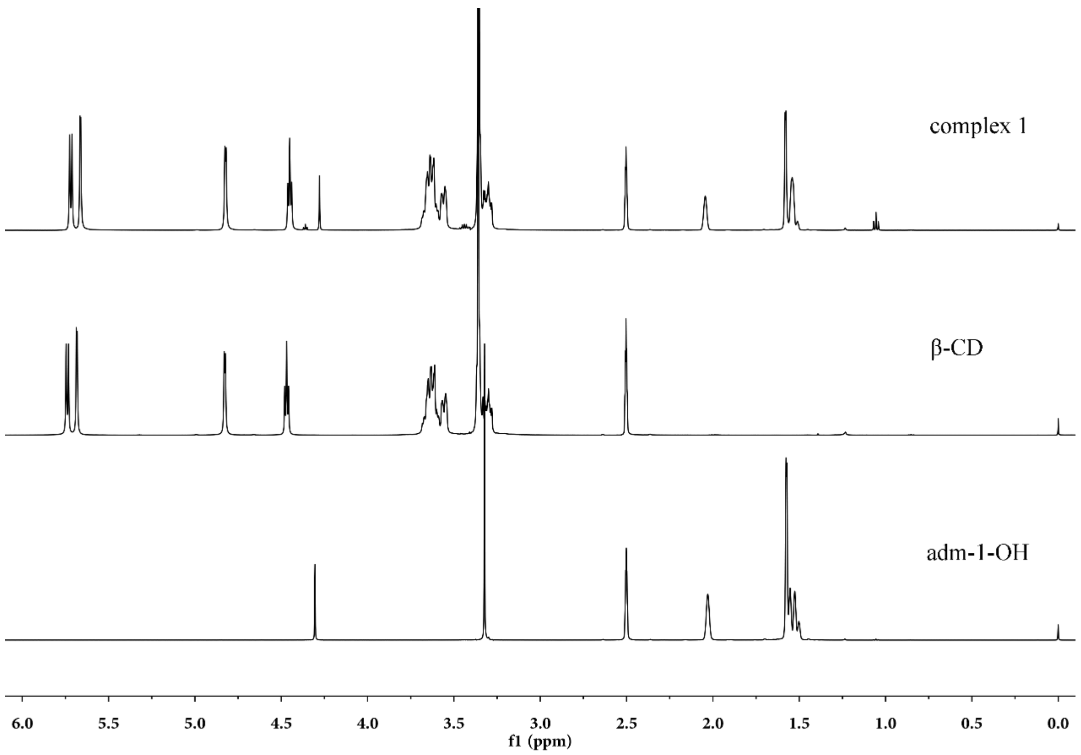

Figure S7. The ^{1}H NMR spectra (500 MHz, DMSO-d_6, room temperature) of **1**, β-CD and adm-1-OH.

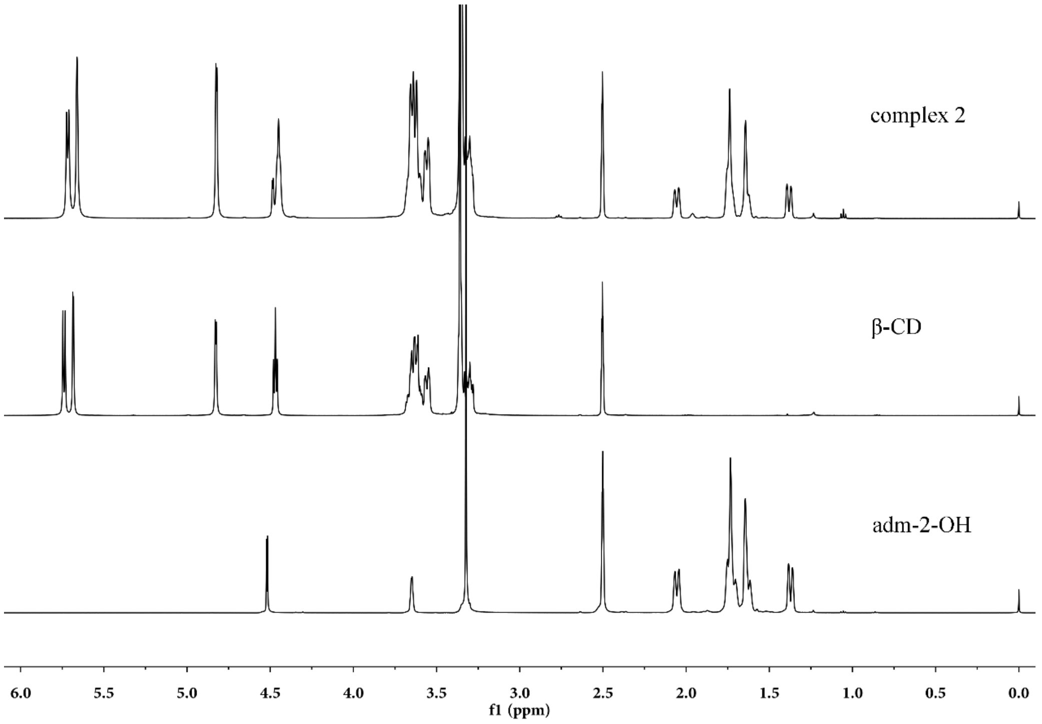

Figure S8. The ^{1}H NMR spectra (500 MHz, DMSO-d_6, room temperature) of **2**, β-CD and adm-2-OH.

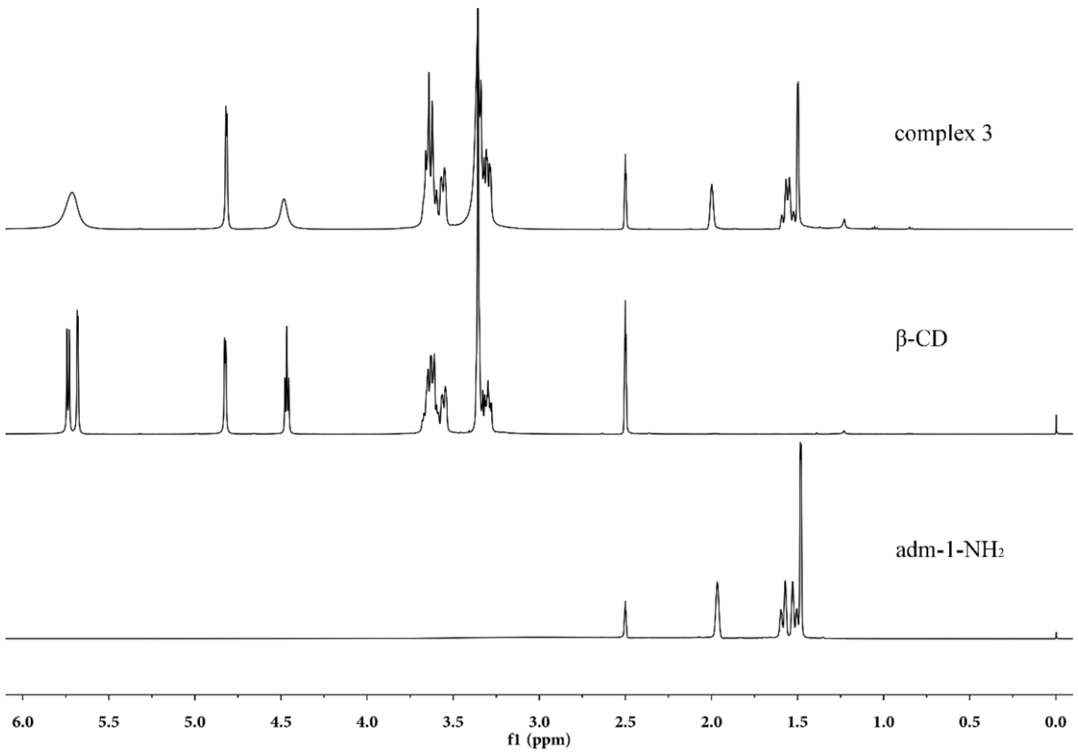

Figure S9. The ^{1}H NMR spectra (500 MHz, DMSO-d_6, room temperature) of **3**, β-CD and adm-1-NH_2.

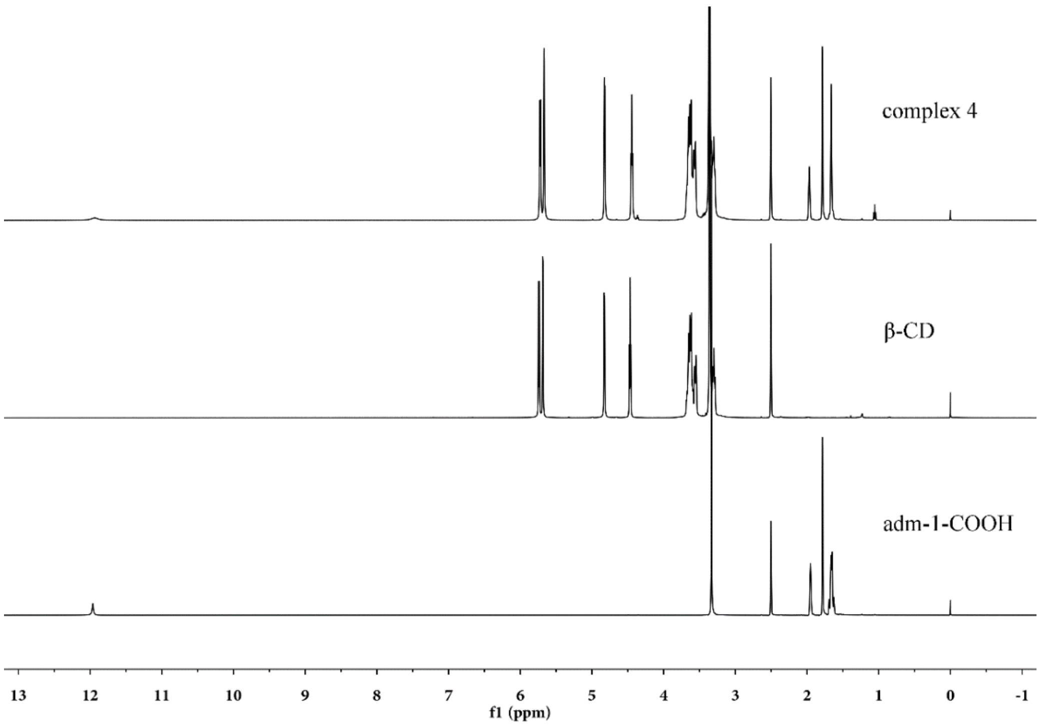

Figure S10. The ^{1}H NMR spectra (500 MHz, DMSO-d_6, room temperature) of **4**, β-CD and adm-1-COOH.

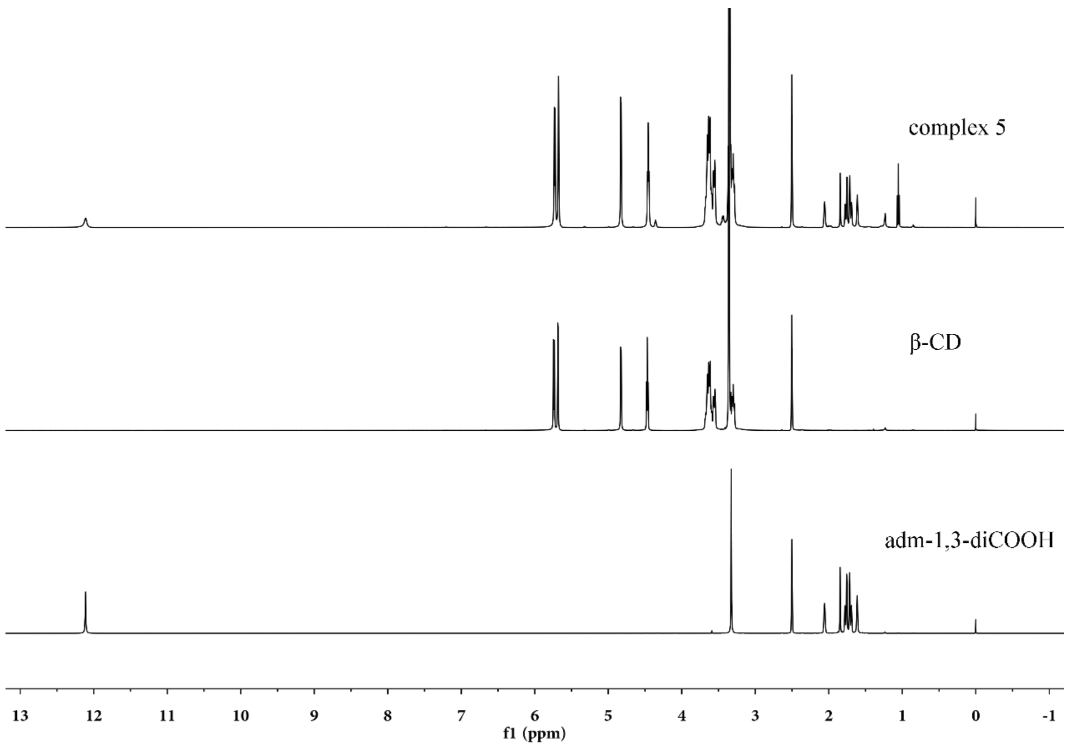

Figure S11. The ^{1}H NMR spectra (500 MHz, DMSO-d_6, room temperature) of **5**, β-CD and adm-1,3-diCOOH.

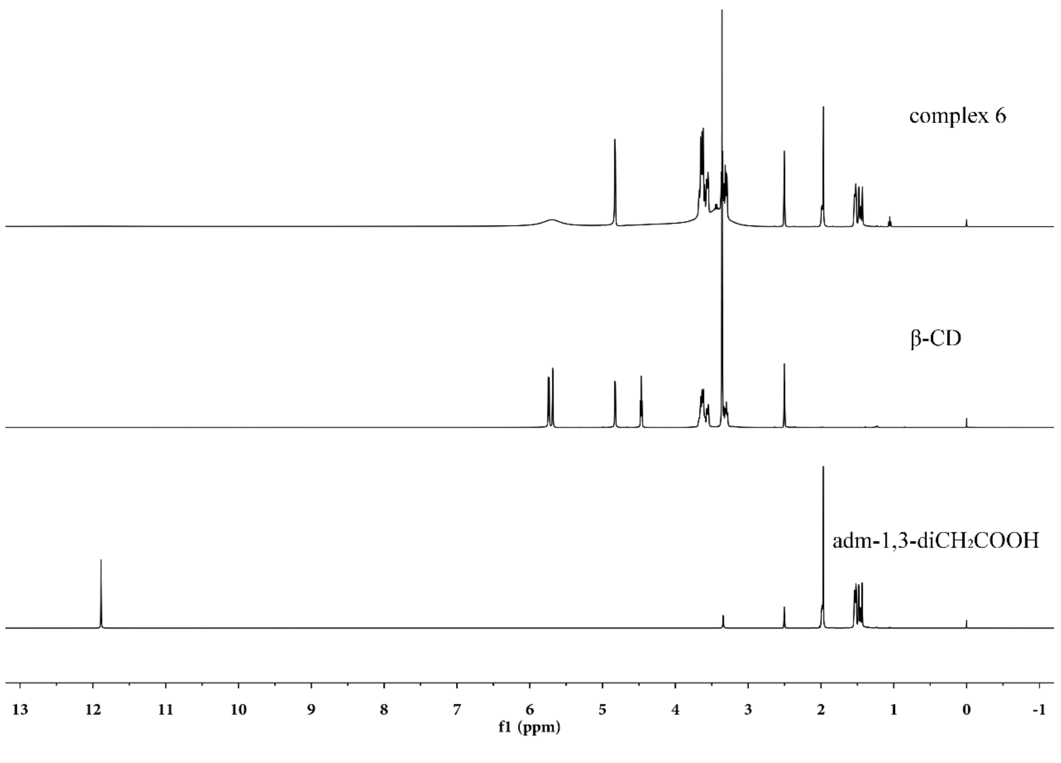

Figure S12. The ^{1}H NMR spectra (500 MHz, DMSO-d_6, room temperature) of **6**, β-CD and adm-1,3-diCH_2COOH.

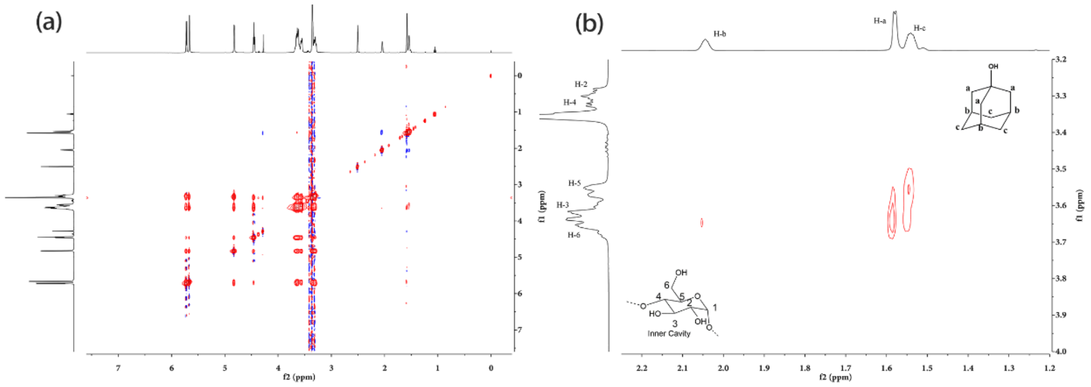

Figure S13. (**a**) 2D NOESY spectra (500 MHz, DMSO-d_6, room temperature) of **1** with (**b**) expansion of significant regions showing interactions between adm-1-OH and β-CD.

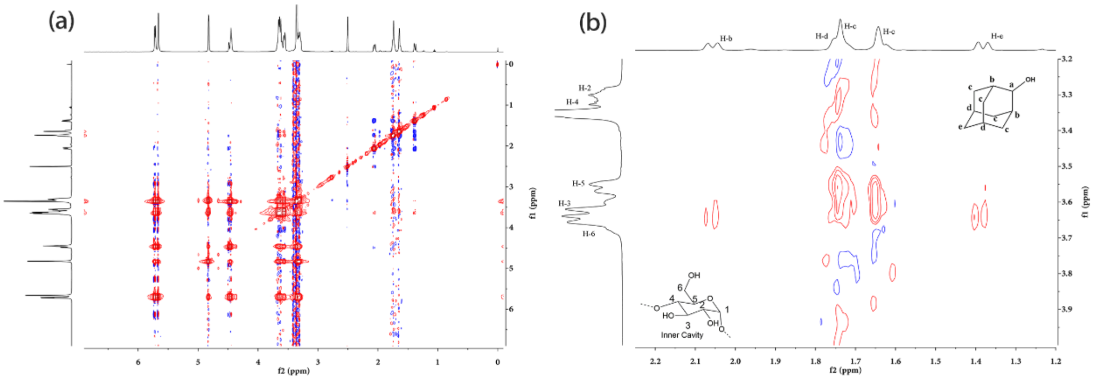

Figure S14. (**a**) 2D NOESY spectra (500 MHz, DMSO-d_6, room temperature) of **2** with (**b**) expansion of significant regions showing interactions between adm-2-OH and β-CD.

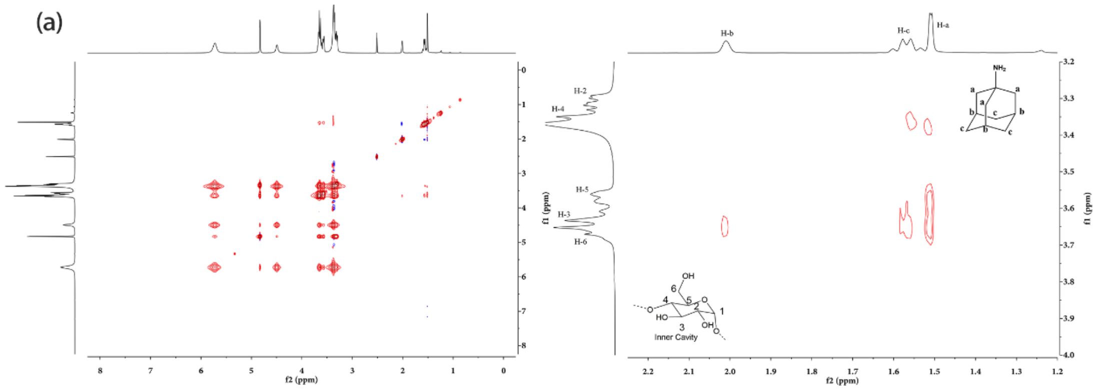

Figure S15. (**a**) 2D NOESY spectra (500 MHz, DMSO-d_6, room temperature) of **3** with (**b**) expansion of significant regions showing interactions between adm-1-NH_2 and β-CD.

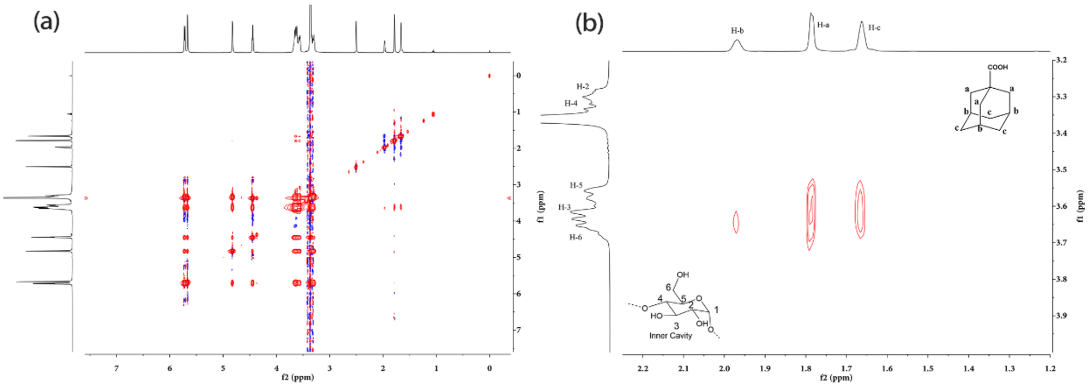

Figure S16. (**a**) 2D NOESY spectra (500 MHz, DMSO-d_6, room temperature) of **4** with (**b**) expansion of significant regions showing interactions between adm-1-NH_2 and β-CD.

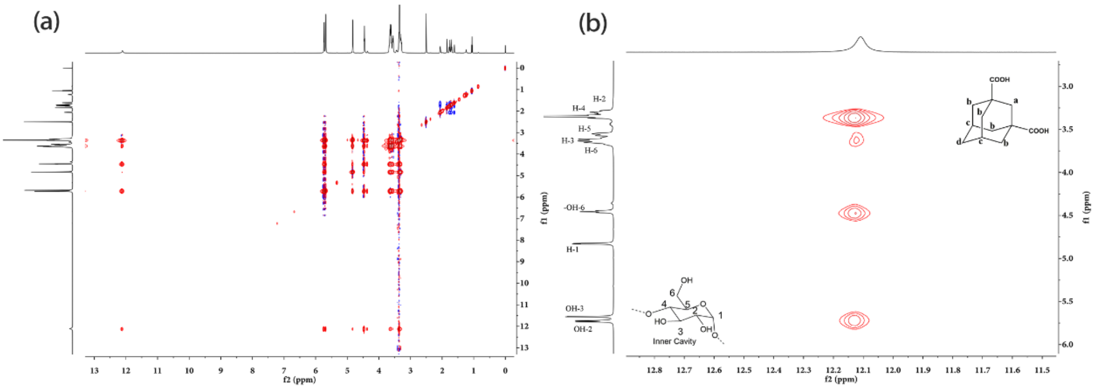

Figure S17. (**a**) 2D NOESY spectra (500 MHz, DMSO-d_6, room temperature) of **5** with (**b**) expansion of significant regions showing interactions between adm-1,3-diCOOH and β-CD.

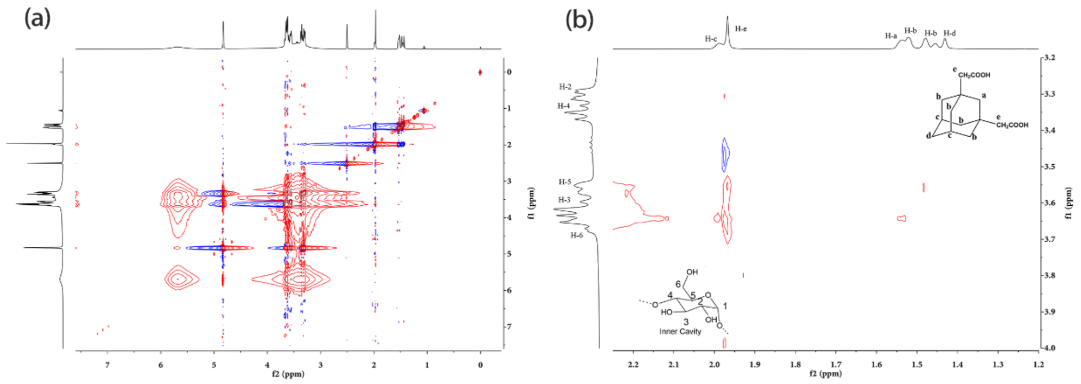

Figure S18. (**a**) 2D NOESY spectra (500 MHz, DMSO-d_6, room temperature) of **6** with (**b**) expansion of significant regions showing interactions between adm-1,3-diCH_2COOH and β-CD.

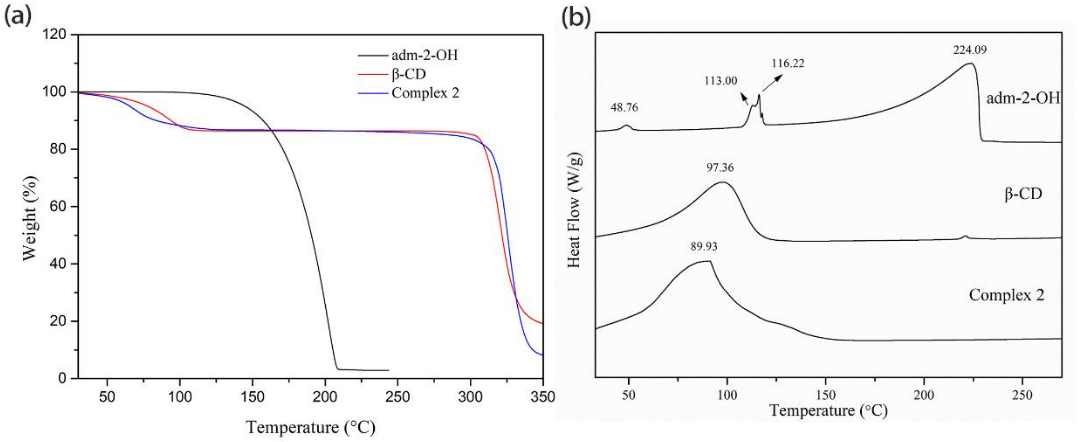

Figure S19. (**a**) TG traces of adm-2-OH, β-CD and **2**. (**b**) A comparison of the DSC-TGA curves of 2 and its subcomponents β-CD and adm-2-OH, showing the melting point alteration upon complex formation.

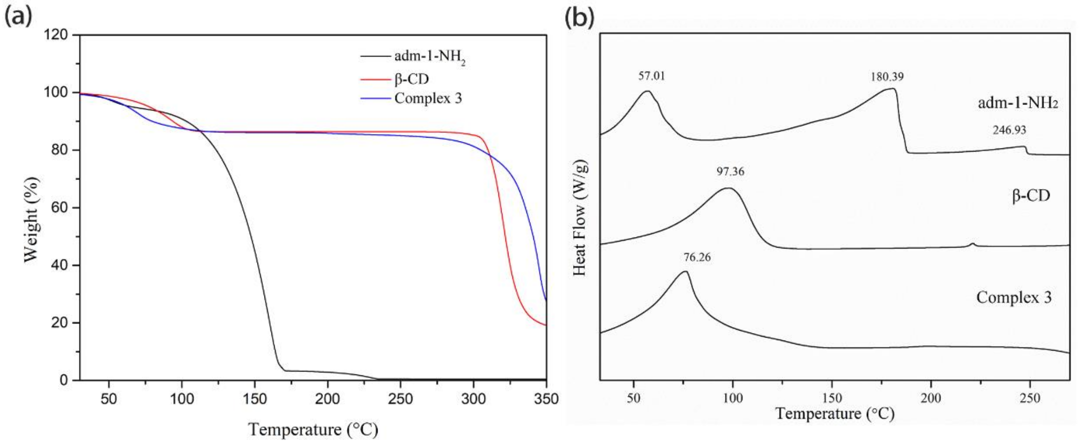

Figure S20. (**a**) TG traces of adm-1-NH_2, β-CD and **3**. (**b**) A comparison of the DSC-TGA curves of 3 and its subcomponents β-CD and adm-1-NH_2, showing the melting point alteration upon complex formation.

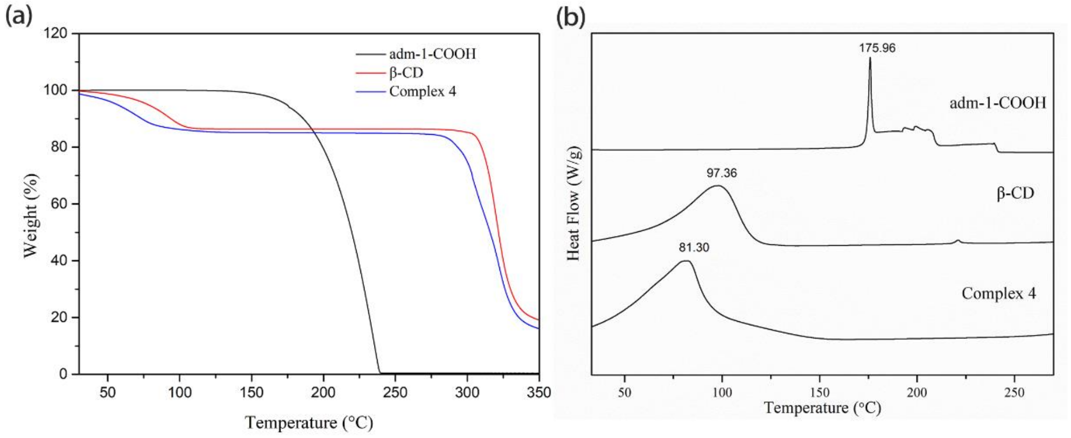

Figure S21. (**a**) TG traces of adm-1-COOH, β-CD and **4**. (**b**) A comparison of the DSC-TGA curves of 4 and its subcomponents β-CD and adm-1-COOH, showing the melting point alteration upon complex formation.

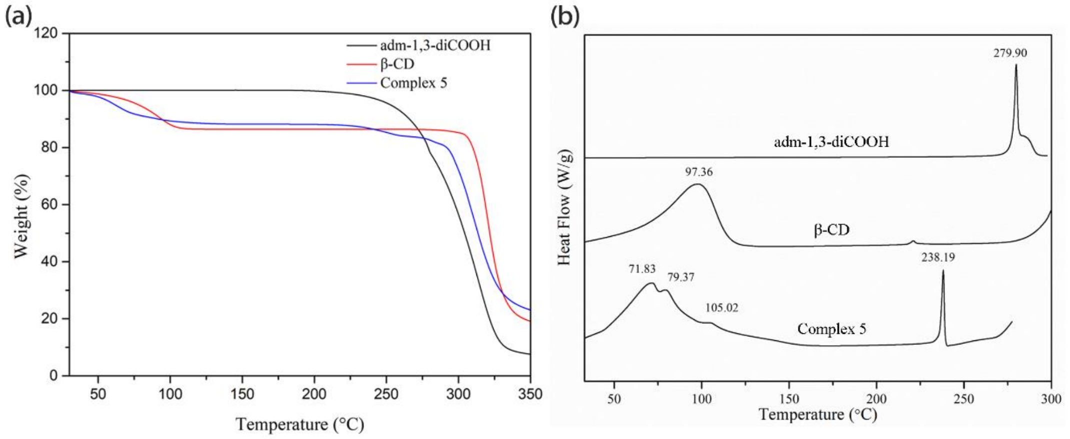

Figure S22. (**a**) TG traces of adm-1,3-diCOOH, β-CD and **5**. (**b**) A comparison of the DSC-TGA curves of 5 and its sub-components β-CD and adm-1,3-diCOOH, showing the melting point alteration upon complex formation.

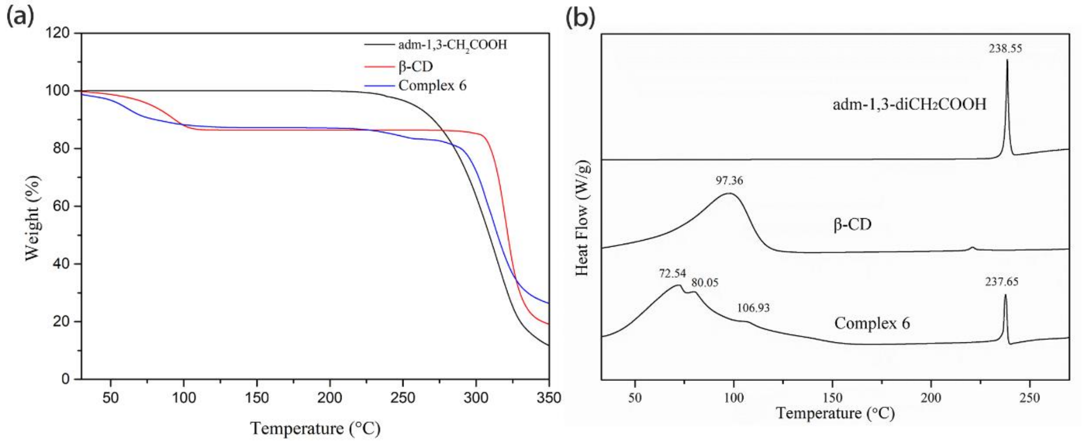

Figure S23. (**a**) TG traces of adm-1,3-diCH2COOH, β-CD and **6**. (**b**) A comparison of the DSC-TGA curves of 6 its sub-components β-CD and adm-1,3-diCH2COOH, showing the melting point alteration upon complex formation.

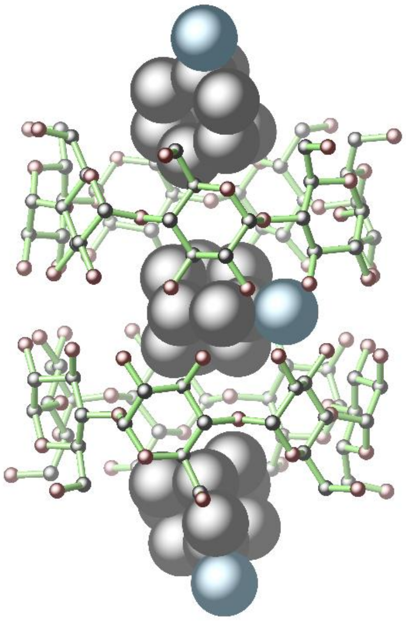

Figure S24. The X-ray crystal structure of **3** showing the diverse guest orientations in the inclusion complexes. The guests are presented as a space-filling model. The hydrogen atoms are omitted for clarity. Color codes: O (brown-red), N (blue), C (black).

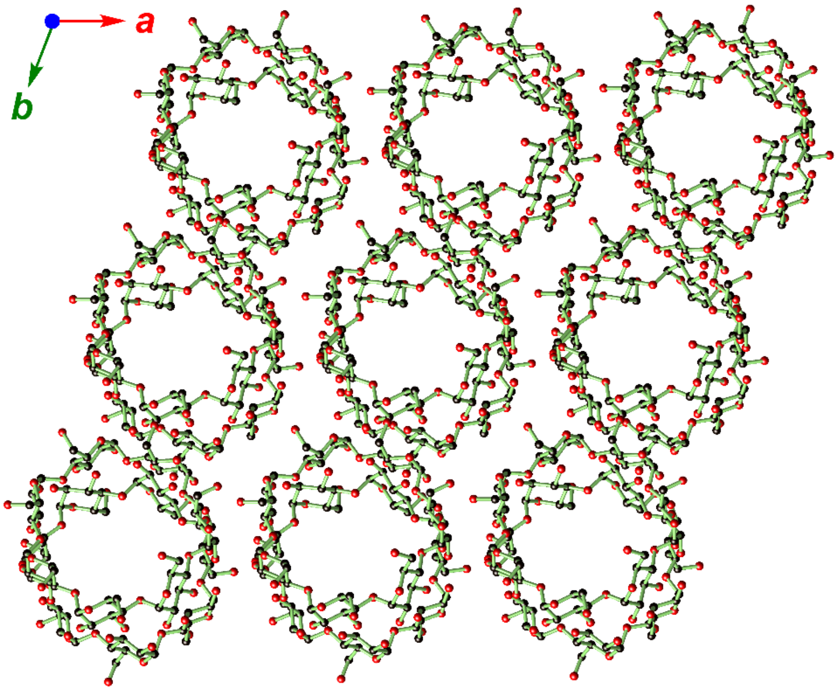

Figure S25. The crystal packing diagram of 6 (along *c* direction). Color codes: O (red) and C (gray).

Table S1. Chemical shifts of adamantane derivative **1** with/without complexation with β-CD (recorded in DMSO-d_6 with TMS as the internal standard) for comparison.

Adamantane Derivative 1	Hydrogen	$\delta_{free\ state}$	$\delta_{complexed\ state}$	$\Delta\delta^a$	Remark
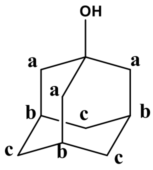	H-a	1.5739	1.5796	0.0057	
	H-b	2.0296	2.0442	0.0146	
	H-c	1.5394	1.5406	0.0012	no split
	-OH	4.3049	4.2786	−0.0263	

a. $\Delta\delta = \delta_{complexed\ state} - \delta_{free\ state}$.

Table S2. Chemical shifts of adamantane derivative **2** with/without complexation with β-CD (recorded in DMSO-d_6 with TMS as the internal standard) for comparison.

Adamantane Derivative 2	Hydrogen	$\delta_{free\ state}$	$\delta_{complexed\ state}$	$\Delta\delta^a$	Remark
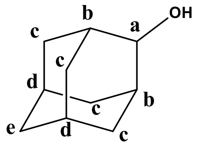	H-a	3.6495	3.6019	−0.0476	
	H-b	2.0549	2.0561	0.0012	
	H-c	1.6889	1.6905	0.0016	
	H-d	1.7029	Merge with peak of 1.7381	0.0352	
	H-e	1.3723	1.3818	0.0095	
	-OH	4.5192	4.4843	−0.0349	

a. $\Delta\delta = \delta_{complexed\ state} - \delta_{free\ state}$.

Table S3. Chemical shifts of adamantane derivative **3** with/without complexation with β-CD (recorded in DMSO-d_6 with TMS as the internal standard) for comparison.

Adamantane Derivative 3	Hydrogen	$\delta_{free\ state}$	$\delta_{complexed\ state}$	$\Delta\delta^a$	Remark
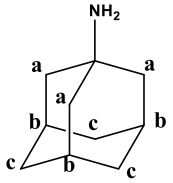	H-a	1.4813	1.4983	0.017	
	H-b	1.9655	1.9987	0.0332	
	H-c	1.5492	1.5573	0.0081	
	-NH_2	broad	disappear		

a. $\Delta\delta = \delta_{complexed\ state} - \delta_{free\ state}$.

Table S4. Chemical shifts of adamantane derivative **4** with/without complexation with β-CD (recorded in DMSO-d_6 with TMS as the internal standard) for comparison.

Adamantane Derivative 4	Hydrogen	$\delta_{free\ state}$	$\delta_{complexed\ state}$	$\Delta\delta^a$	Remark
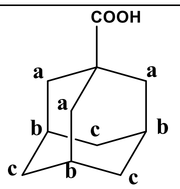	H-a	1.7836	1.7874	0.0038	
	H-b	1.9526	1.9688	0.0162	
	H-c	1.6588	1.6624	0.0036	no split
	-COOH	11.9616	11.9389	−0.0227	

a. $\Delta\delta = \delta_{complexed\ state} - \delta_{free\ state}$.

Table S5. Chemical shifts of adamantane derivative **5** with/without complexation with β-CD (recorded in DMSO-d_6 with TMS as the internal standard) for comparison.

Adamantane Derivative 5	Hydrogen	$\delta_{free\ state}$	$\delta_{complexed\ state}$	$\Delta\delta^a$	Remark
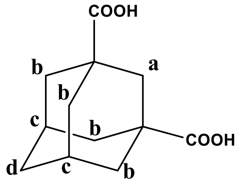	H-a	1.8453	1.8439	−0.0014	
	H-b	1.7361	1.7343	−0.0018	
	H-c	2.0573	2.0570	−0.0003	
	H-d	1.6132	1.6119	−0.0013	
	-COOH	12.1117	12.1087	−0.0030	

a. $\Delta\delta = \delta_{\text{complexed state}} - \delta_{\text{free state}}$.

Table S6. Chemical shifts of adamantane derivative **6** with/without complexation with β-CD (recorded in DMSO-d_6 with TMS as the internal standard) for comparison.

Adamantane Derivative 6	Hydrogen	$\delta_{\text{free state}}$	$\delta_{\text{complexed state}}$	$\Delta\delta^a$	Remark
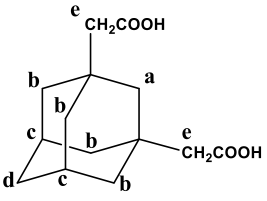	H-a	1.5392	1.5396	0.0004	Merge with peak H-b
	H-b	1.5011	1.5003	−0.0008	
	H-c	1.9866	1.9873	0.0007	
	H-d	1.4352	1.4311	−0.0041	
	H-e	1.9692	1.9674	−0.0018	
	-COOH	11.8876	11.8325	−0.0551	

a. $\Delta\delta = \delta_{\text{complexed state}} - \delta_{\text{free state}}$

Table S7. Chemical shifts of β-CD in **1** with/without complexation with adamantane derivative (recorded in DMSO-d_6 with TMS as the internal standard) for comparison.

Complex 1	Hydrogen	$\delta_{\text{free state}}$	$\delta_{\text{complexed state}}$	$\Delta\delta^a$	Remark
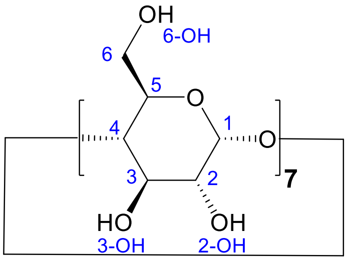	H-1	4.8270	4.8231	−0.0039	
	H-2	/	/		
	H-3	3.6117	3.6161	0.0044	
	H-4	/	/		
	H-5	3.5581	3.5614	0.0033	
	H-6	/	/		
	2-OH	5.7392	5.7189	−0.0203	
	3-OH	5.6841	5.6632	−0.0209	
	6-OH	4.4690	4.4511	−0.0179	

a. $\Delta\delta = \delta_{\text{complexed state}} - \delta_{\text{free state}}$.

Table S8. Chemical shifts of β-CD in **2** with/without complexation with adamantane derivative (recorded in DMSO-d_6 with TMS as the internal standard) for comparison.

Complex 2	Hydrogen	$\delta_{\text{free state}}$	$\delta_{\text{complexed state}}$	$\Delta\delta^a$	Remark
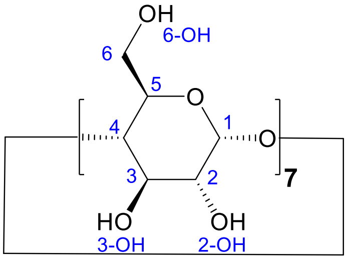	H-1	4.8270	4.8231	−0.0039	
	H-2	/	/		
	H-3	3.6117	3.6196	0.0079	
	H-4	/	/		
	H-5	3.5581	3.5597	0.0016	
	H-6	/	/		
	2-OH	5.7392	5.7165	−0.0227	
	3-OH	5.6841	5.6616	−0.0225	Doblet to siglet
	6-OH	4.4690	4.4497	−0.0193	Triplet to pro-siglet peak

a. $\Delta\delta = \delta_{\text{complexed state}} - \delta_{\text{free state}}$.

Table S9. Chemical shifts of β-CD in **3** with/without complexation with adamantane derivative (recorded in DMSO-d_6 with TMS as the internal standard) for comparison.

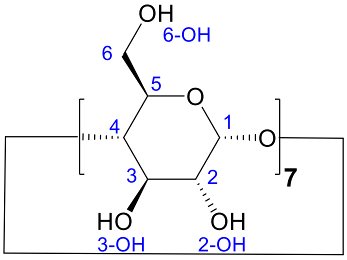

Complex 3	Hydrogen	$\delta_{free\ state}$	$\delta_{complexed\ state}$	$\Delta\delta^a$	Remark
	H-1	4.8270	4.8168	−0.0102	
	H-2	/	/		
	H-3	3.6117	3.6220	0.0103	
	H-4	/	/		
	H-5	3.5581	3.5598	0.0017	
	H-6	/	/		
	2-OH	5.7392	5.7116	−0.0276	Fused into one peak
	3-OH	5.6841	5.7116	0.0275	
	6-OH	4.4690	4.4829	0.0139	Doublet to singlet

a. $\Delta\delta = \delta_{complexed\ state} - \delta_{free\ state}$.

Table S10. Chemical shifts of β-CD in **4** with/without complexation with adamantane derivative (recorded in DMSO-d_6 with TMS as the internal standard) for comparison.

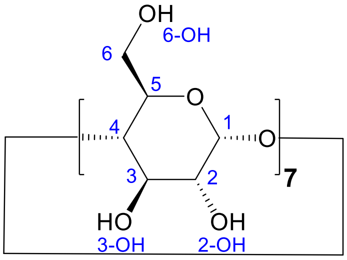

Complex 4	Hydrogen	$\delta_{free\ state}$	$\delta_{complexed\ state}$	$\Delta\delta^a$	Remark
	H-1	4.8270	4.8237	−0.0033	
	H-2	/	/		
	H-3	3.6117	3.6145	0.0028	
	H-4	/	/		
	H-5	3.5581	3.5689	0.0108	
	H-6	/	/		
	2-OH	5.7392	5.7214	−0.0178	
	3-OH	5.6841	5.6677	−0.0164	Doublet peak to singlet
	6-OH	4.4690	4.4445	−0.0245	

a. $\Delta\delta = \delta_{complexed\ state} - \delta_{free\ state}$.

Table S11. Chemical shifts of β-CD in **5** with/without complexation with adamantane derivative (recorded in DMSO-d_6 with TMS as the internal standard) for comparison.

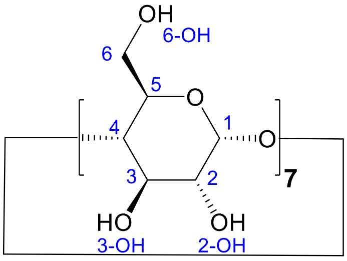

Complex 5	Hydrogen	$\delta_{free\ state}$	$\delta_{complexed\ state}$	$\Delta\delta^a$	Remark
	H-1	4.8270	4.8272	0.0002	
	H-2	/	/		
	H-3	3.6117	3.6134	0.0017	
	H-4	/	/		
	H-5	3.5581	3.5597	0.0016	
	H-6	/	/		
	2-OH	5.7392	5.7283	−0.0109	
	3-OH	5.6841	5.6759	−0.0082	No peak shape change
	6-OH	4.4690	4.4559	−0.0131	

a. $\Delta\delta = \delta_{complexed\ state} - \delta_{free\ state}$.

Table S12. Chemical shifts of β-CD in **6** with/without complexation with adamantane derivative (recorded in DMSO-d_6 with TMS as the internal standard) for comparison**.**

Complex 6	Hydrogen	$\delta_{\text{free state}}$	$\delta_{\text{complexed state}}$	$\Delta\delta^{a}$	Remark
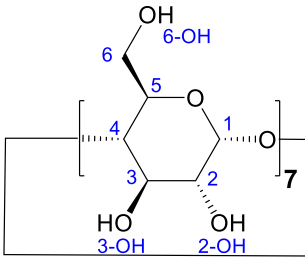	H-1	4.8270	4.8272	0.0002	
	H-2	/	/		
	H-3	3.6117	3.6168	0.0051	
	H-4	/	/		
	H-5	3.5581	3.5616	0.0035	
	H-6	/	/		
	2-OH	5.7392	5.6927	−0.0465	Fused into one peak
	3-OH	5.6841	5.6927	0.0086	
	6-OH	4.4690	vanish	0.0002	

a. $\Delta\delta = \delta_{\text{complexed state}} - \delta_{\text{free state}}$.
