# Supplementary material for: Structural Insights into the Host–Guest Complexation between β-Cyclodextrin and Bio-Conjugatable Adamantane Derivatives
Source: Molecules. 2021 Apr 21;26(9):2412. doi: 10.3390/molecules26092412 (PMC8122645; doi:10.3390/molecules26092412)
Supplement: Supplementary file 1 [file molecules-26-02412-s001.zip › Supplmentary Material for XML.docx]

*Supplementary Material*

**Structural Insights into the Host-Guest Complexation between *β*-Cyclodextrin and Bio-conjugatable Adamantane Derivatives**

Table of Contents

[Figure S1. The ^1^H NMR spectrum (500 MHz, DMSO-*d*_6_, room temperature) of 1. 3](#_Toc69730051)

[Figure S2. The ^1^H NMR spectrum (500 MHz, DMSO-*d*_6_, room temperature) of 2. 3](#_Toc69730052)

[Figure S3. The ^1^H NMR spectrum (500 MHz, DMSO-*d*_6_, room temperature) of 3. 4](#_Toc69730053)

[Figure S4. The ^1^H NMR spectrum (500 MHz, DMSO-*d*_6_, room temperature) of 4. 4](#_Toc69730054)

[Figure S5. The ^1^H NMR spectrum (500 MHz, DMSO-*d*_6_, room temperature) of 5. 5](#_Toc69730055)

[Figure S6. The ^1^H NMR spectrum (500 MHz, DMSO-*d*_6_, room temperature) of 6. 5](#_Toc69730056)

[Figure S7. The ^1^H NMR spectra (500 MHz, DMSO-*d*_6_, room temperature) of 1, β-CD and adm-1-OH. 6](#_Toc69730057)

[Figure S8. The ^1^H NMR spectra (500 MHz, DMSO-*d*_6_, room temperature) of 2, β-CD and adm-2-OH. 6](#_Toc69730058)

[Figure S9. The ^1^H NMR spectra (500 MHz, DMSO-*d*_6_, room temperature) of 3, β-CD and adm-1-NH_2_. 7](#_Toc69730059)

[Figure S10. The ^1^H NMR spectra (500 MHz, DMSO-*d*_6_, room temperature) of 4, β-CD and adm-1-COOH. 7](#_Toc69730060)

[Figure S11. The ^1^H NMR spectra (500 MHz, DMSO-*d*_6_, room temperature) of 5, β-CD and adm-1,3-diCOOH. 8](#_Toc69730061)

[Figure S12. The ^1^H NMR spectra (500 MHz, DMSO-*d*_6_, room temperature) of 6, β-CD and adm-1,3-diCH_2_COOH. 8](#_Toc69730062)

[Figure S13. (a) 2D NOESY spectra (500 MHz, DMSO-*d*_6_, room temperature) of 1 with (b) expansion of significant regions showing interactions between adm-1-OH and β-CD. 9](#_Toc69730063)

[Figure S14. (a) 2D NOESY spectra (500 MHz, DMSO-*d*_6_, room temperature) of 2 with (b) expansion of significant regions showing interactions between adm-2-OH and β-CD. 9](#_Toc69730064)

[Figure S15. (a) 2D NOESY spectra (500 MHz, DMSO-*d*_6_, room temperature) of 3 with (b) expansion of significant regions showing interactions between adm-1-NH_2_ and β-CD. 9](#_Toc69730065)

[Figure S16. (a) 2D NOESY spectra (500 MHz, DMSO-*d*_6_, room temperature) of 4 with (b) expansion of significant regions showing interactions between adm-1-NH_2_ and β-CD. 10](#_Toc69730066)

[Figure S17. (a) 2D NOESY spectra (500 MHz, DMSO-*d*_6_, room temperature) of 5 with (b) expansion of significant regions showing interactions between adm-1,3-diCOOH and β-CD. 10](#_Toc69730067)

[Figure S18. (a) 2D NOESY spectra (500 MHz, DMSO-*d*_6_, room temperature) of 6 with (b) expansion of significant regions showing interactions between adm-1,3-diCH_2_COOH and β-CD. 10](#_Toc69730068)

[Figure S19. (a) TG traces of adm-2-OH, β-CD and 2. (b) A comparison of the DSC-TGA curves of 2 and its subcomponents β-CD and adm-2-OH, showing the melting point alteration upon complex formation. 11](#_Toc69730069)

[Figure S20. (a) TG traces of adm-1-NH_2_, β-CD and 3. (b) A comparison of the DSC-TGA curves of 3 and its subcomponents β-CD and adm-1-NH_2_, showing the melting point alteration upon complex formation. 11](#_Toc69730070)

[Figure S21. (a) TG traces of adm-1-COOH, β-CD and 4. (b) A comparison of the DSC-TGA curves of 4 and its subcomponents β-CD and adm-1-COOH, showing the melting point alteration upon complex formation. 11](#_Toc69730071)

[Figure S22. (a) TG traces of adm-1,3-diCOOH, β-CD and 5. (b) A comparison of the DSC-TGA curves of 5 and its subcomponents β-CD and adm-1,3-diCOOH, showing the melting point alteration upon complex formation. 12](#_Toc69730072)

[Figure S23. (a) TG traces of adm-1,3-diCH_2_COOH, β-CD and 6. (b) A comparison of the DSC-TGA curves of 6 its subcomponents β-CD and adm-1,3-diCH_2_COOH, showing the melting point alteration upon complex formation. 12](#_Toc69730073)

[Figure S24. The X-ray crystal structure of 3 showing the diverse guest orientations in the inclusion complexes. The guests are presented as a space-filling model. The hydrogen atoms are omitted for clarity. Color codes: O (brown-red), N (blue), C (black). 13](#_Toc69730074)

[Figure S25. The crystal packing diagram of 6 (along *c* direction). Color codes: O (red) and C (gray). 13](#_Toc69730075)

[Table S1. Chemical shifts of adamantane derivative 1 with/without complexation with β-CD (recorded in DMSO-*d*_6_ with TMS as the internal standard) for comparison. 14](#_Toc69730076)

[Table S2. Chemical shifts of adamantane derivative 2 with/without complexation with β-CD (recorded in DMSO-*d*_6_ with TMS as the internal standard) for comparison. 14](#_Toc69730077)

[Table S3. Chemical shifts of adamantane derivative 3 with/without complexation with β-CD (recorded in DMSO-*d*_6_ with TMS as the internal standard) for comparison. 14](#_Toc69730078)

[Table S4. Chemical shifts of adamantane derivative 4 with/without complexation with β-CD (recorded in DMSO-*d*_6_ with TMS as the internal standard) for comparison. 14](#_Toc69730079)

[Table S5. Chemical shifts of adamantane derivative 5 with/without complexation with β-CD (recorded in DMSO-*d*_6_ with TMS as the internal standard) for comparison. 14](#_Toc69730080)

[Table S6. Chemical shifts of adamantane derivative 6 with/without complexation with β-CD (recorded in DMSO-*d*_6_ with TMS as the internal standard) for comparison. 15](#_Toc69730081)

[Table S7. Chemical shifts of β-CD in 1 with/without complexation with adamantane derivative (recorded in DMSO-*d*_6_ with TMS as the internal standard) for comparison. 15](#_Toc69730082)

[Table S8. Chemical shifts of β-CD in 2 with/without complexation with adamantane derivative (recorded in DMSO-*d*_6_ with TMS as the internal standard) for comparison. 15](#_Toc69730083)

[Table S9. Chemical shifts of β-CD in 3 with/without complexation with adamantane derivative (recorded in DMSO-*d*_6_ with TMS as the internal standard) for comparison. 16](#_Toc69730084)

[Table S10. Chemical shifts of β-CD in 4 with/without complexation with adamantane derivative (recorded in DMSO-*d*_6_ with TMS as the internal standard) for comparison. 16](#_Toc69730085)

[Table S11. Chemical shifts of β-CD in 5 with/without complexation with adamantane derivative (recorded in DMSO-*d*_6_ with TMS as the internal standard) for comparison. 16](#_Toc69730086)

[Table S12. Chemical shifts of β-CD in 6 with/without complexation with adamantane derivative (recorded in DMSO-*d*_6_ with TMS as the internal standard) for comparison. 17](#_Toc69730087)


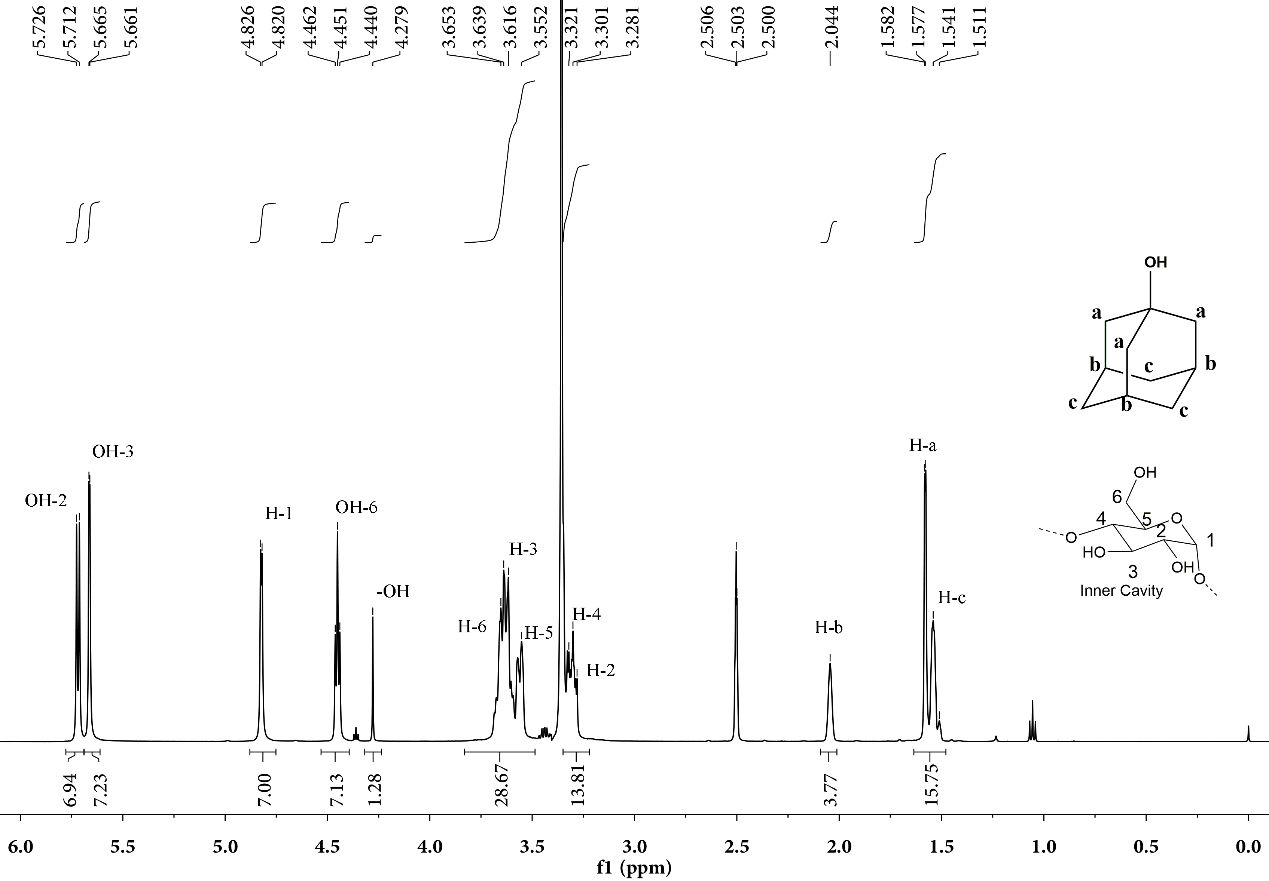


Figure S1. The ^1^H NMR spectrum (500 MHz, DMSO-*d*_6_, room temperature) of 1.


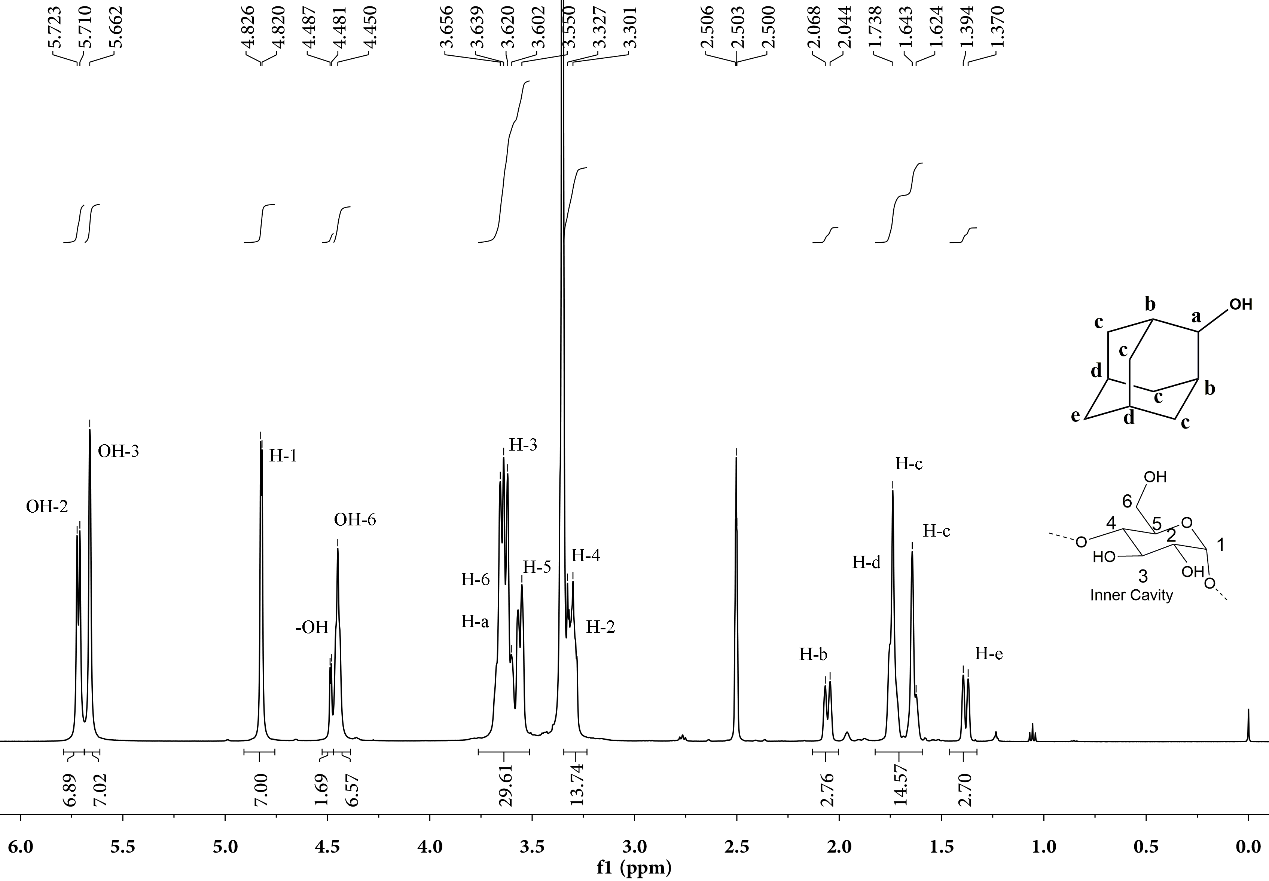


Figure S2. The ^1^H NMR spectrum (500 MHz, DMSO-*d*_6_, room temperature) of 2.


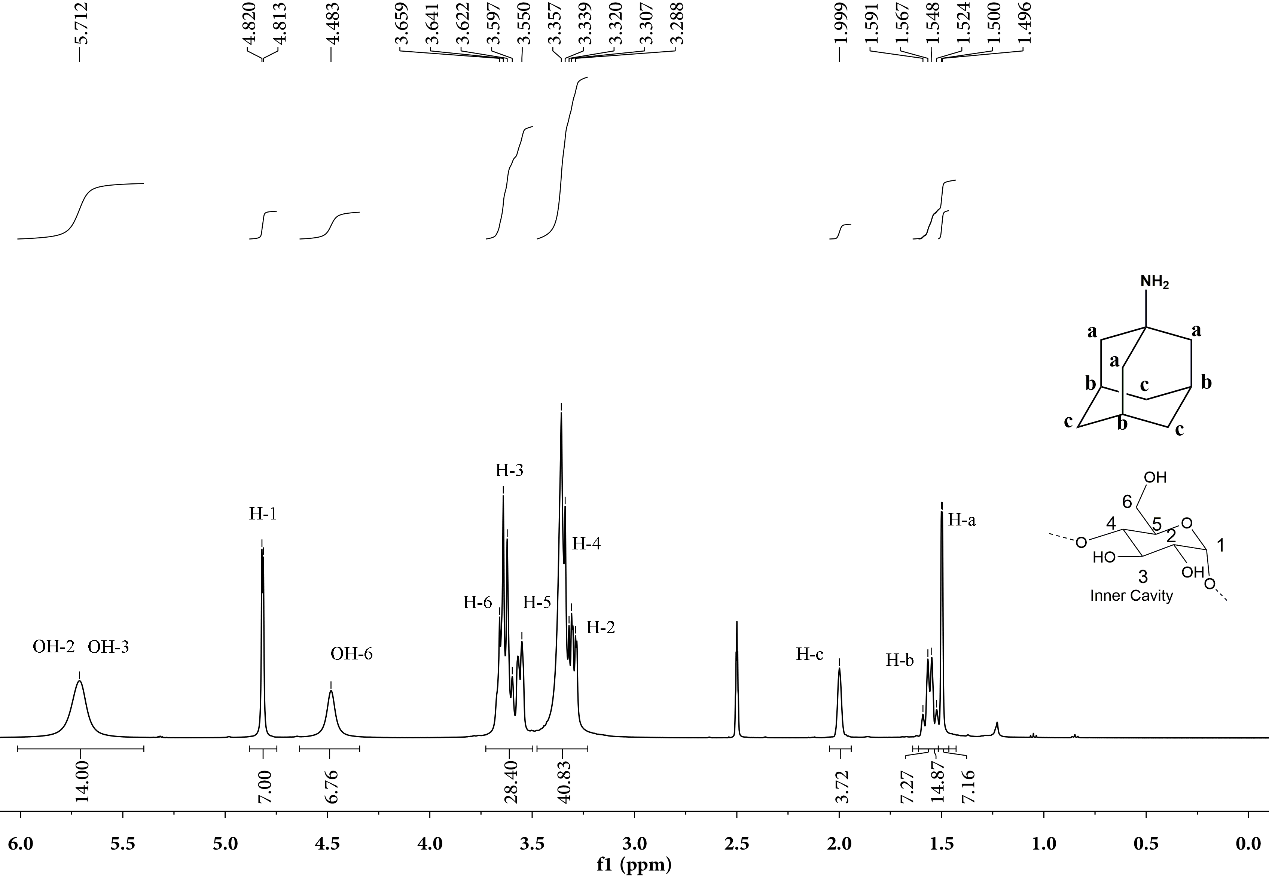


Figure S3. The ^1^H NMR spectrum (500 MHz, DMSO-*d*_6_, room temperature) of 3.


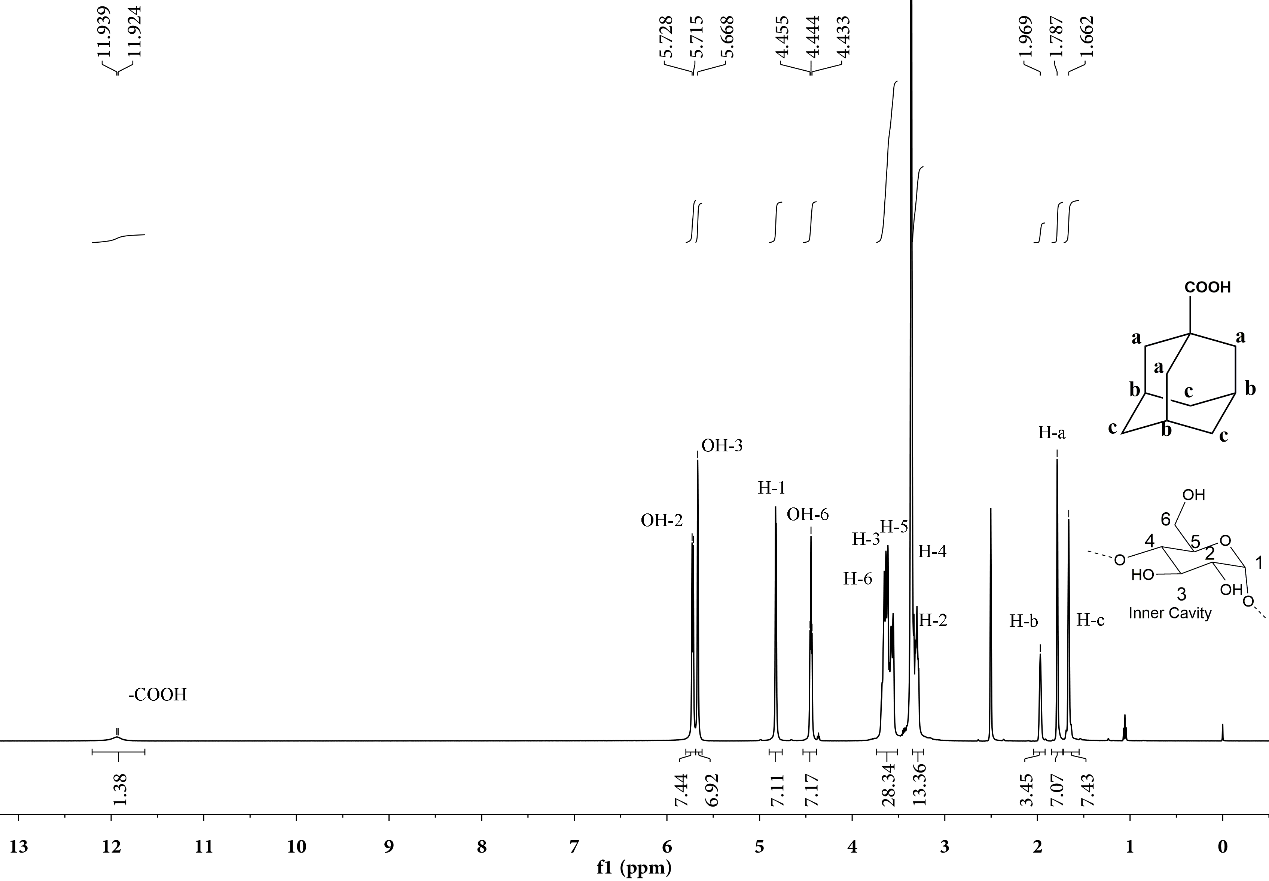


Figure S4. The ^1^H NMR spectrum (500 MHz, DMSO-*d*_6_, room temperature) of 4.


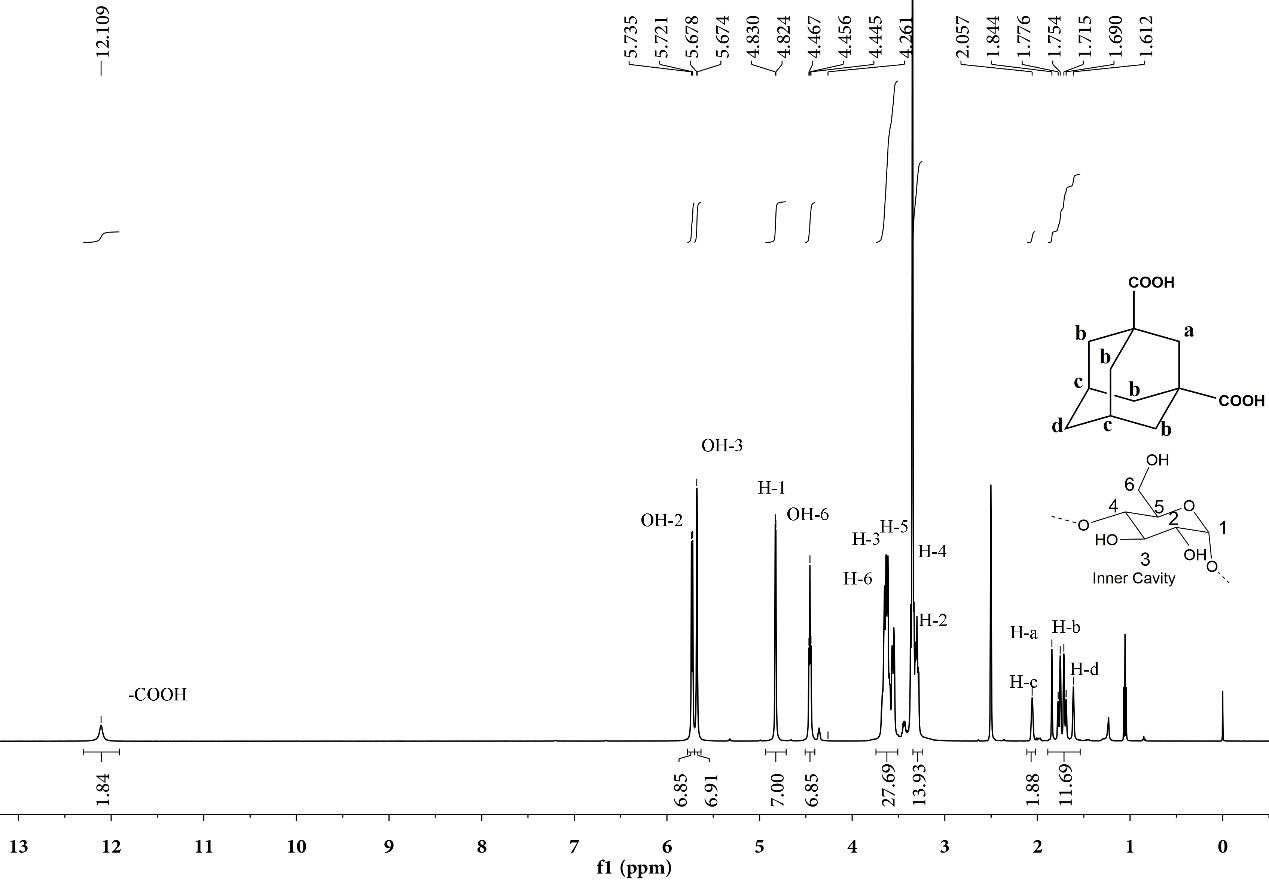


Figure S5. The ^1^H NMR spectrum (500 MHz, DMSO-*d*_6_, room temperature) of 5.


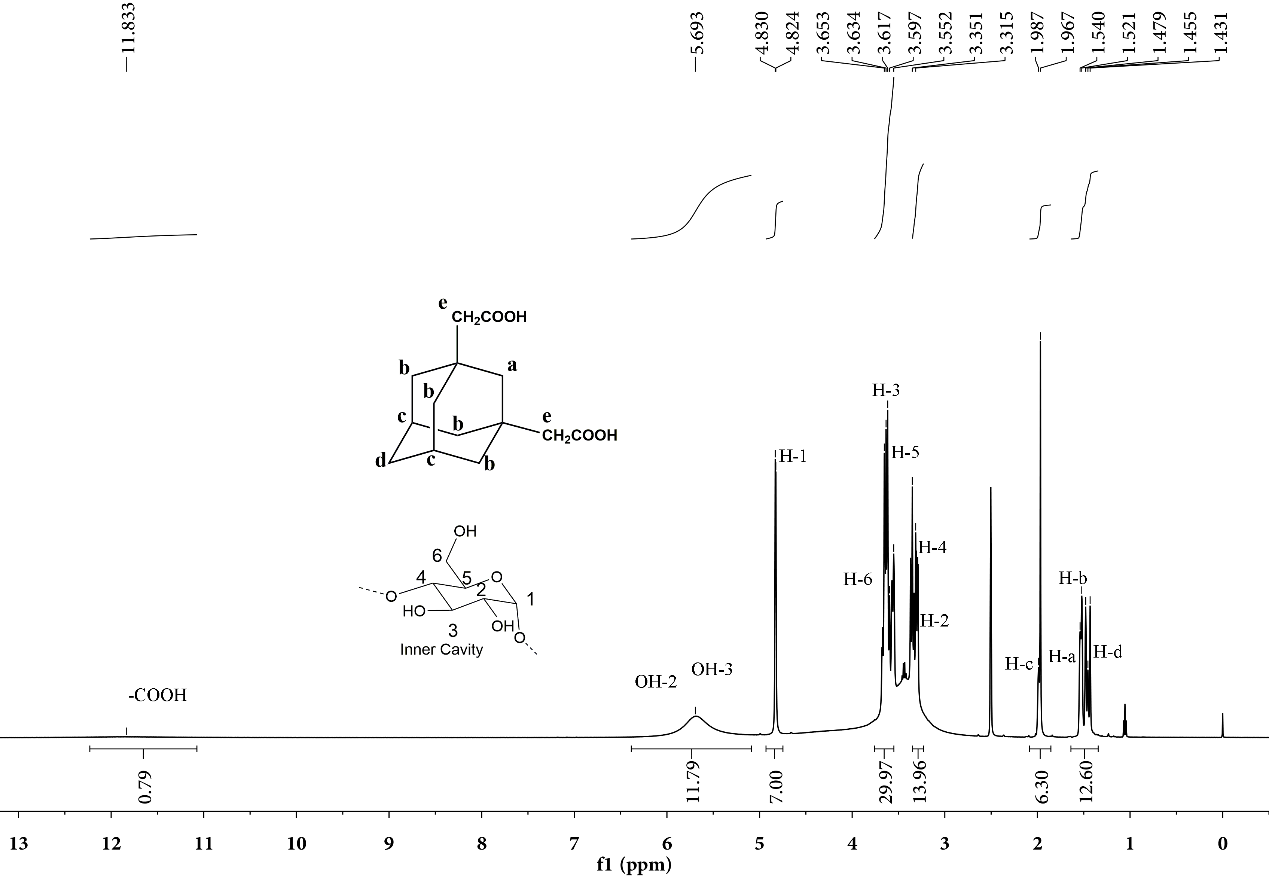


Figure S6. The ^1^H NMR spectrum (500 MHz, DMSO-*d*_6_, room temperature) of 6.


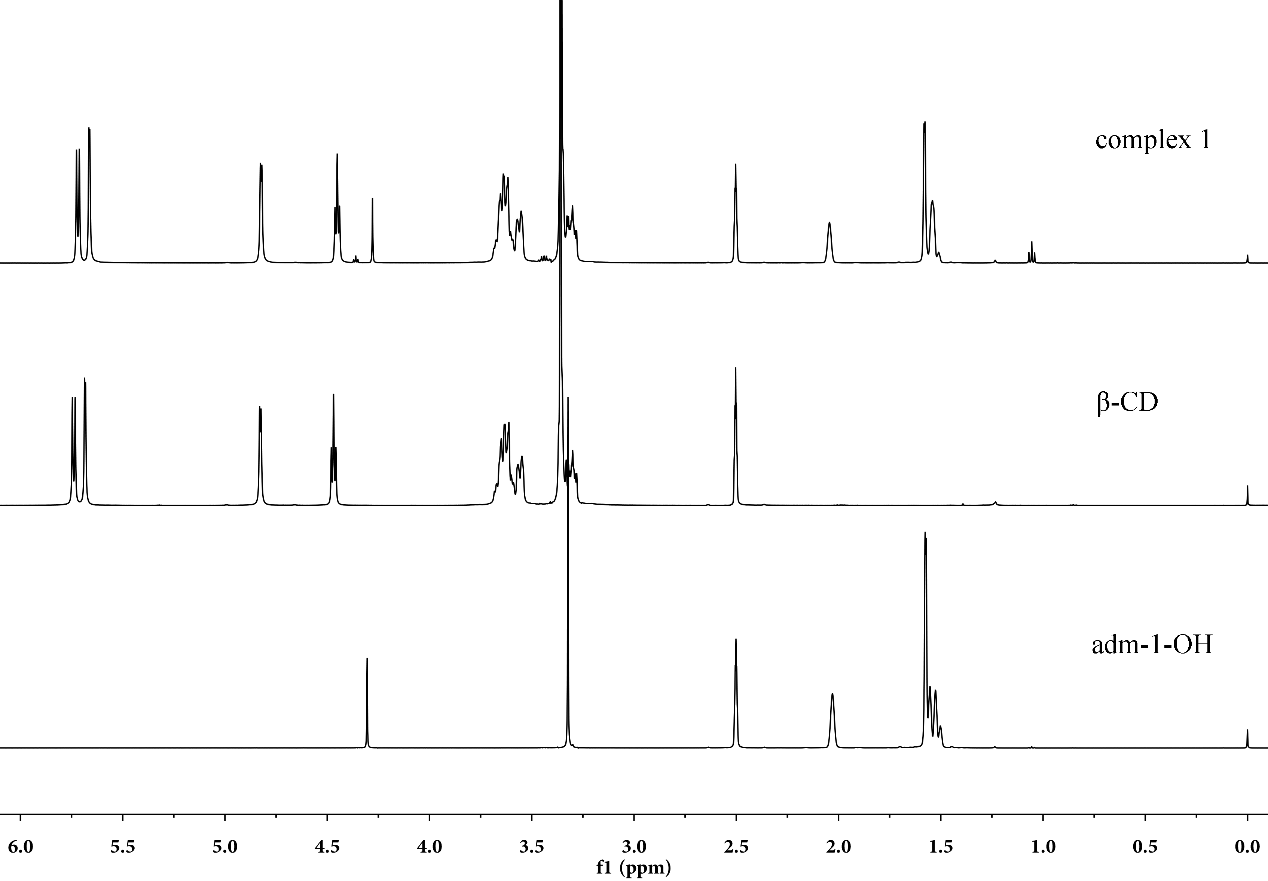


Figure S7. The ^1^H NMR spectra (500 MHz, DMSO-*d*_6_, room temperature) of 1, β-CD and adm-1-OH.


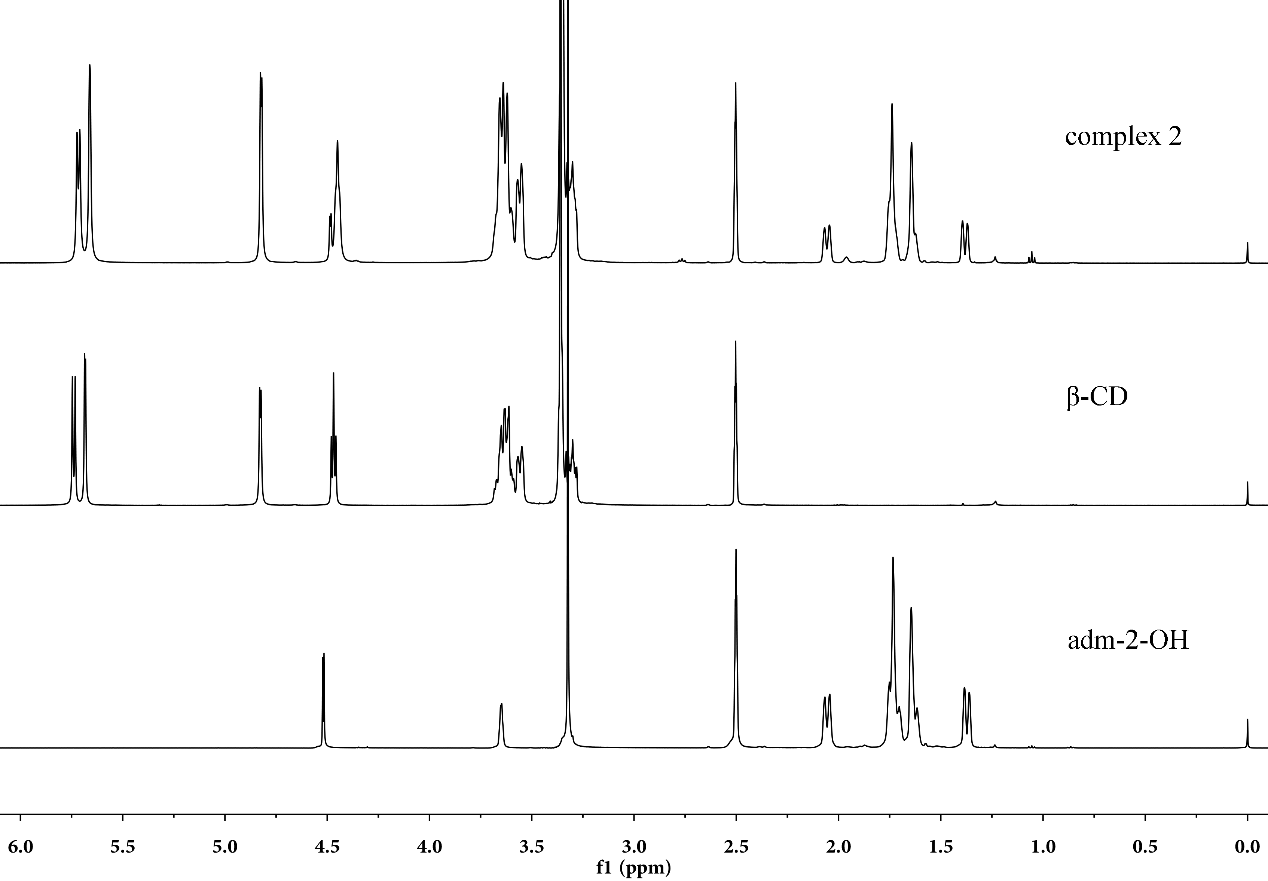


Figure S8. The ^1^H NMR spectra (500 MHz, DMSO-*d*_6_, room temperature) of 2, β-CD and adm-2-OH.


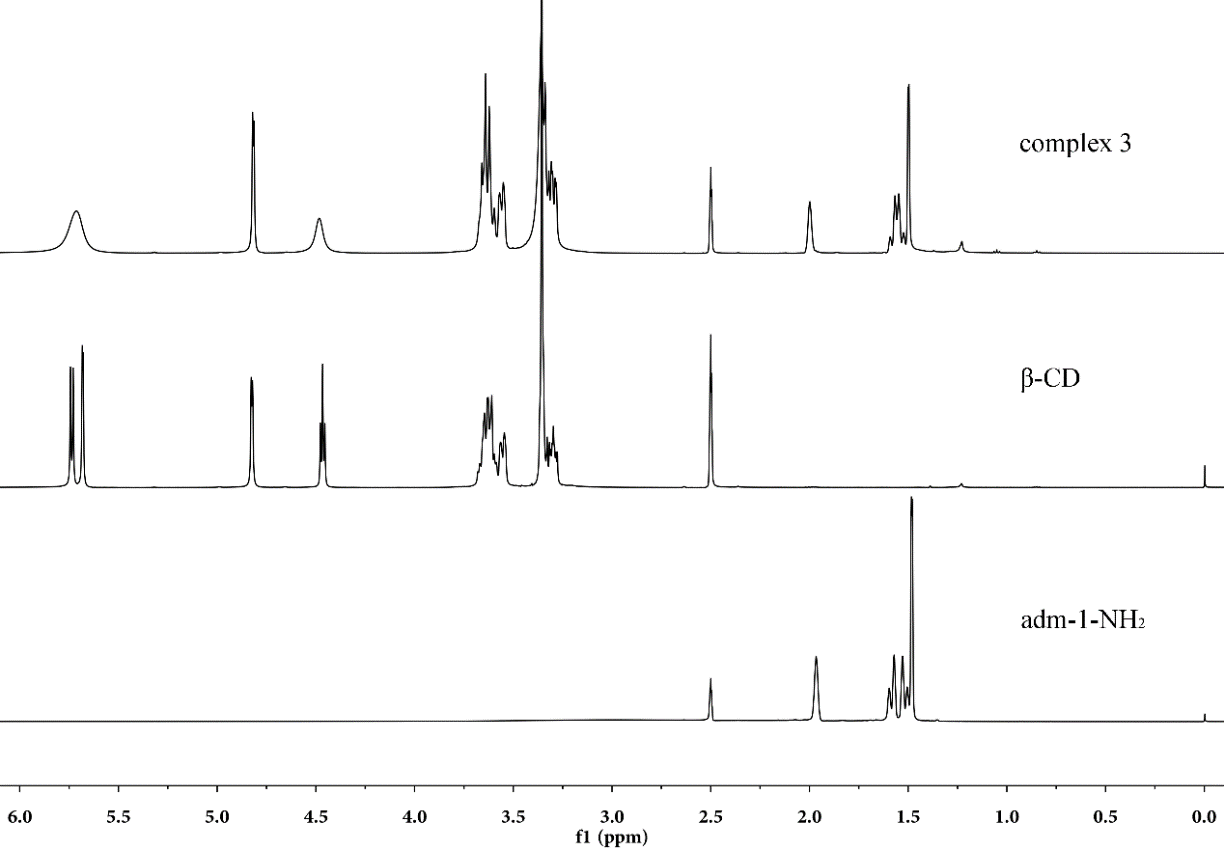


Figure S9. The ^1^H NMR spectra (500 MHz, DMSO-*d*_6_, room temperature) of 3, β-CD and adm-1-NH_2_.


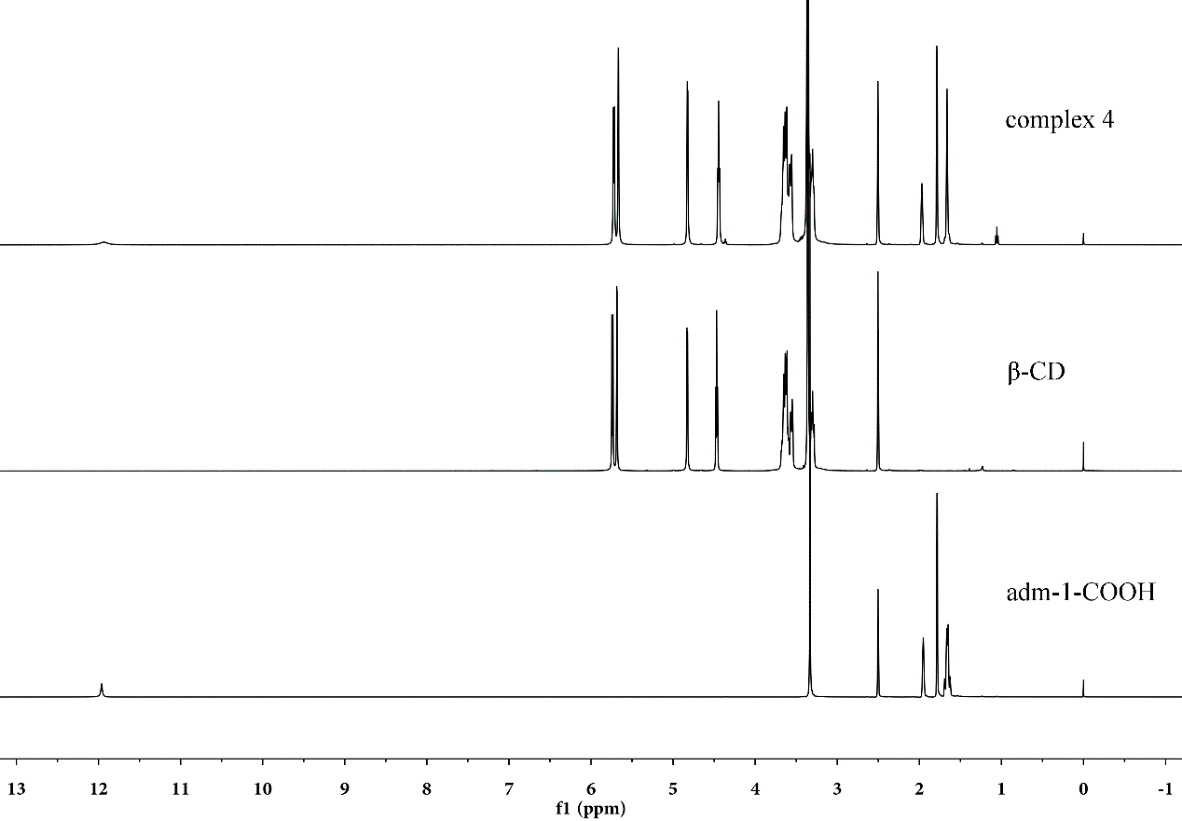


Figure S10. The ^1^H NMR spectra (500 MHz, DMSO-*d*_6_, room temperature) of 4, β-CD and adm-1-COOH.


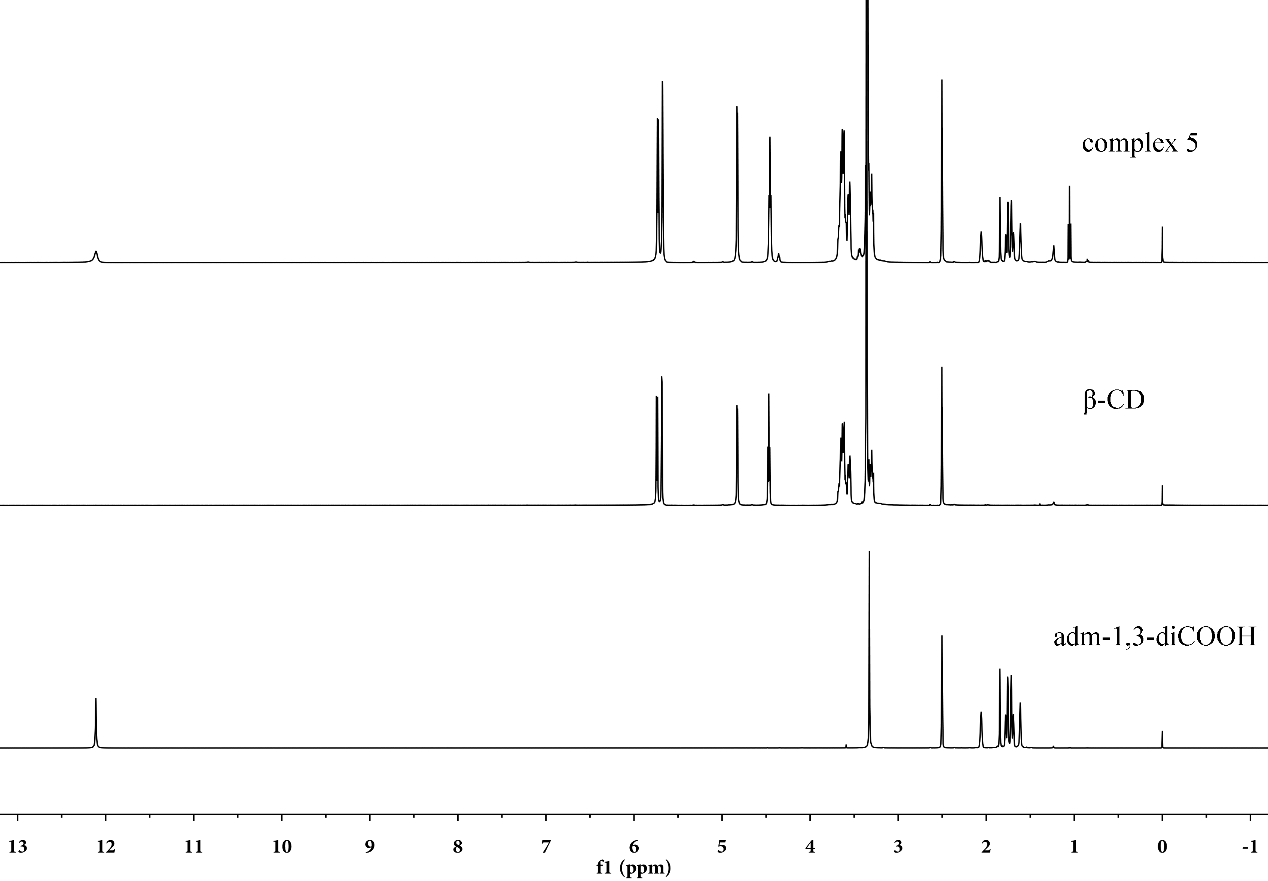


Figure S11. The ^1^H NMR spectra (500 MHz, DMSO-*d*_6_, room temperature) of 5, β-CD and adm-1,3-diCOOH.


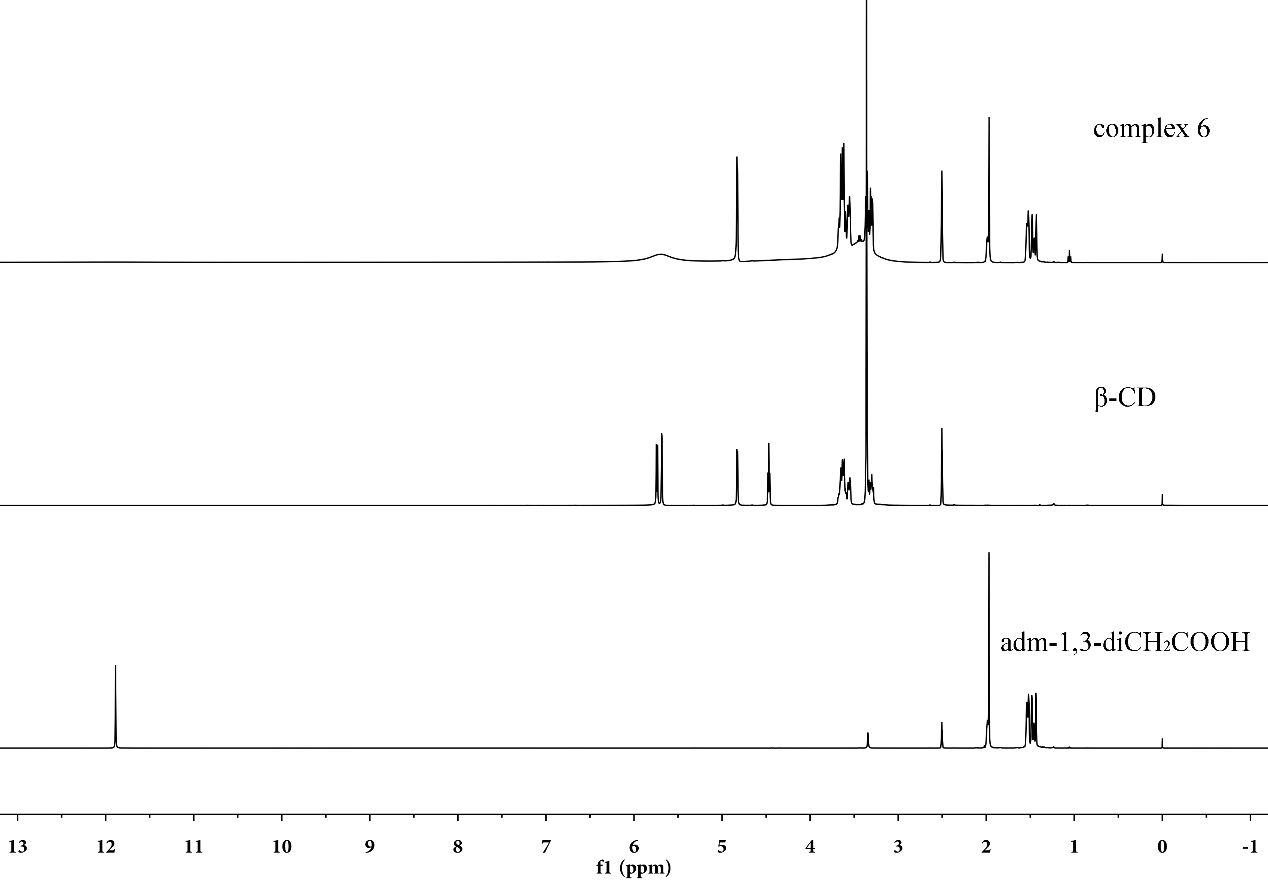


Figure S12. The ^1^H NMR spectra (500 MHz, DMSO-*d*_6_, room temperature) of 6, β-CD and adm-1,3-diCH_2_COOH.


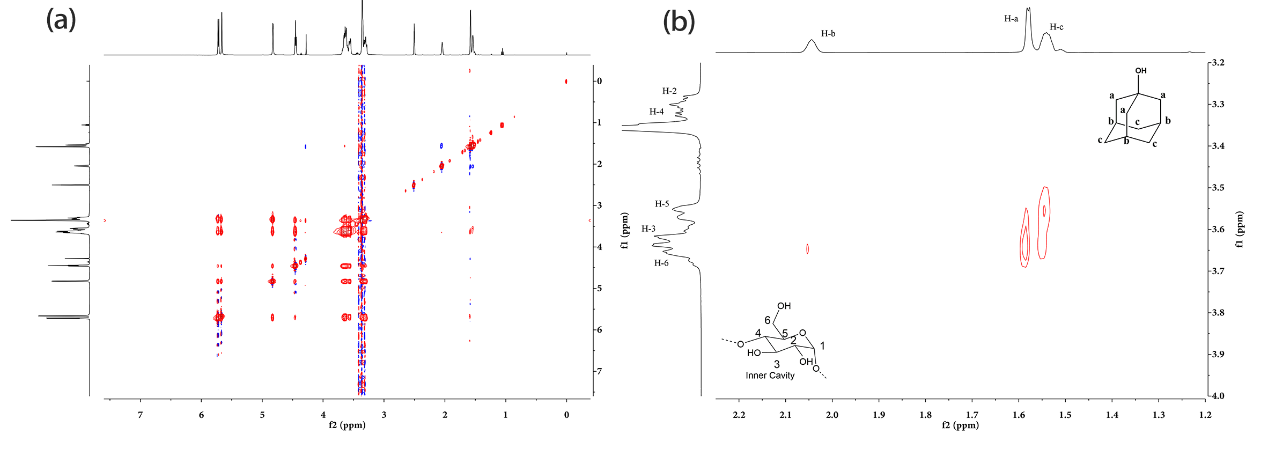


**Figure S13.** (**a**) 2D NOESY spectra (500 MHz, DMSO-*d*_6_, room temperature) of 1 with (b) expansion of significant regions showing interactions between adm-1-OH and β-CD.


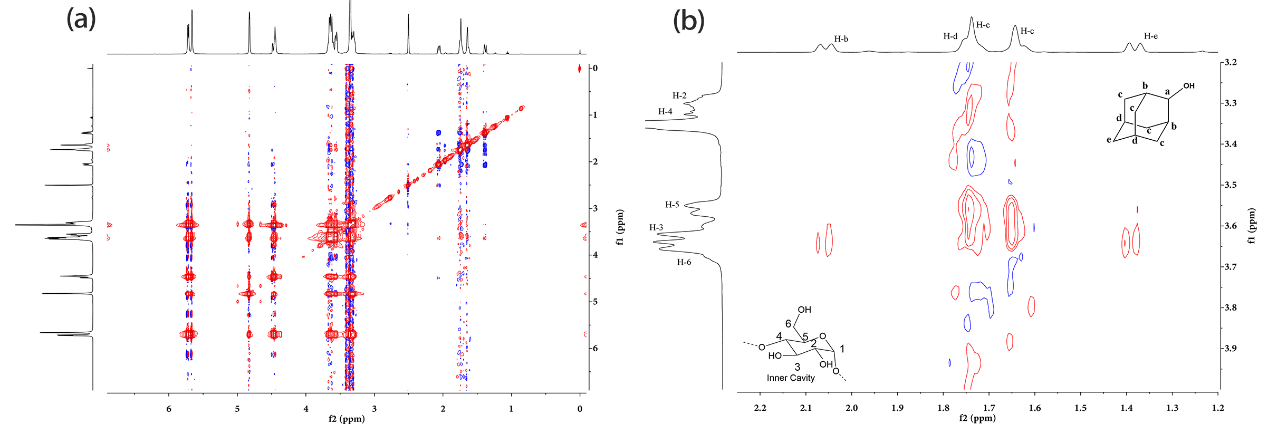


Figure S14. (a) 2D NOESY spectra (500 MHz, DMSO-*d*_6_, room temperature) of 2 with (b) expansion of significant regions showing interactions between adm-2-OH and β-CD.


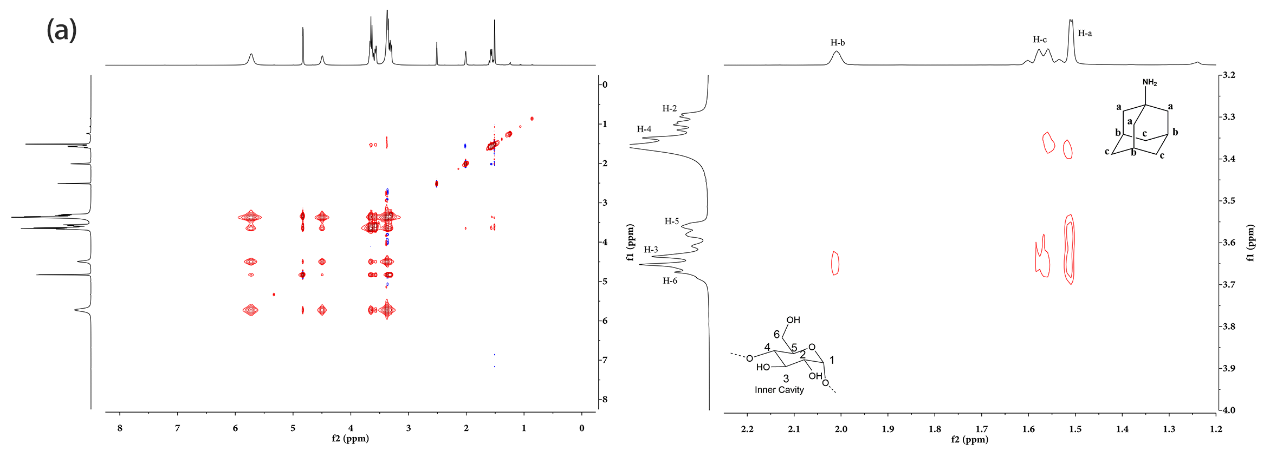


Figure S15. (a) 2D NOESY spectra (500 MHz, DMSO-*d*_6_, room temperature) of 3 with (b) expansion of significant regions showing interactions between adm-1-NH_2_ and β-CD.


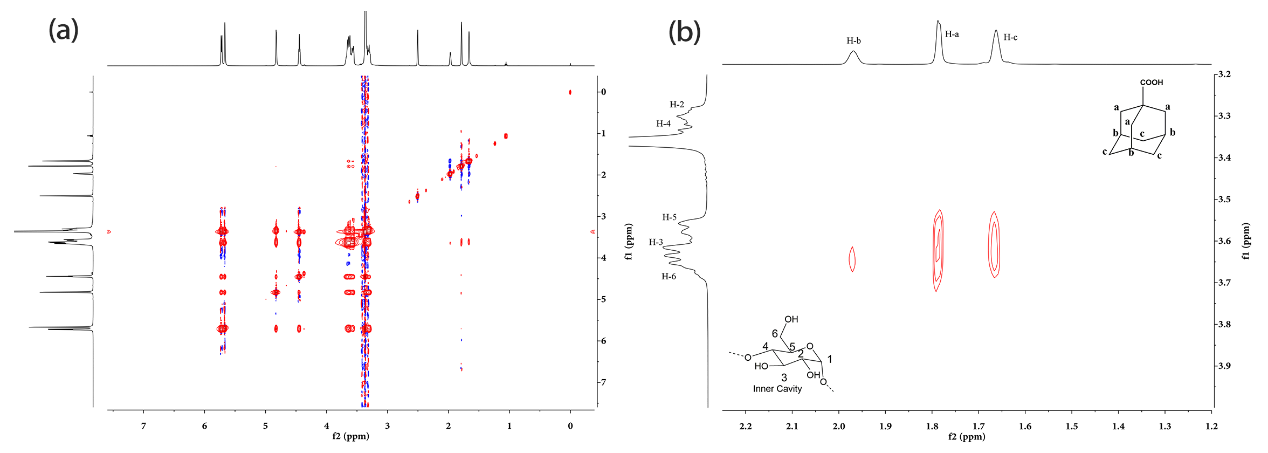


Figure S16. (a) 2D NOESY spectra (500 MHz, DMSO-*d*_6_, room temperature) of 4 with (b) expansion of significant regions showing interactions between adm-1-NH_2_ and β-CD.


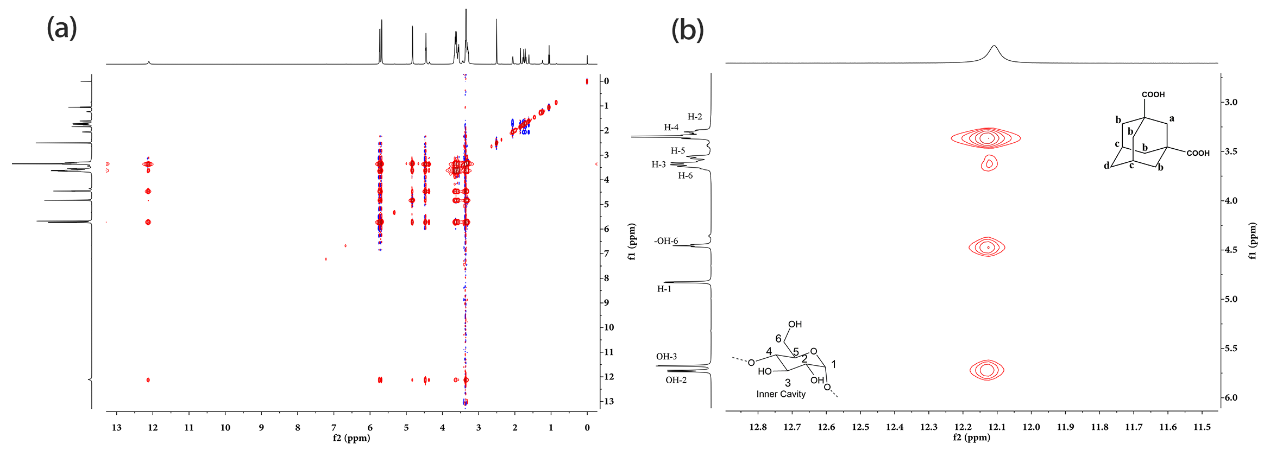


Figure S17. (a) 2D NOESY spectra (500 MHz, DMSO-*d*_6_, room temperature) of 5 with (b) expansion of significant regions showing interactions between adm-1,3-diCOOH and β-CD.


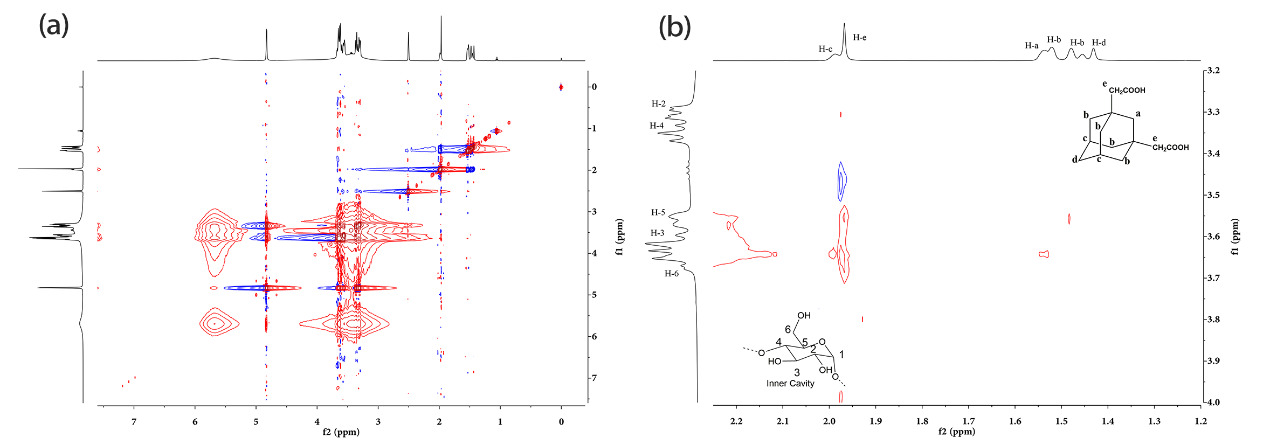


Figure S18. (a) 2D NOESY spectra (500 MHz, DMSO-*d*_6_, room temperature) of 6 with (b) expansion of significant regions showing interactions between adm-1,3-diCH_2_COOH and β-CD.


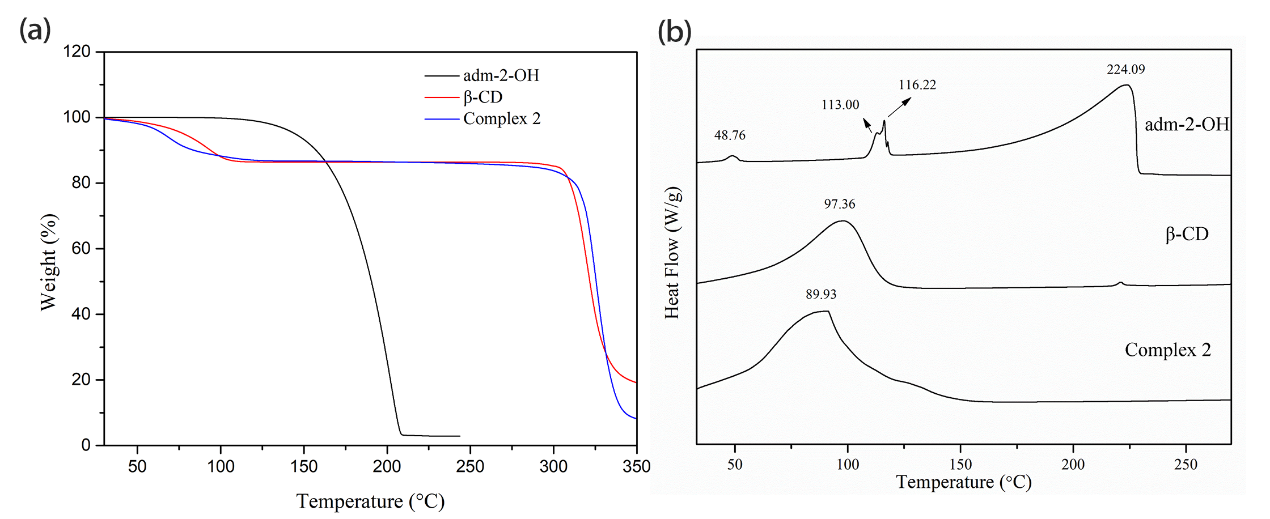


Figure S19. (a) TG traces of adm-2-OH, β-CD and 2. (b) A comparison of the DSC-TGA curves of 2 and its subcomponents β-CD and adm-2-OH, showing the melting point alteration upon complex formation.


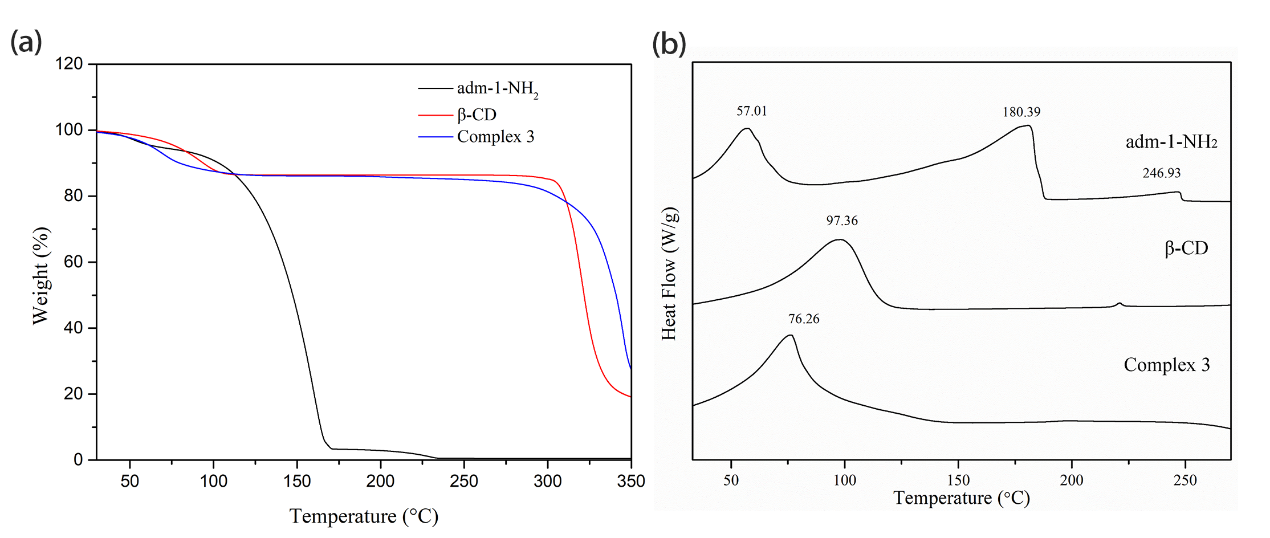


Figure S20. (a) TG traces of adm-1-NH_2_, β-CD and 3. (b) A comparison of the DSC-TGA curves of 3 and its subcomponents β-CD and adm-1-NH_2_, showing the melting point alteration upon complex formation.


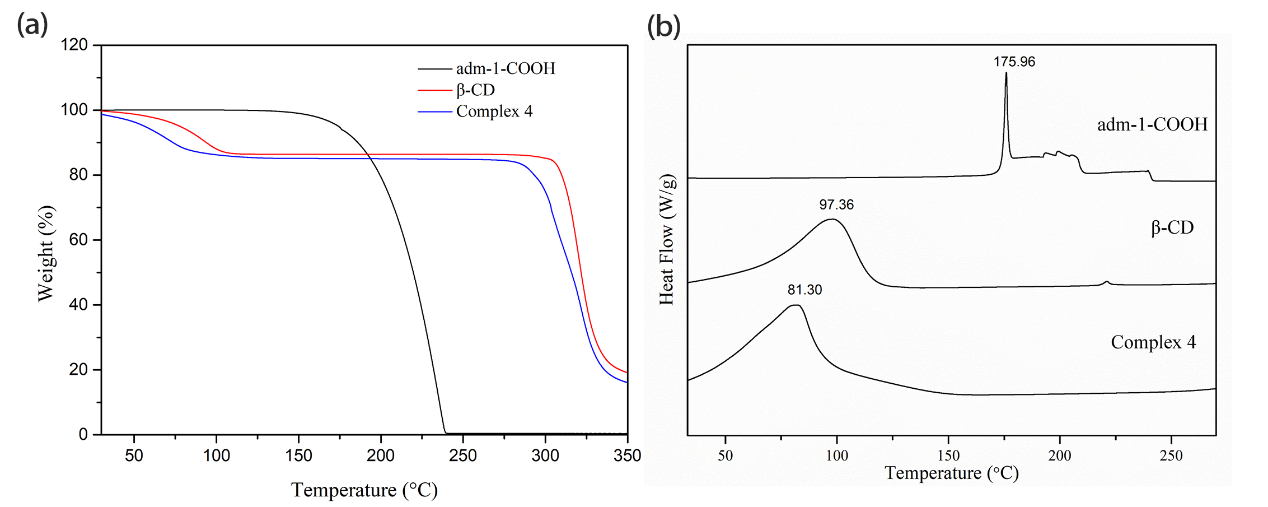


Figure S21. (a) TG traces of adm-1-COOH, β-CD and 4. (b) A comparison of the DSC-TGA curves of 4 and its subcomponents β-CD and adm-1-COOH, showing the melting point alteration upon complex formation.


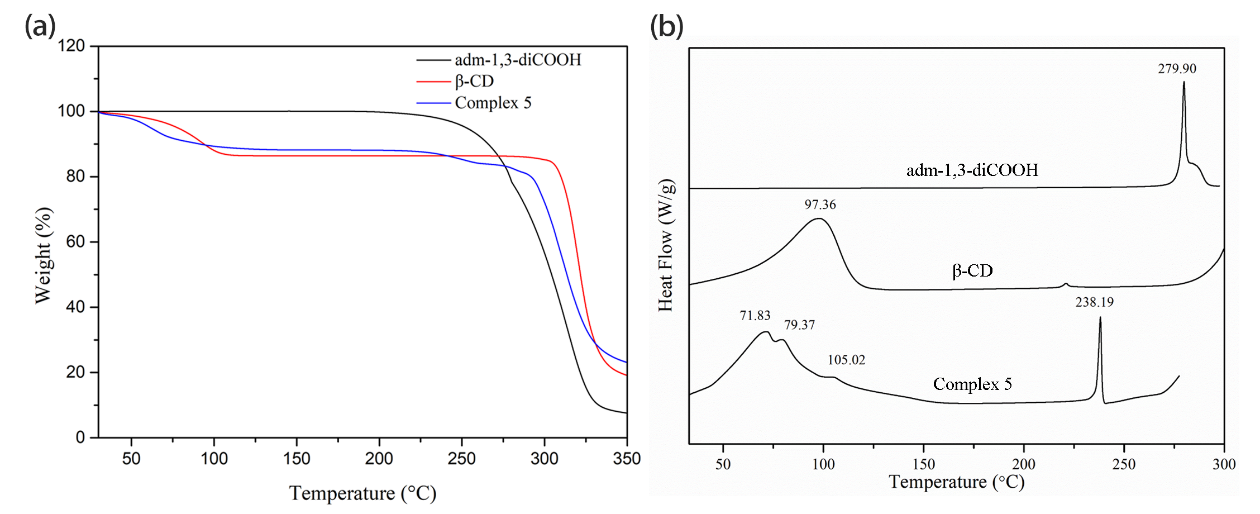


Figure S22. (a) TG traces of adm-1,3-diCOOH, β-CD and 5. (b) A comparison of the DSC-TGA curves of 5 and its subcomponents β-CD and adm-1,3-diCOOH, showing the melting point alteration upon complex formation.


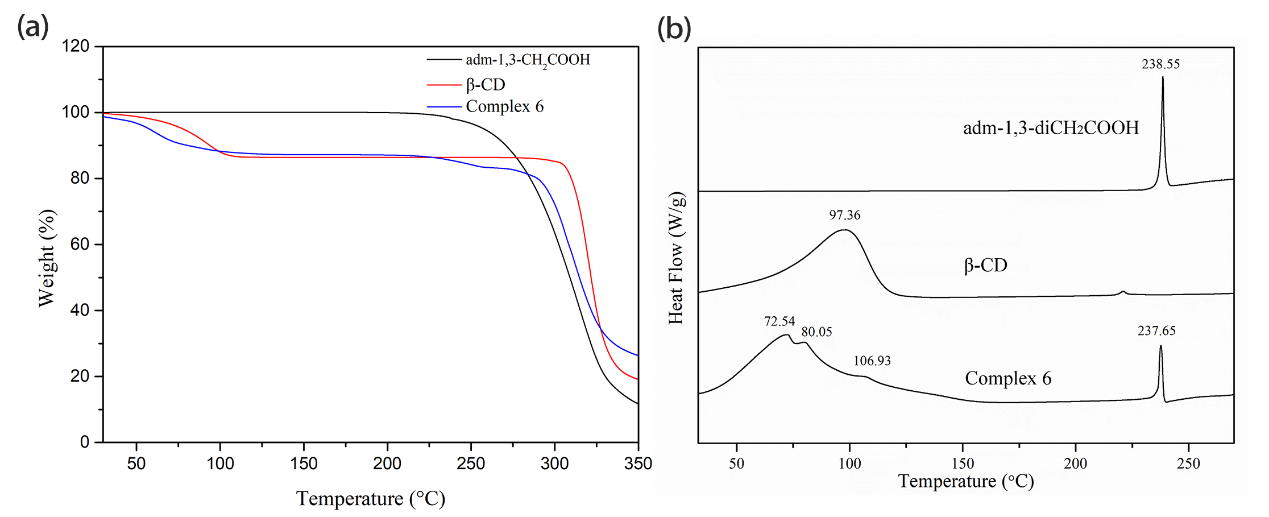


Figure S23. (a) TG traces of adm-1,3-diCH_2_COOH, β-CD and 6. (b) A comparison of the DSC-TGA curves of 6 its subcomponents β-CD and adm-1,3-diCH_2_COOH, showing the melting point alteration upon complex formation.


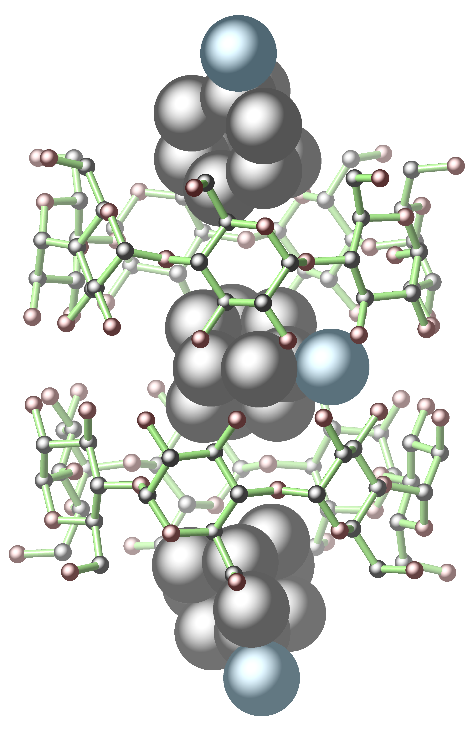


Figure S24. The X-ray crystal structure of 3 showing the diverse guest orientations in the inclusion complexes. The guests are presented as a space-filling model. The hydrogen atoms are omitted for clarity. Color codes: O (brown-red), N (blue), C (black).


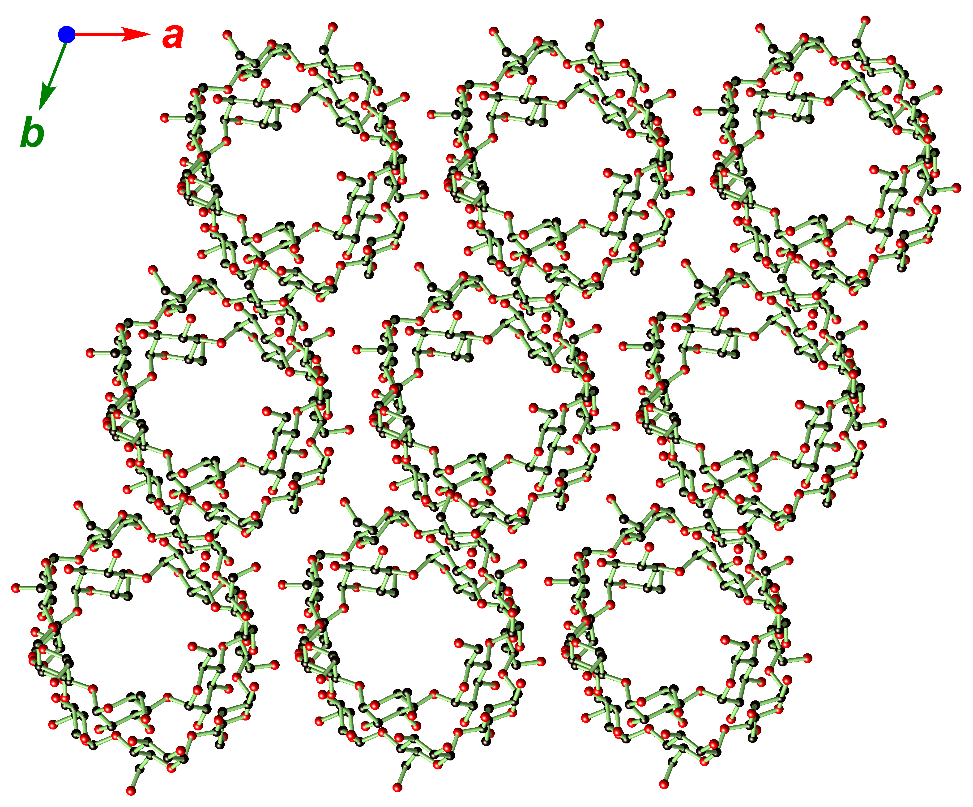


Figure S25. The crystal packing diagram of 6 (along *c* direction). Color codes: O (red) and C (gray).

Table S1. Chemical shifts of adamantane derivative 1 with/without complexation with β-CD (recorded in DMSO-*d*_6_ with TMS as the internal standard) for comparison.

| **Adamantane Derivative 1** | **Hydrogen** | **δ_free state_** | **δ_complexed state_** | **Δδ^a^** | **Remark** |
| --- | --- | --- | --- | --- | --- |
|  | H-a | 1.5739 | 1.5796 | 0.0057 |  |
|  | H-b | 2.0296 | 2.0442 | 0.0146 |  |
|  | H-c | 1.5394 | 1.5406 | 0.0012 | no split |
|  | -OH | 4.3049 | 4.2786 | −0.0263 |  |
|  |  |  |  |  |  |

a. Δδ = δ_complexed state_ − δ_free state._

Table S2. Chemical shifts of adamantane derivative 2 with/without complexation with β-CD (recorded in DMSO-*d*_6_ with TMS as the internal standard) for comparison.

| **Adamantane Derivative 2** | **Hydrogen** | **δ_free state_** | **δ_complexed state_** | **Δδ^a^** | **Remark** |
| --- | --- | --- | --- | --- | --- |
|  | H-a | 3.6495 | 3.6019 | −0.0476 |  |
|  | H-b | 2.0549 | 2.0561 | 0.0012 |  |
|  | H-c | 1.6889 | 1.6905 | 0.0016 |  |
|  | H-d | 1.7029 | Merge with peak of 1.7381 | 0.0352 |  |
|  | H-e | 1.3723 | 1.3818 | 0.0095 |  |
|  | -OH | 4.5192 | 4.4843 | −0.0349 |  |

a. Δδ = δ_complexed state_ − δ_free state._

Table S3. Chemical shifts of adamantane derivative 3 with/without complexation with β-CD (recorded in DMSO-*d*_6_ with TMS as the internal standard) for comparison.

| **Adamantane Derivative 3** | **Hydrogen** | **δ_free state_** | **δ_complexed state_** | **Δδ^a^** | **Remark** |
| --- | --- | --- | --- | --- | --- |
|  | H-a | 1.4813 | 1.4983 | 0.017 |  |
|  | H-b | 1.9655 | 1.9987 | 0.0332 |  |
|  | H-c | 1.5492 | 1.5573 | 0.0081 |  |
|  | -NH_2_ | broad | disappear |  |  |
|  |  |  |  |  |  |

a. Δδ = δ_complexed state_ − δ_free state._

Table S4. Chemical shifts of adamantane derivative 4 with/without complexation with β-CD (recorded in DMSO-*d*_6_ with TMS as the internal standard) for comparison.

| **Adamantane Derivative 4** | **Hydrogen** | **δ_free state_** | **δ_complexed state_** | **Δδ^a^** | **Remark** |
| --- | --- | --- | --- | --- | --- |
|  | H-a | 1.7836 | 1.7874 | 0.0038 |  |
|  | H-b | 1.9526 | 1.9688 | 0.0162 |  |
|  | H-c | 1.6588 | 1.6624 | 0.0036 | no split |
|  | -COOH | 11.9616 | 11.9389 | −0.0227 |  |
|  |  |  |  |  |  |

a. Δδ = δ_complexed state_ − δ_free state._

Table S5. Chemical shifts of adamantane derivative 5 with/without complexation with β-CD (recorded in DMSO-*d*_6_ with TMS as the internal standard) for comparison.

| **Adamantane Derivative 5** | **Hydrogen** | **δ_free state_** | **δ_complexed state_** | **Δδ^a^** | **Remark** |
| --- | --- | --- | --- | --- | --- |
|  | H-a | 1.8453 | 1.8439 | −0.0014 |  |
|  | H-b | 1.7361 | 1.7343 | −0.0018 |  |
|  | H-c | 2.0573 | 2.0570 | −0.0003 |  |
|  | H-d | 1.6132 | 1.6119 | −0.0013 |  |
|  | -COOH | 12.1117 | 12.1087 | −0.0030 |  |

a. Δδ = δ_complexed state_ − δ_free state._

Table S6. Chemical shifts of adamantane derivative 6 with/without complexation with β-CD (recorded in DMSO-*d*_6_ with TMS as the internal standard) for comparison.

| **Adamantane Derivative 6** | **Hydrogen** | **δ_free state_** | **δ_complexed state_** | **Δδ^a^** | **Remark** |
| --- | --- | --- | --- | --- | --- |
|  | H-a | 1.5392 | 1.5396 | 0.0004 | Merge with peak H-b |
|  | H-b | 1.5011 | 1.5003 | −0.0008 |  |
|  | H-c | 1.9866 | 1.9873 | 0.0007 |  |
|  | H-d | 1.4352 | 1.4311 | −0.0041 |  |
|  | H-e | 1.9692 | 1.9674 | −0.0018 |  |
|  | -COOH | 11.8876 | 11.8325 | −0.0551 |  |

**a.** Δδ = δ_complexed state_ − δ_free state_

Table S7. Chemical shifts of β-CD in 1 with/without complexation with adamantane derivative (recorded in DMSO-*d*_6_ with TMS as the internal standard) for comparison.

| **Complex 1** | **Hydrogen** | **δ_free state_** | **δ_complexed state_** | **Δδ^a^** | **Remark** |
| --- | --- | --- | --- | --- | --- |
|  | H-1 | 4.8270 | 4.8231 | −0.0039 |  |
|  | H-2 | / | / |  |  |
|  | H-3 | 3.6117 | 3.6161 | 0.0044 |  |
|  | H-4 | / | / |  |  |
|  | H-5 | 3.5581 | 3.5614 | 0.0033 |  |
|  | H-6 | / | / |  |  |
|  | 2-OH | 5.7392 | 5.7189 | −0.0203 |  |
|  | 3-OH | 5.6841 | 5.6632 | −0.0209 |  |
|  | 6-OH | 4.4690 | 4.4511 | −0.0179 |  |

a. Δδ = δ_complexed state_ − δ_free state._

Table S8. Chemical shifts of β-CD in 2 with/without complexation with adamantane derivative (recorded in DMSO-*d*_6_ with TMS as the internal standard) for comparison.

| **Complex 2** | **Hydrogen** | **δ_free state_** | **δ_complexed state_** | **Δδ^a^** | **Remark** |
| --- | --- | --- | --- | --- | --- |
|  | H-1 | 4.8270 | 4.8231 | −0.0039 |  |
|  | H-2 | / | / |  |  |
|  | H-3 | 3.6117 | 3.6196 | 0.0079 |  |
|  | H-4 | / | / |  |  |
|  | H-5 | 3.5581 | 3.5597 | 0.0016 |  |
|  | H-6 | / | / |  |  |
|  | 2-OH | 5.7392 | 5.7165 | −0.0227 |  |
|  | 3-OH | 5.6841 | 5.6616 | −0.0225 | Doblet to siglet |
|  | 6-OH | 4.4690 | 4.4497 | −0.0193 | Triplet to pro-siglet peak |

a. Δδ = δ_complexed state_ − δ_free state._

Table S9. Chemical shifts of β-CD in 3 with/without complexation with adamantane derivative (recorded in DMSO-*d*_6_ with TMS as the internal standard) for comparison.

| **Complex 3** | **Hydrogen** | **δ_free state_** | **δ_complexed state_** | **Δδ^a^** | **Remark** |
| --- | --- | --- | --- | --- | --- |
|  | H-1 | 4.8270 | 4.8168 | −0.0102 |  |
|  | H-2 | / | / |  |  |
|  | H-3 | 3.6117 | 3.6220 | 0.0103 |  |
|  | H-4 | / | / |  |  |
|  | H-5 | 3.5581 | 3.5598 | 0.0017 |  |
|  | H-6 | / | / |  |  |
|  | 2-OH | 5.7392 | 5.7116 | −0.0276 | Fused into one peak |
|  | 3-OH | 5.6841 | 5.7116 | 0.0275 |  |
|  | 6-OH | 4.4690 | 4.4829 | 0.0139 | Doublet to singlet |

a. Δδ = δ_complexed state_ − δ_free state._

Table S10. Chemical shifts of β-CD in 4 with/without complexation with adamantane derivative (recorded in DMSO-*d*_6_ with TMS as the internal standard) for comparison.

| **Complex 4** | **Hydrogen** | **δ_free state_** | **δ_complexed state_** | **Δδ^a^** | **Remark** |
| --- | --- | --- | --- | --- | --- |
|  | H-1 | 4.8270 | 4.8237 | −0.0033 |  |
|  | H-2 | / | / |  |  |
|  | H-3 | 3.6117 | 3.6145 | 0.0028 |  |
|  | H-4 | / | / |  |  |
|  | H-5 | 3.5581 | 3.5689 | 0.0108 |  |
|  | H-6 | / | / |  |  |
|  | 2-OH | 5.7392 | 5.7214 | −0.0178 |  |
|  | 3-OH | 5.6841 | 5.6677 | −0.0164 | Doublet peak to singlet |
|  | 6-OH | 4.4690 | 4.4445 | −0.0245 |  |

a. Δδ = δ_complexed state_ − δ_free state._

Table S11. Chemical shifts of β-CD in 5 with/without complexation with adamantane derivative (recorded in DMSO-*d*_6_ with TMS as the internal standard) for comparison.

| **Complex 5** | **Hydrogen** | **δ_free state_** | **δ_complexed state_** | **Δδ^a^** | **Remark** |
| --- | --- | --- | --- | --- | --- |
|  | H-1 | 4.8270 | 4.8272 | 0.0002 |  |
|  | H-2 | / | / |  |  |
|  | H-3 | 3.6117 | 3.6134 | 0.0017 |  |
|  | H-4 | / | / |  |  |
|  | H-5 | 3.5581 | 3.5597 | 0.0016 |  |
|  | H-6 | / | / |  |  |
|  | 2-OH | 5.7392 | 5.7283 | −0.0109 | No peak shape change |
|  | 3-OH | 5.6841 | 5.6759 | −0.0082 |  |
|  | 6-OH | 4.4690 | 4.4559 | −0.0131 |  |

a. Δδ = δ_complexed state_ − δ_free state._

Table S12. Chemical shifts of β-CD in 6 with/without complexation with adamantane derivative (recorded in DMSO-*d*_6_ with TMS as the internal standard) for comparison.

| **Complex 6** | **Hydrogen** | **δ_free state_** | **δ_complexed state_** | **Δδ^a^** | **Remark** |
| --- | --- | --- | --- | --- | --- |
|  | H-1 | 4.8270 | 4.8272 | 0.0002 |  |
|  | H-2 | / | / |  |  |
|  | H-3 | 3.6117 | 3.6168 | 0.0051 |  |
|  | H-4 | / | / |  |  |
|  | H-5 | 3.5581 | 3.5616 | 0.0035 |  |
|  | H-6 | / | / |  |  |
|  | 2-OH | 5.7392 | 5.6927 | −0.0465 | Fused into one peak |
|  | 3-OH | 5.6841 | 5.6927 | 0.0086 |  |
|  | 6-OH | 4.4690 | vanish | 0.0002 |  |

a. Δδ = δ_complexed state_ − δ_free state._
